# DNA barcoding of the National Museum of Natural History reptile tissue holdings raises concerns about the use of natural history collections and the responsibilities of scientists in the molecular age

**DOI:** 10.1371/journal.pone.0264930

**Published:** 2022-03-04

**Authors:** Daniel G. Mulcahy, Roberto Ibáñez, Cesar A. Jaramillo, Andrew J. Crawford, Julie M. Ray, Steve W. Gotte, Jeremy F. Jacobs, Addison H. Wynn, Gracia P. Gonzalez-Porter, Roy W. McDiarmid, Ronald I. Crombie, George R. Zug, Kevin de Queiroz

**Affiliations:** 1 Division of Amphibians and Reptiles, Department of Vertebrate Zoology, National Museum of Natural History, Washington, DC, United States of America; 2 Smithsonian Tropical Research Institute, Panama City, Republic of Panama; 3 Sistema Nacional de Investigación, SENACYT, Panamá City, República de Panamá; 4 Departamento de Zoología, Universidad de Panamá, Panamá City, República de Panamá; 5 Departamento de Histología y Neuroanatomía, Facultad de Medicina, Universidad de Panamá, Panamá City, República de Panamá; 6 Department of Biological Sciences, Museo de Historia Natural C.J. Marinkelle, Universidad de los Andes, Bogotá, Colombia; 7 Department of Biology, University of Nevada, Reno, Nevada, United States of America; 8 Bioma, Mexico City, Mexico; 9 Department of Herpetology, California Academy of Sciences, San Francisco, California, United States of America; Nanjing Agricultural University, CHINA

## Abstract

Natural history collections are essential to a wide variety of studies in biology because they maintain large collections of specimens and associated data, including genetic material (e.g., tissues) for DNA sequence data, yet they are currently under-funded and collection staff have high workloads. With the advent of aggregate databases and advances in sequencing technologies, there is an increased demand on collection staff for access to tissue samples and associated data. Scientists are rapidly developing large DNA barcode libraries, DNA sequences of specific genes for species across the tree of life, in order to document and conserve biodiversity. In doing so, mistakes are made. For instance, inconsistent taxonomic information is commonly taken from different lending institutions and deposited in data repositories, such as the Barcode of Life Database (BOLD) and GenBank, despite explicit disclaimers regarding the need for taxonomic verification by the lending institutions. Such errors can have profound effects on subsequent research based on these mis-labelled sequences in data repositories. Here, we present the production of a large DNA barcode library of reptiles from the National Museum of Natural History tissue holdings. The library contains 2,758 sequences (2,205 *COI* and 553 *16S*) from 2260 specimens (four crocodilians, 37 turtles, and 2,219 lizards, including snakes), representing 583 named species, from 52 countries. In generating this library, we noticed several common mistakes made by scientists depositing DNA barcode data in public repositories (e.g., BOLD and GenBank). Our goal is to raise awareness of these concerns and offer advice to avoid such mistakes in the future to maintain accurate DNA barcode libraries to properly document Earth’s biodiversity.

## Introduction

Natural history collections are fundamental to a wide variety of studies in biology. Taxonomic and systematic scientists rely on museum specimens, and their results (the species and clades recognized) underlie studies in nearly every field in biology [1]. Consequently, natural history museums play a crucial role in documenting biodiversity by building, curating, and maintaining specimen collections. The responsibilities of collections staff include (to name a few) registering incoming collections, cataloguing specimens, organizing and reorganizing the collection (both to reflect taxonomic changes and to deal with space constraints), and maintaining a variety of specimen collections, including large series within a species, essential for characterizing intra-specific variation. Collections range from typical whole-specimen, cleared and stained, and osteology preparations to images, sound recordings, tissues, and the associated databases, and staff are responsible for providing access to researchers around the world, including online, by hosting visitors, and shipping loans. Additionally, collections staff and researchers must now adhere to the recent Nagoya Protocols set forth by the Convention on Biological Diversity regarding the access and benefits sharing of genetic resources.

Regrettably, natural history collections currently face many threats, from collections work not being a popular career to budget cuts and threats of closure, especially in the case of university museums, where financially more attractive options exist (e.g., closure of Monroe Natural History Museum [2] to make room for sports facilities). Consequently, natural history collections commonly become casualties of dwindling infrastructure support, as exemplified by the recent tragic event in Brazil [3]. Compounding these threats, technological advances (e.g., scanning and imaging techniques, molecular methods) place increasing demands on collections and their staff. Additionally, continual species discoveries [4], improved estimates of phylogenetic relationships, and advances in obtaining DNA from formalin-fixed specimens [5] have resulted in an enormous increase in the number of specimens that are usable for DNA sequencing, which increase the workload for collections staff. The modernization and expansion of collection databases also places new demands on collections staff, including such tasks as georeferencing, digitization, standardizing vocabulary for aggregate search-engines, and maintaining active hyperlinks to related databases.

Museum specimens commonly serve as sources of molecular sequence data stored in public repositories such as GenBank and the Barcode of Life Database (BOLD). The molecular sequence data are often generated and deposited by external (to the holding museum) researchers. Increasingly, specimen data are accessed through aggregate databases, such as the Global Biodiversity Information Facility (GBIF) and Global Genome Biodiversity Network (GGBN). The coordination between natural history collections, aggregate databases, and data repositories is the responsibility of all parties involved, including museum collection staff and database managers. However, most of the responsibility falls on the researchers, because both museums and data repositories (e.g., BOLD, GenBank) are often unable to keep up with rapidly changing taxonomies and often only the original submitters are allowed to make changes. In museum collections, taxonomic changes not only require updating the name in the collection database, but also specimen labels, and it also commonly requires physically rearranging the collections to maintain a logical and efficient order, which may involve tens to thousands of specimens for a given species.

The Smithsonian Institution’s National Museum of Natural History (NMNH) maintains the largest natural history collection in the world [6], including one of the largest collections of amphibians and reptiles in the world and an ever-increasing tissue collection (>18,000 samples from over 14,000 specimens). Although not all of the tissue collection is currently searchable on the public webpage, the staff has been fulfilling tissue requests for several decades and is working to digitize the entire tissue collection. Like many other institutions, the NMNH has specific loan policies regarding the loan, dissemination, and use of specimens and associated data (e.g., taxonomy, collector, locality, DNA sequences, etc.), and borrowers of material must agree to follow those policies as a condition of the loan. When researchers do not follow the policies, their actions often confound matters by perpetuating outdated information, increasing the workload for an already under-staffed and otherwise strained collection staff.

Here, we report on the production of a *COI* DNA barcode library [7] focused on reptile tissue holdings in the NMNH Division of Amphibians and Reptiles, but also including tissues of specimens housed in other institutions. We provide a brief overview of these DNA barcodes, the taxa, and geographic regions they represent, noting cases in which species exhibit interesting patterns of genetic variation. Some of these cases may represent natural variation in widespread species, while others may signify species complexes in need of taxonomic revision. In other cases, with closely related species, or parthenogenetic clonal species resulting from interspecific hybridization, we find nearly identical DNA barcode sequences.

In depositing our data in BOLD and GenBank, we discovered several common problems concerning the terms of collections use and the deposition of molecular data in public databases. The three most significant were when researchers: a) submitted data accepting taxonomic identifications as received from the lending institution, without considering recent taxonomic changes that may not have been incorporated into the lending museum’s database; b) did not conduct BLAST searches to verify identifications prior to publishing; and c) submitted data for a new species under its previous name (in cases of taxonomic splitting) and failed to update the records when the study was published. Such errors create taxonomic confusion and may be compounded by future studies that use the associated data before the necessary corrections are made. We highlight several cases where these taxonomic errors have profound consequences for the results. We point out other problems in the use of molecular data, recommend procedures to avoid them, and suggest best practices regarding the use of museum specimens and genetic data for museum staff, database managers, editors and reviewers, and most importantly—researchers.

## Materials and methods

### Sampling methods

We subsampled tissue holdings of the NMNH, Department of Vertebrate Zoology, Division of Amphibians and Reptiles, focusing on reptiles. Most tissue samples came from specimens collected by current and previous NMNH researchers, and the voucher specimens were either deposited in the NMNH collection or in other museum collections. The current NMNH was formerly part of the United States National Museum, which was divided into several museums in the mid 1960s [8]. The USNM initials were maintained for historical continuity and are used for citing specimens in the NMNH herpetological collection [9]. In cases where the voucher specimens were lost, destroyed, not collected, or deposited in a different museum, the genetic samples were either associated with USNM Herp Image (USNM-HI) catalogue numbers (when photos were available) or as USNM Herp Tissue (USNM-HT) catalogue numbers. A large number (1050 sequences from 562 specimens) of our BOLD records are in a project in BOLD (SQUAP—Squamates of Panama). For many of the SQUAP records, the tissues and voucher specimens are located in the Collection of Herpetology (CH; former collection of the Círculo Herpetológico de Panamá) at the Smithsonian Tropical Research Institute (STRI; n = 386). For the remaining SQUAP records, either the tissues and voucher specimens are housed in the NMNH collection (n = 152) or the vouchers are housed at either the Museo de Vertebrados, Universidad de Panamá (MVUP; n = 20), the San Diego State Natural History Musuem (SDNHM; n = 2), or the Illinois Natural History Survey collection (INHS; n = 2) and the tissues are in the NMNH collection. The remaining records of this study are contained in a separate project in BOLD (NMNHR—USNM Reptile Tissue Holdings) and most tissue and voucher specimens are housed the NMNH collection (n = 1,502). In some cases, voucher specimens are housed in the Museum of Vertebrate Zoology (MVZ), University of California, Berkeley (n = 68); the California Academy of Sciences (CAS), San Francisco (n = 55); or other institutions and private collections, and the tissues are in the NMNH collection. Our subsampling included 3–5 individuals per species in most cases (when available), with some exceptions in cases of highly variable or widely distributed species (e.g., *Indotyphlops braminus* n = 17; *Hemidactylus frenatus* n = 30; and *Lepidodactylus lugubris* n = 58).

### Molecular methods

Small pieces of liver, muscle, or homogenates from allozyme preparations were extracted using an Auto-Genprep 965 (2011 AutoGen, Inc.), with standard phenol manufacturer protocols, at the Laboratories of Analytical Biology, NMNH. Genomic DNA was eluted in 100 μl of AutoGen R9 re-suspension buffer. We sequenced the DNA barcode in the 5’ region of the *COI* mitochondrial DNA (mtDNA) gene following standard DNA barcoding protocols of the lab in which the data were generated [10]. For some individuals (mostly in SQUAP), we sequenced part of the mtDNA *16S* ribosomal gene as an additional marker. Specific primer information can be found in BOLD and GenBank for each individual sequence. All DNA barcode records are publicly available in BOLD [upon publication] under the projects NMNHR and SQUAP, and are deposited in GenBank (BioProject PRJNA439252 for NMNHR, *COI*: MH273113–MH274806, *16S*: MH308378–MH308388; and BioProject PRJNA437653 for SQUAP, *COI*: KX459694, KX459696, MH139922–MH140427; *16S*: KX283341–42, MH140469–MH141008). Voucher information is provided for each sequence in BOLD and GenBank, although a minimal set of metadata was provided to these public databases. More detailed information on individual specimens is available from the institutions where the tissues and/or vouchers are catalogued, but note that museum taxonomic identifications may be different from ours in GenBank and BOLD for reasons discussed herein. We strove to associate the currently recognized species names with our sequence data and relied on the Reptile Database [11] for this purpose. In cases where specimens could not be identified to species, only the “genus” is provided and Taxonomy Notes are included in the BOLD records where appropriate. “Families” follow BOLD taxonomy.

### Data analyses and quality control

For data analyses and quality control of raw sequence data we used methods previously outlined [10]. We did not rely entirely on museum (or collector) taxonomic identifications. Instead, we used museum identifications as a starting point, updated identifications based on known taxonomic changes, and used the Barcode Index Numbers (BINs) system [12] and exploratory neighbor-joining (NJ) trees, including data from specimens previously submitted to BOLD and GenBank when available, to assist in specimen identifications [13, 14]. When sequences from specimens originally identified as members of different species were placed in the same BIN (we use “BIN” for both the index number and its corresponding cluster of sequences) or were intermingled among putative clades in our NJ trees, we re-examined those specimens to confirm their IDs. When sequences from specimens originally identified as members of the same species were placed in different BINs, or formed divergent clades (or a clade and a paraphyletic group) in our NJ trees, we re-examined those specimens to confirm their IDs. If initial identifications were confirmed, we considered those cases to represent potential species complexes, incomplete lineage sorting, or introgression caused by hybridization. We considered these cases in need of further investigation, preferably with nuclear markers. We reviewed the literature on these species groups and point out cases of known paraphyly or other taxonomic issues. We do not propose any taxonomic changes, as those deserve more detailed studies; instead, we highlight potential problems in these groups. We were less concerned about inferred paraphyly or polyphyly for groups traditionally ranked as genera or higher taxonomic categories, because ~655 base-pairs (bp) of *COI* (or ~555 bp of *16S*) are not adequate to confidently reconstruct evolutionary history at these levels, although they have proven useful for species delimitations [12, 13]. We conducted a maximum-likelihood analysis using RAxML (v 8.2.11) [15] using the rapid bootstrap analysis (1000 replicates) followed by a thorough ML search (using the GTRCAT model) for our complete *COI* dataset. All genetic comparisons are based on uncorrected p-distances. For some groups, such as *Anolis*, phrynosomatids, and *Pituophis*, we generated expanded datasets including data in BOLD and GenBank not collected by us. We analyzed these expanded datasets using the BOLD aligner and NJ trees to aid in the identification of our samples and to identify mis-labelled specimens in these databases.

## General results

**Fig 1:**
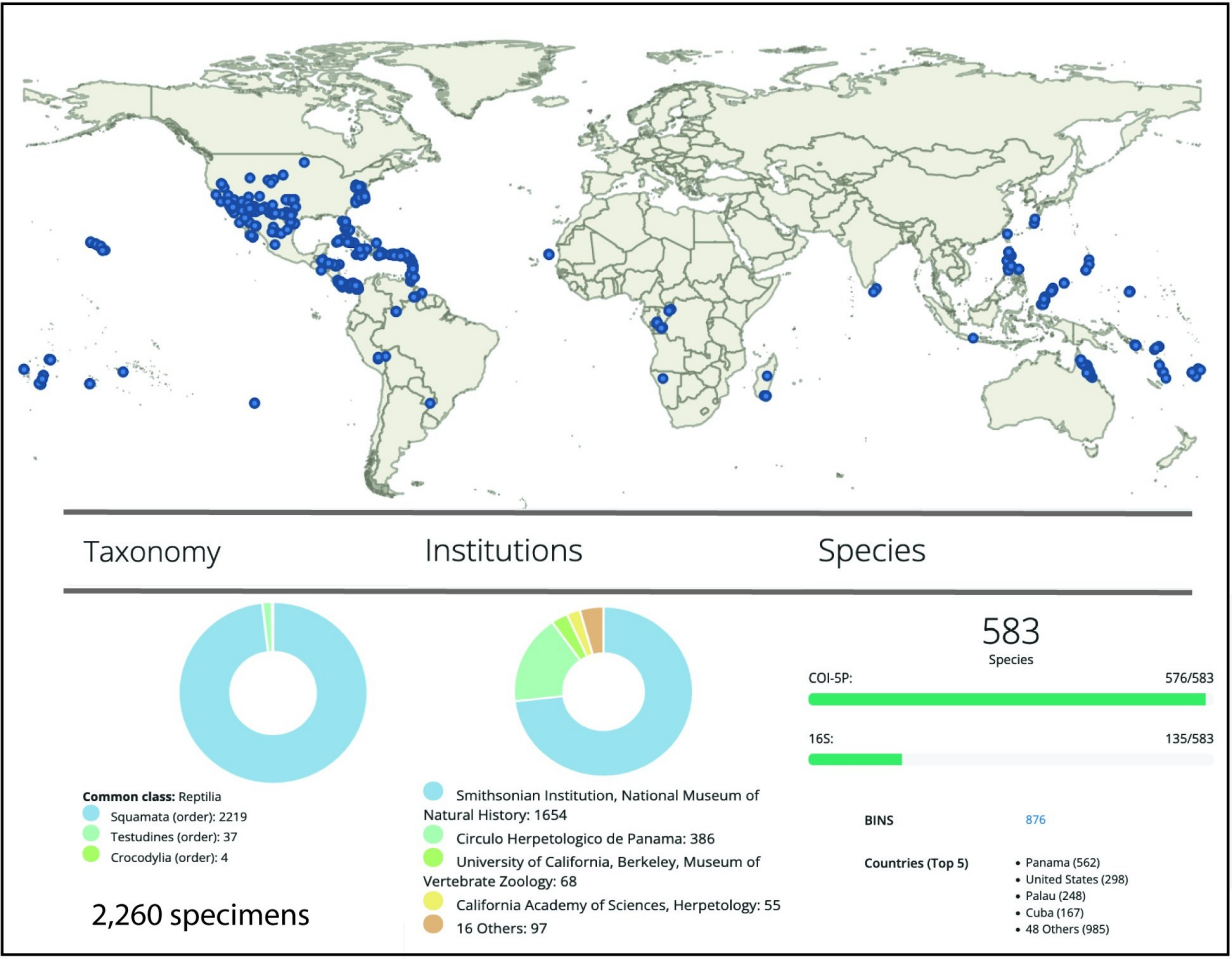
Distribution and summary data for sequence samples produced in this study. Upper: World map (1:250 million) showing geographic distribution of specimens sequenced in this study. Lower: left chart shows the taxonomic breakdown of specimens sequenced; middle chart shows institutions housing the voucher specimens; right chart shows the number of currently recognized species (for both *COI* and *16S* markers), the number of Barcode Index Numbers (BINs) represented by *COI* sequences, and the number of specimens per country, for the top five countries.

Our combined DNA barcode library (NMNHR + SQUAP) consisted of 2,205 *COI* sequences and 553 *16S* sequences and is contained in the National Museum of Natural History, Herps (NMNHH) project in BOLD. Those sequences were from 2,260 reptile specimens, consisting of 2,219 squamatans, 37 testudines, and four crocodilians. Most specimens were from Panama, followed by USA, Palau, Cuba and 48 additional countries (Fig 1). With the addition of *16S* DNA sequences for some of our samples, our project provided 2,758 new sequences to GenBank and BOLD. The *COI* sequences were placed in 876 BINs and represent 583 currently named species, 81 specimens identified only to genus, and one identified only to family. The discrepancy between the number of named species (583) and the number of BINs (876) was explained by a combination of undescribed species (e.g., some groups of anoles, geckos, and skinks), some of which were previously known and others of which were not, and widespread species that exhibit considerable genetic and geographic variation (e.g., *Laticauda colubrina* and *Pituophis catenifer*). By contrast, cases where multiple species were placed in a single BIN were found in closely related turtles (*Rhinoclemmys*) and parthenogenetic lizards (*Aspidoscelis* and *Lepidodactylus*). The ML phylogeny for our *COI* sequences is provided in S1 Fig. Below, we provide a results and discussion section for some of the taxa barcoded, with particular attention to species that were placed in multiple BINs and/or were inferred to be non-monophyletic in our phylogenetic analyses, where multiple species were placed in single BINs, and where interpretation of our results was complicated by misuses of museum data from previous studies. A more general discussion on misuse of museum data and related issues, as well as potential ways to avoid or them, follows.

## Taxon-specific results and discussion

### Testudines

We sequenced 37 turtles, including one pleurodiran (*Chelus frimbriata*), and the rest cryptodirans. Among the cryptodirans, we sequenced one sea turtle (*Dermochelys coriacea*), one snapping turtle (*Chelydra serpentina*), three geoemydid species (*Rhinoclemmys annulata*, *R*. *funerea*, and *R*. *punctularia*), several emydids (*Chrysemys picta*, *Terrapene carolina* and *T*. *ornata*, *Trachemys scripta*, and *Pseudemys rubriventris*), and kinosternids (*Kinosternon flavescens*, *K*. *leucostomum*, *K*. *scorpioides*, *K*. *subrubrum*, and *Sternotherus odoratus*). Of the *Rhinoclemmys*, we sequenced two *R*. *funerea* and one *R*. *punctularia* that were placed into a single BIN. This BIN also contains an additional *R*. *punctularia*, two *R*. *melanosterna*, and three other *R*. *funerea*, all from other BOLD projects. These species have all been shown to have conserved mtDNA (1.3–3.0% p-distance) but have been maintained as different species based on allopatric and parapatric distributions, morphology, and karyotypic differences [16]. This represents a case where closely related species can occupy a single BIN. We also sequenced three *Terrapene carolina*: one *T*. *c*. *carolina*, and two *T*. *c*. *triunguis*, which were placed in separate BINs for each subspecies. Paraphyly of *T*. *carolina* (relative to *T*. *coahuila*) has been inferred based on nuclear and mtDNA (including the *COI* barcode locus) and used to justify recognizing several lineages as species, including separation of *T*. *c*. *carolina* and *T*. *c*. *triunguis* in different species [17]. However, others have chosen to maintain *T*. *c*. *triunguis* as a subspecies of *T*. *carolina* based on known introgression [18]. Among the kinosternids, we sequenced two *K*. *scorpioides*, which were placed in separate BINs, one from Honduras (USNM 559581) and one from central Panama (CH 5691); these likely represent different subspecies [19] and may represent different species [20].

### Squamata—lizards (excluding snakes)

#### Anolis

Our project contains 548 Anolis specimens representing 123 currently recognized species. The COI barcode data ML tree did not infer Anolis to be monophyletic, a result that is contradicted by studies with more data [21]. This is likely (as noted in the Materials and Methods) because ~655 bp of COI cannot resolve over 50 million years of evolution [21]. However, the ML tree was useful for clustering groups of closely related sequences to assign specimens to species. Within Anolis, the barcode data provide interesting insights, such as the placement of samples from each of several widespread species in different BINs, suggesting that some may represent multiple species. In addition, some BIN clusters were not monophyletic in our COI tree. Some of these cases involve taxa previously known to represent multiple species (but not formally proposed). To investigate them further, we constructed an Anolis dataset in BOLD (DS-NMNHA) of 787 sequences from BOLD and GenBank (including the 548 presented herein). We included one Polychrus gutturosus from our study (CH 5693) and one P. marmoratus from GenBank (JN112789) as outgroups (S2 Fig). This resulted in 20 species that were each divided into multiple BINs (other species each formed single BINs). In 12 of those species, the BINs for each species collectively formed a clade (A. alutaceus, A. apletophallus, A. capito, A. carolinensis, A. garmani, A. humilis, A. lemurinus, A. loysiana, A. lineatopus, A. polylepis, A. pulchellus, and A. sagrei), whereas in the other eight species, the BINs for each species did not (A. allogus, A. angusticeps, A. cristatellus, A. equestris, A. limifrons, A. poecilopus, A. porcatus, and A. roquet). Some of the “species” placed in multiple BINs are known to exhibit considerable genetic variation, such as A. carolinensis [22] and A. sagrei [23], whereas others are known to be paraphyletic, such as A. porcatus relative to A. allisoni, A. smaragdinus, and A. carolinensis [24].

Several *Anolis* sequences currently appear to be mis-labelled in GenBank. For instance, KP100436 (POE 2276) is currently in GenBank as *A*. *zeus* but is treated as *A*. *polylepis* in the associated paper [25], and KP231854 (SMF 96459) is in GenBank as *A*. *quercorum* but is treated as *A*. *sacamecatensis* in the associated paper [26]. Additionally, with our *16S* sequences we discovered a study with 52 associated sequences submitted to GenBank as *A*. *tropidonotus*, of which only four were considered *A*. *tropidonotus* by the submitting authors [27]. The others 48 sequences represent two newly named species (*A*. *mccranei* and *A*. *wilsoni*) and a third resurrected species (*A*. *spilorhipis*). Submitting sequences to GenBank prior to publication under taxonomic names accepted at that time and failing to update the names once the proposal of the newly recognized species is published appears to be a common cause of outdated taxonomic information (see General Discussion).

#### Aspidoscelis

Specimens of *Aspidoscelis flagellicauda*, *A*. *sonorae*, and *A*. *uniparens* shared identical *COI* barcodes. This is not surprising because these taxa are parthenogenetic species of hybrid origin with very similar mtDNA [28, 29]. *Aspidoscelis sonorae* and *A*. *flagellicauda* are difficult (but not impossible) to distinguish morphologically, but *A*. *uniparens* is more distinctive (unspotted). According to Wright’s [28] hypothesis, all three species (*A*. *flagellicauda*, *A*. *sonorae*, and *A*. *uniparens*) are thought to have received their mt-genomes from *A*. *inornata*. Our results are consistent with that hypothesis as all three species have nearly identical *COI* sequences (mean pairwise p-distance = 0.25%), were assigned to the same BIN, and were placed sister to the single sample of *A*. *inornata*. This is a case where species cannot be distinguished by DNA barcoding.

#### Emoia

We sequenced 25 specimens of *Emoia caeruleocauda* from three distant and distinct Oceania regions: Marianas, Fiji, and Palau. Each region has a genetically distinct population, the members of which were placed in separate BINs. Zug and Ineich [30] provided morphological evidence that the Fijian population arose from an old, natural dispersal event and not a recent human introduction. The present genetic data support that conclusion and that the Fijian population, as well as the other two, have diverged genetically and may represent different species, some for which existing names may be available [30].

#### Gehyra

We sequenced 29 specimens of *Gehyra oceanica*, which were placed in four BINs corresponding to three clades and a single specimen. Two specimens (USNM 539356 and USNM 539380) from Palau (Sonsorol, Southwest Island Group) were placed in the same BIN and are sister (1.2% p-distance) to the nearest-neighbor BIN containing 11 specimens from Palau (Babeldaob, Ngeanges, Ngerechur, and Ngerur, and Ngkesill islands), the Federated States of Micronesia (Pohnpei), and the Northern Mariana Islands. The large *Gehyra* from the Southwest Island Group of Palau have long been suspected of being a distinct species [31]. These two BINs formed a clade in our *COI* tree and are sister to another group of specimens from Tonga, Cook Islands, and French Polynesia (Polynesia), and the Temotu Province of the Solomon Islands and Fiji (Melanesia). Of these Polynesian + Melanesian specimens, one (USNM 533760) from Tahiti, French Polynesia was placed in its own BIN and is sister to the remaining specimens, which were placed in single BIN (the nearest-neighbor to the BIN containing USNM 533760, which is 4.98% divergent) containing specimens from Tonga, Cook Islands, and Tahiti, French Polynesia (Polynesian) and Fiji and the Solomon Islands (Melanesian). The two Tahiti specimens (in different BINs) suggest the possibility of two species on Tahiti, although nuclear markers should be included in reassessments. The two large clades, Micronesian and Polynesian + Melanesian, are 5% different and were previously also considered to potentially be different species based on morphological and biochemical data [31]. Our two main clades correspond to the Micronesia (M1) and Polynesia + Melanesia (P1) clades of Tonione et al. [32], who used some of the same specimens and found evidence of six lineages within *G*. *oceanica*. Our specimen (USNM 533760) from Tahiti in its own BIN appears to represent an additional lineage, as it is placed sister to the M5 + P1 clade of Tonione et al. [32] in a *COI* neighbor-joining tree including their specimens (not shown). We maintain all of these specimens as “*G*. *oceanica*” but realize this taxon may represent several distinct species [32].

#### Gekko

We sequenced 10 specimens (USNM 498340, USNM 514063, USNM 546223, USNM 584620, USNM 584624, USNM 584628, USNM 584638, USNM 584642–43, and USNM 584645) previously identified as *G*. *vittatus* from Palau and two specimens of *G*. *vittatus* from the Solomon Islands. The Palauan populations of *Gekko vittatus* have long been recognized as distinct from other *G*. *vittatus* [31], and were more recently described as *Gekko remotus* [33] based on morphological data. Our barcode data support this conclusion as the Palau populations were placed in their own BIN and are ~8% divergent from specimens from the Solomon Islands.

#### Hemidactylus

**Fig 2:**
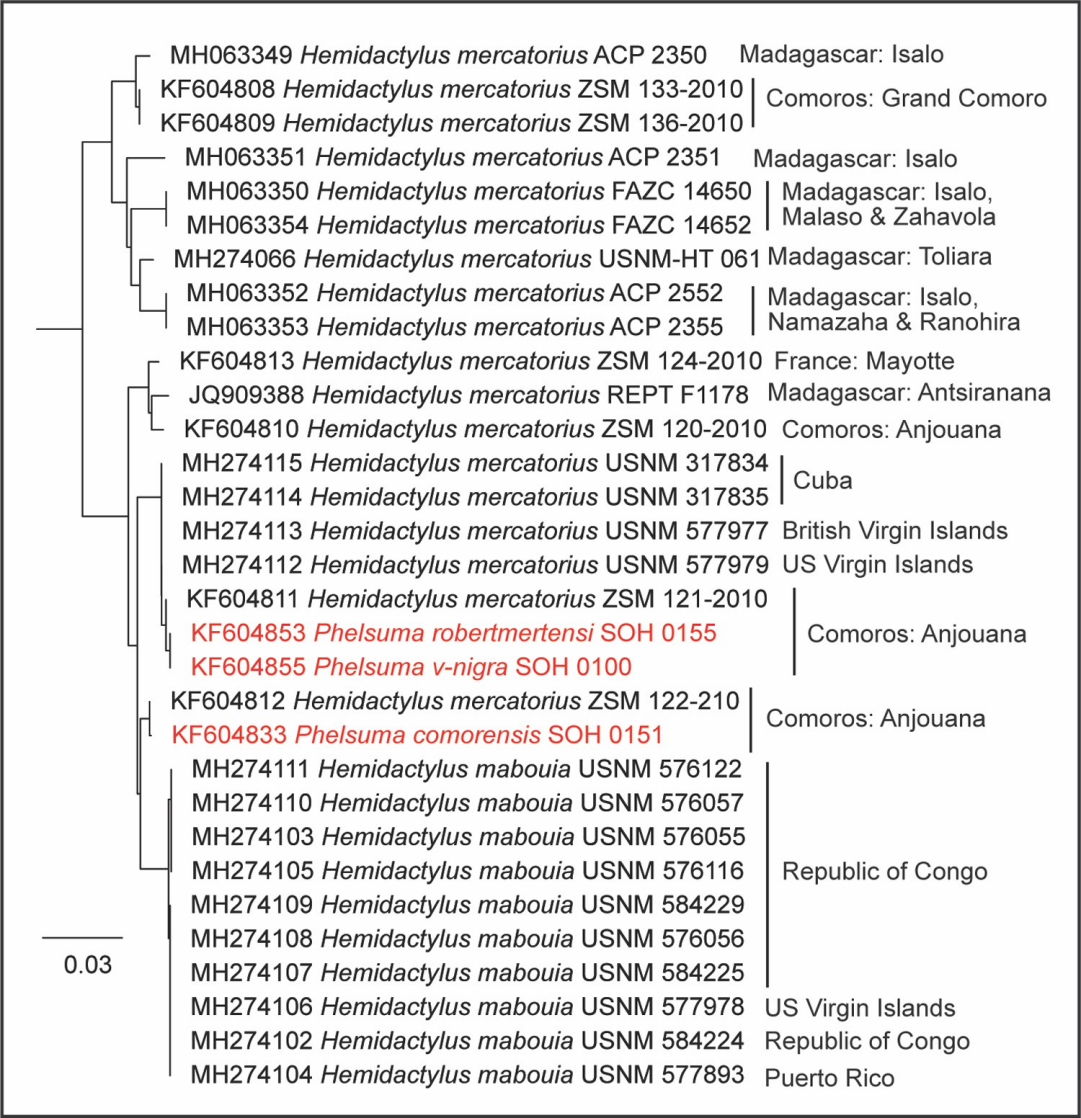
NJ tree of *Hemidactylus mabouia* and *H*. *mercatorius COI* sequences in GenBank. Sequences are shown by GenBank Accession number, followed by the species, and specimen number. Localities are shown to the right. Red sequences are mis-labelled specimens in GenBank and BOLD (see text). The localities given for the mis-labelled specimens are those of the *H*. *mercatorius* samples to which they correspond, inferred from the published tree [39]. GenBank Accession numbers beginning with MH274 are from this study, those beginning with KF604 are from Hawlitschek et al. [39].

The *H*. *mabouia*–*mercatorius* complex ranges through Madagascar and Sub-Saharan Africa to the neotropics, including northern South America and the Caribbean [34]. We sequenced 15 specimens in this complex: eight from the Republic of Congo (USNM 576055–57, USNM 576116, USNM 576122, USNM 584224–25, and USNM 584229); two from Cuba (USNM 317834–35); two from the US Virgin Islands (USNM 577978–79); one from the British Virgin Islands (USNM 577977); one from Puerto Rico (USNM 577893); and one from Madagascar (USNM-HT 061). Several researchers recognize insular specimens from the Gulf of Guinea, Comoros, Madagascar, Mayotte, and the Seychelles as *H*. *mercatorius*, with considerable genetic (*16S* mtDNA) variation within [35–37], whereas others continue to recognize all populations as *H*. *mabouia* [38]. *COI* sequences exist for *H*. *mercatorius* from a study on DNA barcoding of Comoran Reptiles [39]. Several of these sequences seem to be mis-identified. For example, two samples supposedly of *Phelsuma* (KF604853, SOH0155 and KF604855, SOH0100) were placed in the same BIN as some of our *H*. *mercatorius* samples and are nested within that species in a NJ tree (Fig 2). We downloaded all of the DNA barcodes of Hawlitschek et al. [39] from GenBank (the specimens, but not the project, are public in BOLD) and noticed that several sequences contain stop-codons; these sequences are flagged in GenBank as “Unverified” (e.g., KF604884). A NJ tree generated from these sequences (not shown) does not match the tree presented in their study; the main differences are the many presumably mis-labelled sequences, several of which are nested within distantly related taxa (e.g., *Hemidactylus* and *Phelsuma* sequences nested within *Fucifer*, and a *Trachylepis* sequence nested within, and identical to some, *Hemidactylus*). One supposed *H*. *mercatorius* sample (KF604815, SOH0065) is 99.5% similar to a skink (*Amphiglossus johannae*, KF604751), and another sample (KF604814, SOH00654) is 99.6% similar to a chameleon (*Fucifer cephalolepis*, KF604786). We urge researchers to check their alignments for stop-codons and download and generate trees with their data after it has been uploaded to BOLD to ensure that errors did not occur during the upload process.

Despite these errors, a NJ tree of our *Hemidactylus COI* sequences and others from GenBank has *H*. *mercatorius* paraphyletic with respect to *H*. *mabouia* (Fig 2). Five of our samples in the *H*. *mabouia*–*mercatorius* complex were placed among two *H*. *mercatorius* clades: the sample from Madagascar (also placed in its own *COI* BIN) was placed in a clade with other Malagasy Region specimens, and the two from Cuba, the one from the British Virgin Islands, and one (of two) from the US Virgin Islands (USNM 577979) were placed in a clade with at least one Comoran specimen (and were placed in a single BIN with other individuals from Hawlitschek et al. [39]). The other US Virgin Islands sample (USNM 577978), the one from Puerto Rico, and those from the Republic of Congo were placed in a *H*. *mabouia* clade. The global distribution of this species complex was assessed recently using the mtDNA locus *ND2* and several nuclear loci [34]. Although the taxonomic sampling of that study was dense regarding *H*. *mabouia* specimens from sub-Saharan Africa, it contained only a few samples from the Caribbean and only three *H*. *mercatorius* specimens from Madagascar. The authors suggested that the *H*. *mabouia* complex may be composed of more than 20 putative species, ranging throughout sub-Saharan Africa to the Caribbean, with *H*. *mabouia sensu stricto* (*ss*) ranging from Ghana through the Congolian and northern Zambezian regions, the South African and extreme southern Zambezian regions, and the neotropics. *Hemidactylus mabouia ss* was found to be sister to *H*. *mercatorius*, and this *H*. *mabouia ss + H*. *mercatorius* clade was nested within several clades of *H*. *mabouia sensu lato* (*sl*), which included several additional samples from the Zambezian and South African regions [34]. Our dataset includes some of the same *H*. *mabouia* specimens sequenced by Agarwal et al. [34] from the Republic of Congo, which represent *H*. *mabouia ss*, as does our sample from Puerto Rico and one (of two) from the US Virgin Islands. Our results show that both *H*. *mabouia ss* and *H*. *mercatorius* occur in the Caribbean, where the presence of *H*. *mercatorius* (in at least Cuba and both the British and US Virgin Islands) represents a novel finding. We also note that Agarwal et al. [34] did not detect *H*. *mercatorius* in the Gulf of Guinea, as reported by Rocha et al. [36]. Studies of the *H*. *mabouia–mercatorius* complex to date have used different mtDNA loci (e.g., *16S* [37], *ND2* [34], and *COI* [39] and this study), which prohibits direct comparison of their results. Until additional studies are conducted, including numerous individuals from each of these localities with the same markers sampled, the distributions of the members of the *H*. *mabouia*–*mercatorius* complex remain uncertain.

#### Holbrookia

We barcoded 29 specimens of *Holbrookia*, including all six currently recognized species, including one *H*. *approximans*, one of the recently elevated *H*. *subcaudalis* [40], and several of the other four species (*H*. *elegans*, *H*. *lacerata*, *H*. *maculata*, *and H*. *propinqua*). Eleven specimens of the widespread and polytypic *H*. *maculata* were placed into five BINs. In comparing our results with the Supporting Information of Chambers and Hebert [41], another DNA barcoding paper on amphibians and reptiles of North America (their S1 Fig Reptilian neighbor-joining tree), we noticed that they included five “*H*. *maculata*” specimens, three of which clustered with *Callisaurus*, *Cophosaurus* and *Uma*, their inferred closest relatives [42, 43], whereas the remaining two were placed within *Phrynosoma*. Therefore, we constructed a NJ tree with their samples and ours for Phrynosomatidae (not shown). This tree placed their two samples (from Durango, MX) within our *H*. *approximans-H*. *subcaudalis-H*. *lacerata* clade, sister to our *H*. *lacerata-H*. *subcaudalis* clade, with deep divergence (p-distance ≈ 10%). The other three specimens (from Mazatlán, MX, the type locality of *H*. *elegans* [44]) were sister to our *H*. *elegans* clade. Our clade represents *H*. *e*. *thermophila* as it included two specimens from Guaymas, MX (IBUNAM 28140–41), the type locality of that taxon [45]. The Mazatlán specimens were placed in a separate BIN and are > 9% different from our samples, suggesting that *H*. *e*. *elegans* and *H*. *e*. *thermophila* may be different species. In any case, none of the five specimens identified as *H*. *maculata* by Chambers and Hebert [41] is a member of the species *H*. *maculata* as currently recognized. We interpret their being labelled in BOLD and GenBank as “*H*. *maculata*” as a case where the museum identifications were based on an old taxonomy that the users failed to update when they published these sequences (see General Discussion).

#### Lamprolepis

We sequenced 34 specimens of *Lamprolepis smaragdina* from five countries (Philippines, Federated States of Micronesia, Palau, Solomon Islands, and Northern Mariana Islands), which were placed in 6 BINs. Two specimens (USNM 497566 and USNM 509418) from the Philippines were each placed in their own BIN, and several specimens from the Solomon Islands (USNM 533555, USNM 533563, USNM 533575, and USNM 533577–79) were placed in a single BIN. Specimens from the Federated States of Micronesia (USNM 576348–49, USNM 576886, and USNM 576888) and some from Palau (USNM 507549, USNM 513647, USNM 584766, USNM 584863) shared a BIN, while one from the Northern Mariana Islands (USNM 571626) and others from Palau (USNM 584767, USNM 584775, USNM 584778, USNM 584785, USNM 584790, USNM 584804–05, USNM 584807–08, USNM 584810, USNM 584824, USNM 584836–37, USNM 584839, USNM 584850, USNM 584862) shared another BIN, and a single specimen (USNM 559804) from Palau was placed in its own BIN. The taxon *Lamprolepis smaragdina* has been shown to be a complex, likely containing multiple species [46]. Based on geography alone, our specimens likely would fall into clades 4 (northern Philippines) and clade 6 (primarily Papua New Guinea and west Pacific populations, but also eastern satellite islands of Sulawesi) of Linkem et al. [46]. Those authors included some of the same USNM specimens sequenced by us, from the Solomon Islands, the Federated States of Micronesia, and Palau [46], but sequenced a different gene region (*ND2*). Although they provided an appendix with voucher information, their tree figures use only country and/or island as specimen identifiers. This makes it difficult, if not impossible, to determine exactly which specimens are in which clades, without reanalyzing their data with properly labelled specimens. If this complex is divided into several species, it is likely that the specimens barcoded herein will represent multiple species.

#### Lepidodactylus

We sequenced 98 specimens of *Lepidodactylus*: 58 *Lepidodactylus lugubris*, 18 *L*. *moestus*, four *L*. *vanuatuensis*, two *L*. *paurolepis*, two *L*. *euaensis*, one *L*. *manni*, and 13 others not identified to species. Two specimens not identified to species (USNM 533293 and USNM 533296) from the Solomon Islands were nested within *L*. *vanuatuensis* in our *COI* tree, but were placed in a separate BIN (4.5% different from the four specimens of *L*. *vanuatuensis*). Heinicke et al. [47] included one of these Solomon Island specimens (USNM 533293) in their study and found it sister to *L*. *vanuatuensis* based on mitochondrial (*ND2*) and nuclear (*RAG1* and *PDC*) data, and treated it as an undescribed species. Our *COI* data support this conclusion. Another specimen (USNM 585519) was placed sister to *L*. *paurolepis*, also in its own BIN, and 10 from Palau (including USNM 531971), all placed in their own BIN, likely represent an unnamed species [31, 47]. *Lepidodactylus lugubris* is a parthenogenetic species with six or more clones recognizable by their color patterns [48, 49]. *Lepidodactylus moestus* is thought to be the maternal parental species [50]. Our barcode data support this hypothesis as the two species are indistinguishable (0–0.8% p-distance) based on the *COI* locus, and all were placed in the same BIN.

#### Lipinia

Fourteen specimens of *L*. *noctua* (USNM 322478, USNM 322483–84, USNM 322536, USNM 322759, USNM 333531, USNM 499936, USNM 533714, USNM 533716, USNM 533720, USNM 533774–76, and USNM 571856) were placed in a single BIN, containing individuals from Fiji eastward to French Polynesia and Hawaii. This high degree of similarity across a broad geographic distribution supports an “express train” hypothesis [51], resulting from relatively recent human transport of this taxon through central Oceania. The distinctiveness of the *Lipinia* samples from Palau (USNM 512290, USNM 585287, USNM 585300, USNM 585306, USNM 585313–15, and USNM 585344–45) support the distinction of a northern Micronesian species, “*Lipinia* sp.” [31].

#### Nactus

The *COI* barcode data confirm that the Australian *N*. *eboracensis* is a distinct lineage from *N*. *cheverti* and *N*. *galgajuga*, also from Australia, and that the latter are sister species [52, 53]. The early diverging specimen of *N*. *eboracensis* (USNM 561878) was placed in a separate BIN from the other two, which is unexpected because they were collected at the same locality. The six *N*. *multicarinatus* were placed into two BINs, one composed of three specimens (USNM 333869, 333890–91) from Efate Island, Vanuatu, and the other composed of one specimen (USNM 334202) from Espiritu Island, Vanuatu, and two from the Reef Islands, Solomon Islands (USNM 533297–98). The difference between *N*. *multicarinata* samples is suggestive of differentiation within this species, which is often considered homogenous from Vanuatu to northern Papua New Guinea.

#### Plestiodon

We sequenced four *Plestiodon*, two *P*. *multivirgatus* (USNM 561174–75), one *P*. *tetragrammus* (USNM 337765), and one *P*. *laticeps* (USNM 588406). In matching these sequences with sequences in GenBank, we discovered that the *P*. *inexpectatus* (ROM 23249) from Chambers and Hebert [41], is a 97% match to a copepod (*Leptodiaptomus*), and their *P*. *laticeps* (FMNH 259981) as well as their *Urosaurus nigricaudus* (MVZ 161339 and MVZ 161341) and *Ophisaurus compressus* (FMNH 266588) sequences are 98–99% matches to the salamander species *Desmognatus monticola*. The sequences associated with these specimens are <250 bp and were neither included in their phylogenetic analyses nor “BIN’d”; therefore, the errors have escaped attention. Simple BLAST searches in GenBank prior to submitting these sequences to BOLD could have prevented these errors.

### Squamata—snakes

#### Bothrops

The *COI* data support two BINs for *B*. *asper* from Panama. The first BIN contained specimens from Darién (CH 9097), Colón (CH 5290, CH 9306), and Coclé (USNM 579859), whereas the second BIN contained specimens only from Bocas del Toro (USNM 347362, USNM 347366, CH 6625, CH 6656). In our ML tree, the two *B*. *asper* BINs formed clades and together they formed a larger monophyletic group, with *B*. *atrox* sister to it. A recent study has identified multiple clades within *Bothrops asper* [54]. They used different genes than *COI*, but it appears that our specimens extend the range of the CICA clade [54] to the Bocas del Toro region of Panama.

#### Candoia

Four individuals of *Candoia bibroni* were sequenced and are closely related, although there is genetic divergence between the single Samoa specimen (USNM 322766) from the central Pacific and three specimens (USNM 533623, USNM 534107–08) from the Reef Islands (Solomon Islands) from the southwest Pacific Rim. A study of genetic relationships among *Candoia* populations [55], found differences between Fijian and Samoan populations, which drew attention to McDowell’s [56] recognition of two morphotypes of *C*. *bibroni*, an oceanic form and a Melanesian one. Our results, although limited, show genetic differentiation of the samples from these two areas, which were placed in separate BINs. Our large sample of *C*. *superciliosa* confirms the distinctiveness and uniformity of the Palau *Candoia* population [55], which was previously considered part of *C*. *carinata* but later recognized as a separate species [57].

#### Erythrolamprus

*Erythrolamprus reginae* is paraphyletic with respect to *E*. *poecilogyrus*. The former taxon is known to be a complex likely containing multiple cryptic species [58]. Some of us (DGM and KdQ) are involved in a separate project on this group (Ascenso et al., in prep.).

#### Laticauda colubrina

We sequenced eight specimens of the widespread species *L*. *colubrina*, which were placed into three BINs, one each for populations in the Philippines (USNM 497558–59), one for Palau specimens (USNM 577702–03, USNM 513410, USNM 531997, and USNM 577712), and a single specimen from the Solomon Islands (USNM 533630).

#### Naja

We sequenced three specimens initially identified as *Naja melanoleuca* from the Department of Likouala, Republic of Congo, two from Impongui (USNM 576163 and USNM 558271) and one from near the village of Ganganya Broussen (USNM 576076). These were placed in separate BINs. A recent study found *Naja melanoleuca* to be a species complex and split it into five species [59]. That study, based on mtDNA and two nuclear loci, included all three of the same individuals we barcoded and determined the first two represent true *Naja melanoleuca*, whereas the other individual was identified as *N*. *subfulva*, previously recognized as a subspecies. The BIN results support their conclusion.

#### Oxybelis

We sequenced 22 specimens of *Oxybelis*, 12 identified as *O*. *brevirostris* and 10 originally identified as *O*. *aeneus*. Recently, *O*. *aeneus* was found to represent several species, with new species described (or resurrected) from within, ranging from the southwestern USA to northern South America, and *O*. *aeneus* was restricted to the populations from the Amazon Basin [60]. Based on *16S* data, two of our specimens (CH 5078 and CH 6080) from central and southeastern Panama (respectively) are 98–99% similar to and formed a clade with *O*. *vittatus* specimens. A third specimen (CH 5746), for which we were not able to obtain *16S* data, is in the same *COI* BIN as CH 5078 (99% identical). Therefore, we treat these three specimens as the recently resurrected *O*. *vittatus*. Based on *16S* data, another five specimens (USNM 319273, USNM 347522, USNM 347524, USNM 347527, and USNM 347529) from Isla Escudo de Veraguas, in northern Panama, are 99% identical to the newly described *O*. *koehleri* [60]. A specimen (USNM 559680) from Honduras, for which we were not able to obtain 16S data, was placed in a *COI* BIN with three other specimens in BOLD. These specimens are from El Salvador, Nicaragua, and Honduras. Based on geography, we consider these specimens as *O*. *koehleri*, but note they differ from the Panama specimens by 4.5% (*COI* p-distance). The northern specimens are within the range of *O*. *koehleri*, whereas the specimens from Isla Escudo de Veraguas, Panama, would represent a significant range extension for this species, or they may prove to be a different species. For now, we refer to these specimens as *O*. cf. *koehleri*. A fourth specimen (USNM 562697) for which we were not able to obtain *16S* data is from southern Venezuela, which, based on geography, we interpret to represent *O*. *aeneus* sensu stricto.

#### Pituophis

**Fig 3:**
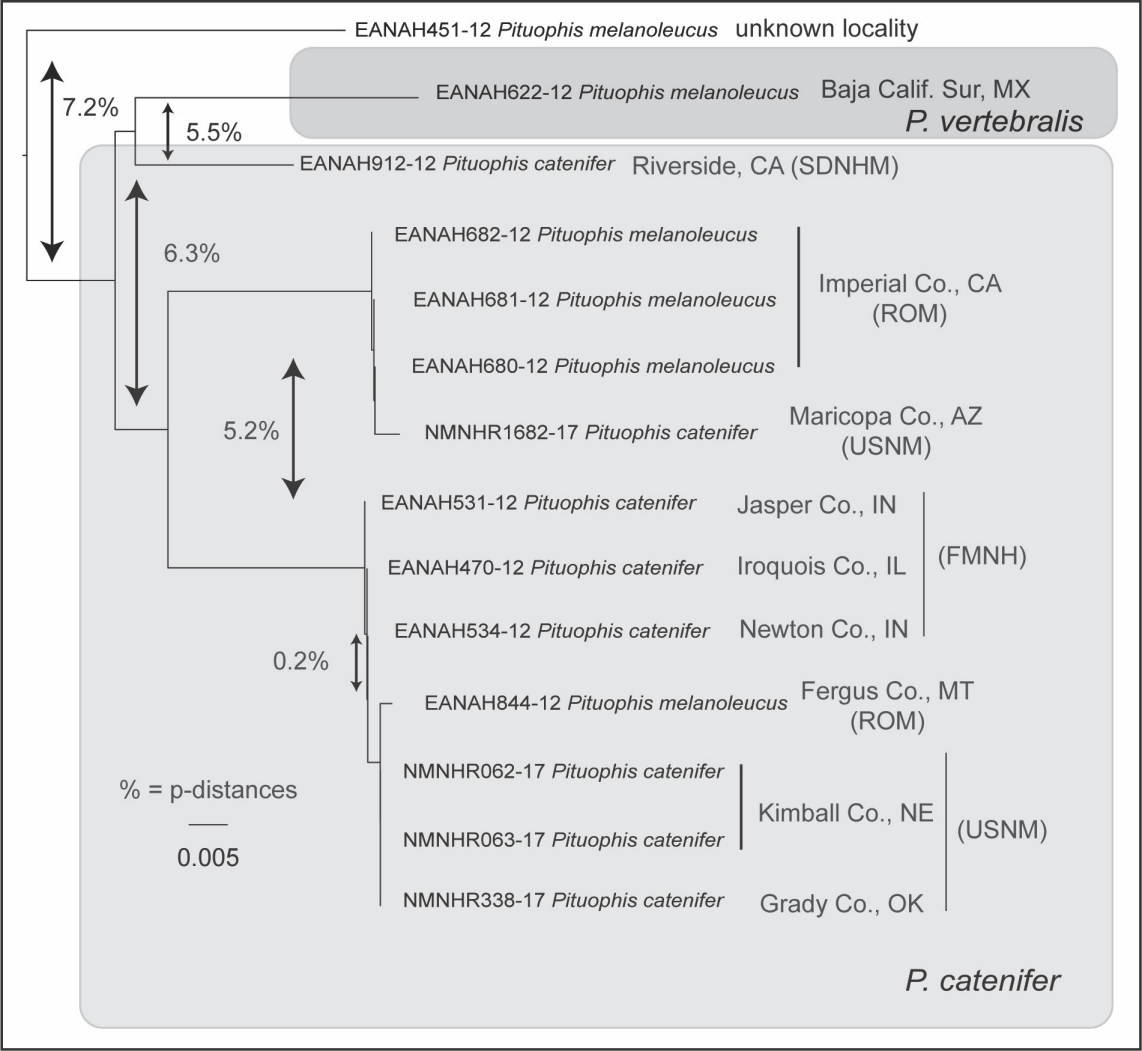
Neighbor-joining tree of *Pituophis* snakes, including samples from this study and another project in BOLD. Samples from our study begin with NMNHR, specimens from Chambers and Hebert [41] begin with EANAH and included outdated names from lending institutions. In that study, samples borrowed from the Field Museum of Natural History (FMNH) and San Diego Natural History Museum (SDNMH) had updated taxonomies that separate *P*. *catenifer* and *P*. *vertebralis* from *P*. *melanoleucus*, whereas the Royal Ontario Museum (ROM) did not. The researchers published the sequences in BOLD and GenBank using the outdated taxonomy and reported overall low levels of interspecific and high levels of intraspecific genetic divergences in their study on amphibians and reptiles, with similar mis-labelled individuals throughout [41]. While the wide-ranging *P*. *catenifer* does show high levels of intraspecific divergence (6.3%), adopting the current taxonomy (at that time) would have eliminated the erroneous detection of low levels of interspecific variation and provided more accurate estimates of intraspecific variation. Average uncorrected p-distances are shown between clades or samples indicated by arrows. Specimens are presented by BOLD Process IDs, followed by the species names adopted by the lending institutions at the time of the study, with some using the updated taxonomy and others not. Grey boxes indicate updated taxonomy. Sample EANAH451-12 presumably represents true *P*. *melanoleucus*; however, no locality information was provided for that specimen.

We sequenced four individuals of *P*. *catenifer* that were placed in two BINs, one containing two specimens (USNM 561176–77) from Kimball Co., Nebraska, and one specimen (USNM 580435) from Grady Co., Oklahoma, and another BIN containing one specimen (USNM 589596) from Maricopa Co., Arizona. Upon running an identification search in BOLD, we determined that several records of *Pituophis* in BOLD were labelled with outdated species names. Our specimen from Arizona was placed in a NJ clade with three individuals from Imperial Co., California identified in BOLD and GenBank as *P*. *melanoleucus*. Our specimens from Oklahoma and Nebraska were placed in a clade with individuals from Illinois and Indiana identified as *P*. *catenifer*, and an individual from Montana identified as *P*. *melanoleucus* (Fig 3). The first molecular systematic treatment of *Pituophis*, used a single mtDNA gene (*ND4*), was published over 20 years ago [61] and split *P*. *melanoleucus* as then circumscribed into three species: *P*. *melanoleucus* in the east, *P*. *ruthveni* in Louisiana, and *P*. *catenifer*, from Indiana and Illinois west through the Great Plains to the Pacific Coast in California. The division of *P*. *catenifer*, *P*. *melanoleucus*, and *P*. *ruthveni* was shortly thereafter adopted in a widely-used list of North American amphibian and reptile species [62]. The sequences deposited by Chambers and Hebert [41] were from several institutions, two of which use the current taxonomy (SDNHM and FMNH), whereas another has not updated its records (ROM). The failure to use current names when publishing these records in GenBank and BOLD is what lead, in part, to the result of low interspecific divergences and high intraspecific divergences reported in that study [41]. The authors compared three individuals of *P*. *catenifer* from Indiana and Illinois and one individual of *P*. *catenifer* from Montana still labelled as *P*. *melanoleucus*, causing the erroneous measure of “low interspecific divergence” (0.2%). Conversely, they compared specimens still labelled as *P*. *melanoleucus* from Baja California, Mexico (*P*. *vertebralis*; [63]), Imperial Co., California and Fergus Co., Montana (*P*. *catenifer*), and a specimen of unknown locality, which likely represents *P*. *melanoleucus* as currently circumscribed, resulting in their finding “deep intraspecific divergence” (average of 7.2%). While the wide-ranging *P*. *catenifer* does exhibit high intraspecific divergence (6.3%), occupying at least three BINs (3 clades in Fig 3), this is different from the divergence reported by Chambers and Hebert [41].

## General discussion

### Specimen identification

Most natural history collections, including the NMNH, have loan policies for borrowing and using specimens and/or tissues. These policies typically include a disclaimer about specimen identifications, data errors, and outdated taxonomies. In general, it is the responsibility of the user to verify taxonomic identifications. A common misconception is that because the specimens are from an accredited museum, their taxonomic identifications must be correct and current. However, this is not always the case, as specimens may retain initial field identifications that may not have been verified by collection staff at the time of cataloguing, because of lack of time and/or taxonomic expertise in the particular geographic region or taxonomic group. Most often, however, incorrect names are the result of taxonomic changes that have occurred subsequent to the specimens being catalogued. Furthermore, when taxonomic rearrangements involve several to many taxa represented by a large number of specimens sampled across a large geographic region (e.g., the *Sceloporus undulatus* complex [64]), the workload is overwhelming and updates often are not conducted in a timely manner. In such cases, thousands of records and hundreds of jar labels may need to be changed. Moreover, because the specimens are often stored on the shelves taxonomically and/or alphabetically, such changes often require rearranging the collection.

Recently, researchers produced a DNA barcode library for numerous North American species of amphibians and reptiles by borrowing tissues from natural history collections [41]. In that study, the taxonomic names that were listed for each specimen in the museums’ databases were used without verifying that the taxonomy was current. Because the authors used the resources provided by the Center for North American Herpetology (CNAH: http://www.cnah.org) to compile a list of species [41], one is left to assume they followed this taxonomy. Although the CNAH database is updated regularly, it is difficult to track dates of changes in its taxonomy, and there was no mention of date accessed. However, a printed version was made available in 2009 [65]; it contains several taxonomic changes mentioned herein, the consequences of which were not adequately considered in the DNA barcode study published in 2016 [41]. For example, the authors included sequences from multiple individuals of gopher snakes (*Pituophis*) from southern California, some of which had been updated to the current taxonomy (*P*. *catenifer*) and others of which had not (*P*. *melanoleucus*) (Fig 3). In another case, involving earless lizards (*Holbrookia*), all specimens were considered *H*. *maculata* [41], when in fact none was assignable to that species as circumscribed at the time and should have been treated as *H*. *lacerata*, *H*. *approximans*, and *H*. *elegans* according to the current taxonomy. Intra- and interspecific genetic comparisons were made based on data containing both types of errors (treating conspecific individuals as members of different species and treating heterospecific individuals as members of the same species), and such errors explain several seemingly anomalous cases of “low interspecific divergence and deep intraspecific divergence” in their results [41]. Indeed, those authors speculated that *H*. *maculata* “may include multiple species” [41], when simply updating the taxonomy would have revealed that their samples of this taxon were considered different species at the time.

In a third case, specimens of two species of fence lizards (*Sceloporus undulatus* and *S*. *graciosus*) were reported to have nearly identical *COI* sequences [41]. This was attributed to possible hybridization, and a paper [64] was cited as evidence of hybridization between these species. However, that paper makes no mention of *S*. *graciosus* but instead concerns hybridization between *S*. *cowlesi* and *S*. *tristichus*, both of which are members of the *Sceloporus undulatus* species complex. We are unaware of any reports of hybridization between *Sceloporus tristichus* and *S*. *graciosus*. The *S*. *undulatus* sequences reported by Chambers and Hebert [41] are from western Colorado and likely represent *S*. *tristichus* [66]. It is likely that the “*S*. *graciosus*” of Chambers and Hebert [41] were mis-identified, and were also *S*. *tristichus*. Other *S*. *graciosus* samples in their study were sister to *S*. *arenicolus*, a species closely related to *S*. *graciosus*, and the “*S*. *undulatus*” in their study (including the likely *S*. *tristichus* mis-identified as *S*. *graciosus*) were sister to *S*. *virgatus*, which is closely related to the *S*. *undulatus* complex [67].

Several other sequences from that study [41] appear to have resulted from PCR contamination: one identified in BOLD and GenBank as a skink (*Plestiodon*) is a 97% match to a copepod, and several others identified as three distantly related lizard species are 98–99% identical to sequences from one salamander species. Many of the amphibian sequences in that study were also associated with mis-identified specimens or outdated taxonomies (see Bell et al. [68]). The lesson here is that all specimen identifications should be checked before publishing, making sure that the vouchers are identified correctly and the taxonomy is up to date. Similarly, sequences should be checked for possible contamination and those that appear to be contaminated should not be published. Simple BLAST searches in GenBank can quickly identify such contamination errors and should be conducted on any questionable sequences. Although GenBank and BOLD databases have been shown to be largely accurate at the “genus” and higher levels [69], their accuracy for species identifications is considerably lower [70], and detailed studies such as that of Bell et al. [68] and this one have revealed numerous misidentified sequences. As more and more environmental DNA and forensic studies are conducted, which rely on such databases for identifications, the value of accurate, voucher-based reference material becomes increasingly important [69, 70]. All efforts to avoid contributing mis-identified sequences to the databases should be taken, including reexamination of the data after they are submitted to repositories but before they are publicly released. For example, in a DNA barcode study referred to in the *Hemidactylus* section above [39], reexamination of the data could have prevented mis-labelled specimens from being released in GenBank and BOLD. When questionable results are obtained that cannot be explained by contamination or lab errors, we recommend that researchers reach out to the collection staff of the lending institution to determine if they can be explained by identification errors or an outdated taxonomy; whenever possible, voucher specimens should be examined.

### Vouchers, catalogue numbers, and collection codes

We cannot over-emphasize the importance of voucher specimens in taxonomic research [1], including molecular studies [71], yet papers continue to be published without them (e.g., [72]). Additionally, some researchers publish papers without depositing their sequence data in public databases (e.g., [73]). In certain cases of rare and endangered species, toe- or skin-clips, or blood samples may be all that can be legally collected. When whole-specimen vouchers are not possible, digital images should be taken and deposited in museums as photo vouchers. Collection of voucher specimens (or photographs) and deposition of the data in public repositories are the responsibilities of the scientists who conduct the studies, but those practices need to be enforced by reviewers and journal editors. A contribution to the DNA barcode library for snakes provided evidence of multiple putative cryptic species in Thailand [72]. This study was based on blood samples that were taken from a snake farm in Bangkok, Thailand; neither voucher specimens nor photographs were provided, and the localities were given only as “Thailand, Bangkok.” Although this study suggests the existence of several new species, the lack of voucher specimens and detailed locality information make it problematic to integrate the sequence data into any future studies attempting to delimit those species morphologically and geographically and to name them according to accepted taxonomic procedures.

Another common mistake, particularly with the USNM specimens in GenBank, is that researchers report the catalogue number incorrectly. GenBank has a Biocollections Database, which includes a list of acceptable museum collection codes recognized by GenBank and the designated institutions [74]. Institutions should, and often do, include appropriate information in loan agreements for citing their material in third-party databases and publications. The NMNH Division of Amphibians and Reptiles issues specimen tags from two different series: field series tags (labelled “USNM-FS” or “USNM-FH”) that are assigned to specimens in the field, and collection tags (labelled “USNM”) that are assigned to specimens when they are catalogued into the collection. Researchers may be confused by this and omit the “FS”, thinking that the field number is the catalogue number. For instance, in a study of skink diversification [75] three *Caledoniscincus atropunctatus* sequences were published with the specimen vouchers given as USNM 33456–33458, when in fact USNM 33456–33458 are catalogue numbers for turtles (*Chrysemys picta*) and USNM-FS 33456–33458 are the field numbers assigned to the skinks, whose catalogue numbers are USNM 334364–334366. This problem may be compounded as many institutions are now using linear and 2D barcoded numbers on tissue and DNA samples, in addition to catalogue numbers on the voucher specimen, and these “internal” numbers are shared on aggregate databases such as GGBN. When correct collection codes are used in GenBank [74], hovering over the collection code in the “specimen voucher” field with the cursor brings up the institution, department and/or division, and a link on the specimen number takes the user to the institution’s website for the specimen (if the museum’s database is publicly searchable). GenBank is improving at enforcing use of the correct museum codes [74], and if unrecognized initials are used, an administrator will ask the submitter to provide details, such as address and website (if available) for the collection, and they will add this information to the Biocollections database. If personal field series numbers are used, GenBank will indicate that the voucher specimens are in a private collection (e.g., [76]). The use of stable identifiers (e.g., Uniform Resource Identifiers; URI’s) for collection objects may alleviate some of the problems of linking specimens and associated data, including metadata, as more and more collections become accessible online, but challenges remain as natural history collections merge and institution domains change with the rapidly advancing digital world [77, 78].

### Updating the databases

When researchers name a new species, the new species name commonly is not associated with the GenBank and BOLD sequences from the specimens upon which that species was based (see *Anolis* above). Both databases have specific procedures for handling names of newly named species. For instance, GenBank will not use the new species name until it is published. Instead, when sequences of new species are submitted to GenBank, they are given temporary names provided by the submitter. For example, the temporary name “DGM sp2017” was used until the associated manuscript was published using the name *Bronchocela burmana* for that species [79]. In cases of species splitting, researchers commonly use the species name that would have been used for the specimens prior to splitting. In both cases, researchers often fail to request the name change once the manuscript is published, and the database managers do not track the release of every relevant publication. It is the responsibility of the authors to inform GenBank regarding the complete article reference, any taxonomic changes, and permission to release the sequences. In GenBank, only the submitters of a sequence can request changes to the species identification of that sequence. In BOLD, there is a Comments feature, which allows users to add comments, such as mis-identifications, to any sequence record. A feature like this would be useful in GenBank, though such an early-developed database may be challenged in keeping up with modern demands [80]. Additionally, researchers often fail to report taxonomic changes to the museum(s) from which they borrowed specimens. Traditionally, it was standard practice for scientists to provide reprints of any articles based on museum specimens to the lending institutions and alert them about taxonomic changes regarding the specimens involved. We encourage scientists to revive and continue this practice not only because it is a requirement of the loan policy of the NMNH and many other museums, but also because it crucially improves the data for subsequent use.

## Conclusion

Scientists using tissues from museum collections for molecular (and other) studies must do so judiciously, with an awareness of museum practices and policies. When publishing data associated with museum specimens, researchers should take time to carefully evaluate species identifications, making sure they are aware of the most relevant taxonomic proposals. By depositing molecular sequences associated with these specimens in publicly available databases (e.g., GenBank) under incorrect or outdated taxonomic names, researchers compromise the data and increase the workload for anyone wanting to use those data—assuming they are aware of potential errors. Misidentified specimens can lead to profound mistakes in taxonomy [81] and result in under- and over-estimation of genetic diversity between and within species [41]. If these errors are not corrected, subsequent users may perpetuate and even compound them by drawing erroneous conclusions. We encourage scientists submitting molecular sequence data to GenBank and BOLD, as well as any other data associated with collection specimens to appropriate databases [82], to confirm species identifications and use current taxonomies, even if the museums’ databases lag behind. Any re-identifications should also be submitted to the museums. Responsible scientists should follow up when their studies are published, informing museums of publications based on their specimens, indicating any taxonomic changes, and updating all databases to which they have access. Adopting these practices will improve the quality of the data and thus benefit the entire user communities of both molecular sequence and museum databases.

## Supporting information

S1 Fig Maximum-likelihood COI phylogeny for 2205 taxa. (PDF)

S2 Fig NMNHA dataset COIBOLD NJ tree. (PDF)

## References

[1] Rocha LA, Aleixo A, Allen G, Almeda F, Baldwin CC, Barclay MV, et al. Specimen collection: an essential tool. Science. 2014; 344, 814–815. Epub 2014/05/24. doi: 10.1126/science.344.6186.814 . 24855245

[2] Kaplan S. A university is eliminating its science collection—to expand a running track. Washington Post. 2017; 5 July.

[3] Kury AB, Giupponi AP, Mendes AC. Immolation of Museu Nacional, Rio de Janeiro—unforgettable fire and irreplaceable loss. Journal of Arachnology. 2018; 46, 556–558. doi: 10.1636/JoA-S-18-094.1

[4] Padial JM, Miralles A, De la Riva I, Vences M. The integrative future of taxonomy. Frontiers in Zoology. 2010; 7, 16. Epub 2010/05/27. doi: 10.1186/1742-9994-7-16 . 20500846 PMC2890416

[5] Hykin SM, Bi K, McGuire JA. Fixing Formalin: A method to recover genomic-scale DNA sequence data from formalin-fixed museum specimens using high-throughput sequencing. PLoS One. 2015; 10, e0141579. doi: 10.1371/journal.pone.0141579 . 26505622 PMC4623518

[6] Huxley R, Quaisser C, Butler CR, Dekker RWRJ. Managing natural science collections: a guide to strategy, planning and resourcing. Abingdon, Oxon; New York, NY: Routledge; 2020.

[7] Hebert PD, Cywinska A, Ball SL, deWaard JR. Biological identifications through DNA barcodes. Proc Biol Sci. 2003; 270, 313–21. doi: 10.1098/rspb.2002.2218 . 12614582 PMC1691236

[8] Yochelson EL, Jarrett M. The National Museum of Natural History: 75 years in the Natural History Building: Smithsonian Institution Press; 1985.

[9] Sabaj MH. Codes for natural history collections in ichthyology and herpetology. Copeia. 2020; 108, 593–669. doi: 10.1643/asihcodons2020

[10] Weigt LA, Driskell AC, Baldwin CC, Ormos A. DNA barcoding fishes. Methods Mol Biol. 2012; 858, 109–126. doi: 10.1007/978-1-61779-591-6_6 . 22684954

[11] Uetz P, Freed P, Hošek J. The Reptile Database 2021 [cited April, 2021]. Available from: http://reptile-database.org/.

[12] Ratnasingham S, Hebert PD. A DNA-based registry for all animal species: the barcode index number (BIN) system. PLoS One. 2013; 8, e66213. doi: 10.1371/journal.pone.0066213 . 23861743 PMC3704603

[13] Deichmann JL, Mulcahy DG, Vanthomme H, Tobi E, Wynn AH, Zimkus BM, et al. How many species and under what names? Using DNA barcoding and GenBank data for west Central African amphibian conservation. PLoS One. 2017; 12, e0187283. doi: 10.1371/journal.pone.0187283 . 29131846 PMC5683629

[14] Mulcahy DG, Lee JL, Miller AH, Chand M, Thura MK, Zug GR. Filling the BINs of life: report of an amphibian and reptile survey of the Tanintharyi (Tenasserim) Region of Myanmar, with DNA barcode data. Zookeys. 2018; 757, 85–152. doi: 10.3897/zookeys.757.24453 . 29780268 PMC5958176

[15] Stamatakis A. RAxML version 8: a tool for phylogenetic analysis and post-analysis of large phylogenies. Bioinformatics. 2014; 30, 1312–1313. doi: 10.1093/bioinformatics/btu033 . 24451623 PMC3998144

[16] Vargas-Ramirez M, Carr JL, Fritz U. Complex phylogeography in *Rhinoclemmys melanosterna*: conflicting mitochondrial and nuclear evidence suggests past hybridization (Testudines: Geoemydidae). Zootaxa. 2013; 3670, 238–254. doi: 10.11646/zootaxa.3670.2.8 . 26438937

[17] Martin BT, Bernstein NP, Birkhead RD, Koukl JF, Mussmann SM, Placyk JS Jr. Sequence-based molecular phylogenetics and phylogeography of the American Box Turtles (*Terrapene* spp.) with support from DNA barcoding. Mol Phylogenet Evol. 2013; 68, 119–134. doi: 10.1016/j.ympev.2013.03.006 . 23523575

[18] Fritz U, Havas P. On the relcassification of Box Turtles (*Terrapene*): a response to Marin et al. (2014). Zootaxa. 2014; 3835, 295–298. doi: 10.11646/zootaxa.3835.2.10 25081452

[19] Ernst CH, Barbour RW. Turtles of the World: Smithsonian Press; 1989.

[20] Spinks PQ, Thomson RC, Gidis M, Bradley Shaffer H. Multilocus phylogeny of the New-World mud turtles (Kinosternidae) supports the traditional classification of the group. Mol Phylogenet Evol. 2014; 76, 254–260. doi: 10.1016/j.ympev.2014.03.025 . 24704303

[21] Poe S, Nieto-Montes de Oca A, Torres-Carvajal O, de Queiroz K, Velasco JA, Truett B, et al. A phylogenetic, biogeographic, and taxonomic study of all extant species of *Anolis* (Squamata; Iguanidae). Syst Biol. 2017; 66, 663–697. doi: 10.1093/sysbio/syx029 . 28334227

[22] Campbell-Staton SC, Goodman RM, Backstrom N, Edwards SV, Losos JB, Kolbe JJ. Out of Florida: mtDNA reveals patterns of migration and Pleistocene range expansion of the Green Anole lizard (*Anolis carolinensis*). Ecol Evol. 2012; 2, 2274–2284. doi: 10.1002/ece3.324 . 23139885 PMC3488677

[23] Reynolds RG, Kolbe JJ, Glor RE, Lopez-Darias M, Gomez Pourroy CV, Harrison AS, et al. Phylogeographic and phenotypic outcomes of brown anole colonization across the Caribbean provide insight into the beginning stages of an adaptive radiation. J Evol Biol. 2019; 33, 468–494. doi: 10.1111/jeb.13581 . 31872929

[24] Glor RE, Losos JB, Larson A. Out of Cuba: overwater dispersal and speciation among lizards in the *Anolis carolinensis* subgroup Molec Ecol. 2005; 14, 2419–2432. doi: 10.1111/j.1365-294X.2005.02550.x 15969724

[25] Köhler JJ, Poe S, Ryan MJ, Kohler G. *Anolis marsupialis* Taylor 1956, a valid species from southern Pacific Costa Rica (Reptilia, Squamata, Dactyloidae). Zootaxa. 2015; 3915, 111–122. doi: 10.11646/zootaxa.3915.1.5 . 25662113

[26] Köhler G, Perez RG, Petersen CB, De La Cruz FR. A revision of the Mexican *Anolis* (Reptilia, Squamata, Dactyloidae) from the Pacific versant west of the Isthmus de Tehuantepec in the states of Oaxaca, Guerrero, and Puebla, with the description of six new species. Zootaxa. 2014; 3862, 1–210. doi: 10.11646/zootaxa.3862.1.1 . 25283534

[27] Köhler G, Townsend JH, Petersen GP. A taxonomic revision of the *Norops tropidonotus* complex (Squamata, Dactyloidae), with the resurrection of *N*. *spilorhipis* (Álvarez del Toro and Smith, 1956) and the description of two new species. Mesoamerican Herpetology. 2016; 3, 8–41.

[28] Wright JW. Evolution of the lizards of the genus *Cnemidophorus*. In: Wright JW, Vitt LJ, editors. Biology of whiptail lizards (genus *Cnemidophorus*). Oklahoma Museum of Natural History 1993. Pp. 27–81.

[29] Reeder TW, Cole CJ, Dessauer HC. Phylogenetic relationships of Whiptail Lizards of the genus *Cnemidophorus* (Squamata: Teiidae): a test of monophyly, reevaluation of karyotypic evolution, and review of hybrid origins. American Museum Novitates. 2002; 3365, 1–61.

[30] Zug GR, Ineich I. Striped skinks in Oceania: The status of *Emoia caeruleocauda* in Fiji. Pacific Science. 1997; 51, 183–188.

[31] Crombie RI, Pregill GK. A checklist of the herpetofauna of the Palau Islands (Republic of Belau), Oceania. Herpetological Monographs. 1999; 13, 29–80.

[32] Tonione MA, Fisher RN, Zhu C, Moritz C. Deep divergence and structure in the Tropical Oceanic Pacific: a multilocus phylogeography of a widespread gekkonid lizard (Squamata: Gekkonidae: *Gehyra oceanica*). J Biogeo. 2016; 43, 268–278. doi: 10.1111/jbi.12645

[33] Rösler H, Ineich I, Wilms TM, Böhme W. Studies on the taxonomy of the *Gekko vittatus* Houttuyn, 1782 complex (Squamata: Gekkonidae) I. On the variability of *G*. *vittatus* Houttuyn, 1782 sensu lato, with the description of a new species from Palau Islands, Micronesia. Bonn Zoological Bulletin. 2012; 61, 241–254.

[34] Agarwal I, Ceriaco LMP, Metallinou M, Jackman TR, Bauer AM. How the African house gecko (*Hemidactylus mabouia*) conquered the world. R Soc Open Sci. 2021; 8, 210749. Epub 2021/08/14. doi: 10.1098/rsos.210749 . 34386263 PMC8334833

[35] Vences M, Wanke S, Vieites DR, Branch WR, Glaw F, Meyer A. Natural colonization or introduction? Phylogeographical relationships and morphological differentiation of house geckos (*Hemidactylus*) from Madagascar. Biological Journal of the Linnean Society. 2004; 83, 115–130. doi: 10.1111/j.1095-8312.2004.00370.x WOS:000224032600008.

[36] Rocha S, Carretero MA, Harris DJ. Diversity and phylogenetic relationships of *Hemidactylus* geckos from the Comoro islands. Mol Phylogenet Evol. 2005; 35, 292–299. doi: 10.1016/j.ympev.2004.11.023 . 15737599

[37] Rocha S, Carretero MA, Harris DJ. On the diversity, colonization patterns and status of *Hemidactylus* spp. (Reptilia: Gekkonidae) from the Western Indian Ocean islands. Herpetological Journal. 2010; 20, 83–89. WOS:000281466800004.

[38] Carranza S, Arnold EN. Systematics, biogeography, and evolution of *Hemidactylus* geckos (Reptilia: Gekkonidae) elucidated using mitochondrial DNA sequences. Mol Phylogenet Evol. 2006; 38, 531–545. Epub 2005/09/13. doi: 10.1016/j.ympev.2005.07.012 . 16154768

[39] Hawlitschek O, Nagy ZT, Berger J, Glaw F. Reliable DNA barcoding performance proved for species and island populations of comoran squamate reptiles. PLoS One. 2013; 8, e73368. Epub 2013/09/27. doi: 10.1371/journal.pone.0073368 . 24069192 PMC3772021

[40] Hibbitts TJ, Ryberg WA, Harvey JA, Voelker G, Lawing AM, Adams CS, et al. Phylogenetic structure of *Holbrookia lacerata* (Cope 1880) (Squamata: Phrynosomatidae): one species or two? Zootaxa. 2019; 4619, 139–154. doi: 10.11646/zootaxa.4619.1.6 . 31716318

[41] Chambers EA, Hebert PD. Assessing DNA barcodes for species identification in North American reptiles and amphibians in natural history collections. PLoS One. 2016; 11, e0154363. doi: 10.1371/journal.pone.0154363 . 27116180 PMC4846166

[42] Wilgenbusch J, de Queiroz K. Phylogenetic relationships among the phrynosomatid Sand Lizards inferred from mitochondrial DNA sequences generated by heterogeneous evolutionary processes. Syst Biol. 2000; 49, 592–612. doi: 10.1080/10635159950127411 12116429

[43] Schulte JA II, de Queiroz K. Phylogenetic relationships and heterogeneous evolutionary processes among phrynosomatine sand lizards (Squamata, Iguanidae) revisited. Mol Phylogenet and Evol. 2008; 47, 700–716. doi: 10.1016/j.ympev.2008.01.010 18362078

[44] Bocourt F. Études sur les reptiles. Part 3, Sect. 1, Book. 2–15, pp. 33–860, Mission Scientifique au Mexique et dans l’Amérique Centrale. Paris, Imprimerie Nationale. 1874.

[45] Barbour T. A new lizard from Guaymas, Mexico. Proceedings of the New England Zoological Club. 1921; 7, 79–80.

[46] Linkem CW, Brown RM, Siler CD, Evans BJ, Austin CC, Iskandar DT, et al. Stochastic faunal exchanges drive diversification in widespread Wallacean and Pacific Island lizards (Squamata: Scincidae: *Lamprolepis smaragdina*). J Biogeo. 2013; 40, 507–520. doi: 10.1111/jbi.12022

[47] Heinicke MP, Greenbaum E, Jackman TR, Bauer AM. Evolution of gliding in Southeast Asian geckos and other vertebrates is temporally congruent with dipterocarp forest development. Biol Lett. 2012; 8, 994–997. doi: 10.1098/rsbl.2012.0648 . 22977067 PMC3497132

[48] Moritz C, Case TJ, Bolger DT, Donnellan S. Genetic diversity and the history of the Pacific Island House Geckos (*Hemidactylus* and *Lepidodactylus*). Biol J Linn Soc. 1993; 48, 113–133.

[49] Boissinot S, Ineich I, Thaler L, Guillaume C-P. Hybrid origin and clonal diversity in the parthenogenetic gecko, *Lepidodactylus lugubris* in French Polynesia. J Herp. 1997; 31, 295–298. https://www.jstor.org/stable/1565401.

[50] Radtkey RR, Donnellan SC, Fisher RN, Moritz C, Hanley KA, Case TJ. When species collide: the origin and spread of an asexual species of gecko. P Roy Soc B. 1995; 259, 145–52.

[51] Austin CC. Lizards took express train to Polynesia. Nature. 1999; 397, 113–114. doi: 10.1038/16365

[52] Zug GR, Fisher RN. A preliminary assessment of the *Nactus pelagicus* species group (Squamata: Gekkonidae) in New Guinea and a new species from the Admiralty Islands. Zootaxa. 2012; 3257, 22–37.

[53] Heinicke MP, Greenbaum E, Jackman TR, Bauer AM. Molecular phylogenetics of Pacific *Nactus* (Squamata: Gekkota: Gekkonidae) and the diphyly of Australian species. P Calif Acad Sci, Series 4. 2010; 61, 633–646.

[54] Saldarriaga-Cordoba M, Parkinson CL, Daza JM, Wüster W, Sasa M. Phylogeography of the Central American Lancehead *Bothrops asper* (Serpentes: Viperidae). PLoS One. 2017; 12, e0187969. doi: 10.1371/journal.pone.0187969 . 29176806 PMC5703453

[55] Austin CC. Molecular phylogeny and historical biogeography of Pacific Island Boas (*Candoia*). Copeia. 2000; 2000, 341–352.

[56] McDowell SB. A catalogue of snakes of New Guinea and the Solomons, with special reference to those in the Bernice P. Bishop Museum, part III. Boinae and Acrochordoidea. J Herp. 1997; 13, 1–92.

[57] Smith HM, Chiszar D, Tepedelen K, van Breukelen F. A revision of bevel-nosed boas (*Candoia carinata* complex) (Reptilia: Serpentes). Hamadryad. 2001; 26, 283–315.

[58] Ascenso AC, Costa JCL, Prudente ALC. Taxonomic revision of the *Erythrolamprus reginae* species group, with description of a new species from Guiana Shield (Serpentes: Xenodontinae). Zootaxa. 2019; 4586, 65–97. doi: 10.11646/zootaxa.4586.1.3 . 31716142

[59] Wüster W, Chirio L, Trape JF, Ineich I, Jackson K, Greenbaum E, et al. Integration of nuclear and mitochondrial gene sequences and morphology reveals unexpected diversity in the Forest Cobra (*Naja melanoleuca*) species complex in Central and West Africa (Serpentes: Elapidae). Zootaxa. 2018; 4455, 68–98. doi: 10.11646/zootaxa.4455.1.3 . 30314221

[60] Jadin RC, Blair C, Orlofske SA, Jowers MJ, Rivas GA, Vitt LJ, et al. Not withering on the evolutionary vine: systematic revision of the Brown Vine Snake (Reptilia: Squamata: *Oxybelis*) from its northern distribution. Organisms Diversity & Evolution. 2020; 20, 723–746. doi: 10.1007/s13127-020-00461-0

[61] Rodríguez-Robles JA, De Jesus-Escobar JM. Molecular systematics of New World Gopher, Bull, and Pinesnakes (*Pituophis*: Colubridae), a transcontinental species complex. Mol Phylogenet Evol. 2000; 14, 35–50. doi: 10.1006/mpev.1999.0698 10631041

[62] Crother BI, Boundy J, Campbell JA, de Queiroz K, Frost D, Green DM, et al. Scientific and standard English names of amphibians and reptiles of North America north of Mexico: Update. Herp Rev. 2003; 34, 196–203.

[63] Grismer JL. Comments on the taxonomy of Gopher Snakes from Baja California, Mexico: a reply to Rodríguez-Robles and de Jesús-Escobar. Herp Rev. 2001; 32, 81–83.

[64] Leaché AD, Cole CJ. Hybridization between multiple fence lizard lineages in an ecotone: locally discordant variation in mitochondrial DNA, chromosomes, and morphology. Mol Ecol. 2007; 16, 1035–1054. doi: 10.1111/j.1365-294X.2006.03194.x . 17305859

[65] Collins JP, Taggart TW. Standard common and current scientific names for North American amphibians, turtles, reptiles and crocodilians, Sixth Edition. Lawrence, Kansas: The Center for North American Herpetology; 2009; 1–44.

[66] Leaché AD, Reeder TW. Molecular Systematics of the Eastern Fence Lizard (*Sceloporus undulatus*): A comparison of parsimony, likelihood, and Bayesian approaches. Syst Biol. 2002; 51, 44–68. doi: 10.1080/106351502753475871 11943092

[67] Wiens JJ, Kuczynski CA, Arif S, Reeder TW. Phylogenetic relationships of phrynosomatid lizards based on nuclear and mitochondrial data, and a revised phylogeny for *Sceloporus*. Mol Phylogenet Evol. 2010; 54, 150–160. doi: 10.1016/j.ympev.2009.09.008 19751839

[68] Bell RC, Mulcahy DG, Gotte SW, Maley AJ, Mendoza C, Steffensen G, et al. The type locality project: collecting genomic-quality, topotypic vouchers and training the next generation of specimen-based researchers. Systematics and Biodiversity. 2020; 18, 557–572. doi: 10.1080/14772000.2020.1769224

[69] Leray M, Knowlton N, Ho SL, Nguyen BN, Machida RJ. GenBank is a reliable resource for 21st century biodiversity research. Proc Natl Acad Sci U S A. 2019;116(45):22651–6. Epub 2019/10/23. doi: 10.1073/pnas.1911714116 . 31636175 PMC6842603

[70] Meiklejohn KA, Damaso N, Robertson JM. Assessment of BOLD and GenBank—Their accuracy and reliability for the identification of biological materials. PLoS One. 2019;14(6):e0217084. Epub 2019/06/20. doi: 10.1371/journal.pone.0217084 . 31216285 PMC6584008

[71] Pleijel F, Jondelius U, Norlinder E, Nygren A, Oxelman B, Schander C, et al. Phylogenies without roots? A plea for the use of vouchers in molecular phylogenetic studies. Mol Phylogenet Evol. 2008; 48, 369–71. doi: 10.1016/j.ympev.2008.03.024 . 18424089

[72] Laopichienpong N, Muangmai N, Supikamolseni A, Twilprawat P, Chanhome L, Suntrarachun S, et al. Assessment of snake DNA barcodes based on mitochondrial *COI* and *Cytb* genes revealed multiple putative cryptic species in Thailand. Gene. 2016; 594, 238–247. doi: 10.1016/j.gene.2016.09.017 . 27632899

[73] Xie Y, Wang P, Zhong GH, Zhu F, Liu Q, Che J, et al. Molecular phylogeny found the distribution of *Bungarus candidus* in China (Squamata: Elapidae). Zool System. 2018; 43, 109–117. doi: 10.11865/zs.201810

[74] Sharma S, Ciufo S, Starchenko E, Darji D, Chlumsky L, Karsch-Mizrachi I, et al. The NCBI BioCollections Database. Database. 2018; 2018. doi: 10.1093/database/bay006 . 29688360 PMC5824777

[75] Sadlier RA, Bauer AM, Wood PL Jr., Smith SA, Jackman TR. A new species of lizard in the genus *Caledoniscincus* (Reptilia: Scincidae) from southern New Caledonia and a review of *Caledoniscincus atropunctatus* (Roux). Zootaxa. 2013; 3694, 501–524. doi: 10.11646/zootaxa.3694.6.1 . 26312308

[76] Myers EA, Burgoon JL, Ray JM, Martínez-Gómez JE, Matías-Ferrer N, Mulcahy DG, et al. Coalescent species tree inference of *Coluber* and *Masticophis*. Copeia. 2017; 105, 642–650. doi: 10.1643/CH-16-552

[77] Zug GR, Mulcahy DG, Vindum JV. Resurrection of *Bronchocela burmana* Blanford, 1878 for the Green Crested Lizard (Squamata, Agamidae) of southern Myanmar. Zookeys. 2017; 657, 141–156. doi: 10.3897/zookeys.657.11600 . 28331413 PMC5345374

[78] Güntsch A, Hyam R, Hagedorn G, Chagnoux S, Röpert D, Casino A, et al. Actionable, long-term stable and semantic web compatible identifiers for access to biological collection objects. Database. 2017; doi: 10.1093/database/bax003 28365724 PMC5467547

[79] Hedrick BP, Heberling JM, Meineke EK, Turner KG, Grassa CJ, Park DS, et al. Digitization and the future of natural history collections. BioScience. 2020; 70, 243–251. doi: 10.1093/biosci/biz163

[80] Nadim T. Data labours: how the sequence databases GenBank and EMBL-Bank make data. Science as Culture. 2016; 25, 496–519. doi: 10.1080/09505431.2016.1189894

[81] Kitchener AC, Machado FA, Hayssen V, Moehlman PD, Viranta S, Esselstyn J. Consequences of the misidentification of museum specimens: the taxonomic status of *Canis lupaster soudanicus*. J Mamm. 2020; 101, 1148–1150. doi: 10.1093/jmammal/gyaa080

[82] Miralles A, Bruy T, Wolcott K, Scherz MD, Begerow D, Beszteri B, et al. Repositories for taxonomic data: where we are and what is missing. Syst. Biol. 2020; 69, 1231–1253. doi: 10.1093/sysbio/syaa026 32298457 PMC7584136

