# Supplementary material for: DNA barcoding of the National Museum of Natural History reptile tissue holdings raises concerns about the use of natural history collections and the responsibilities of scientists in the molecular age
Source: PLoS One. 2022 Mar 4;17(3):e0264930. doi: 10.1371/journal.pone.0264930 (PMC8896674; doi:10.1371/journal.pone.0264930)
Supplement: S1 Fig — (PDF) [file pone.0264930.s001.pdf]

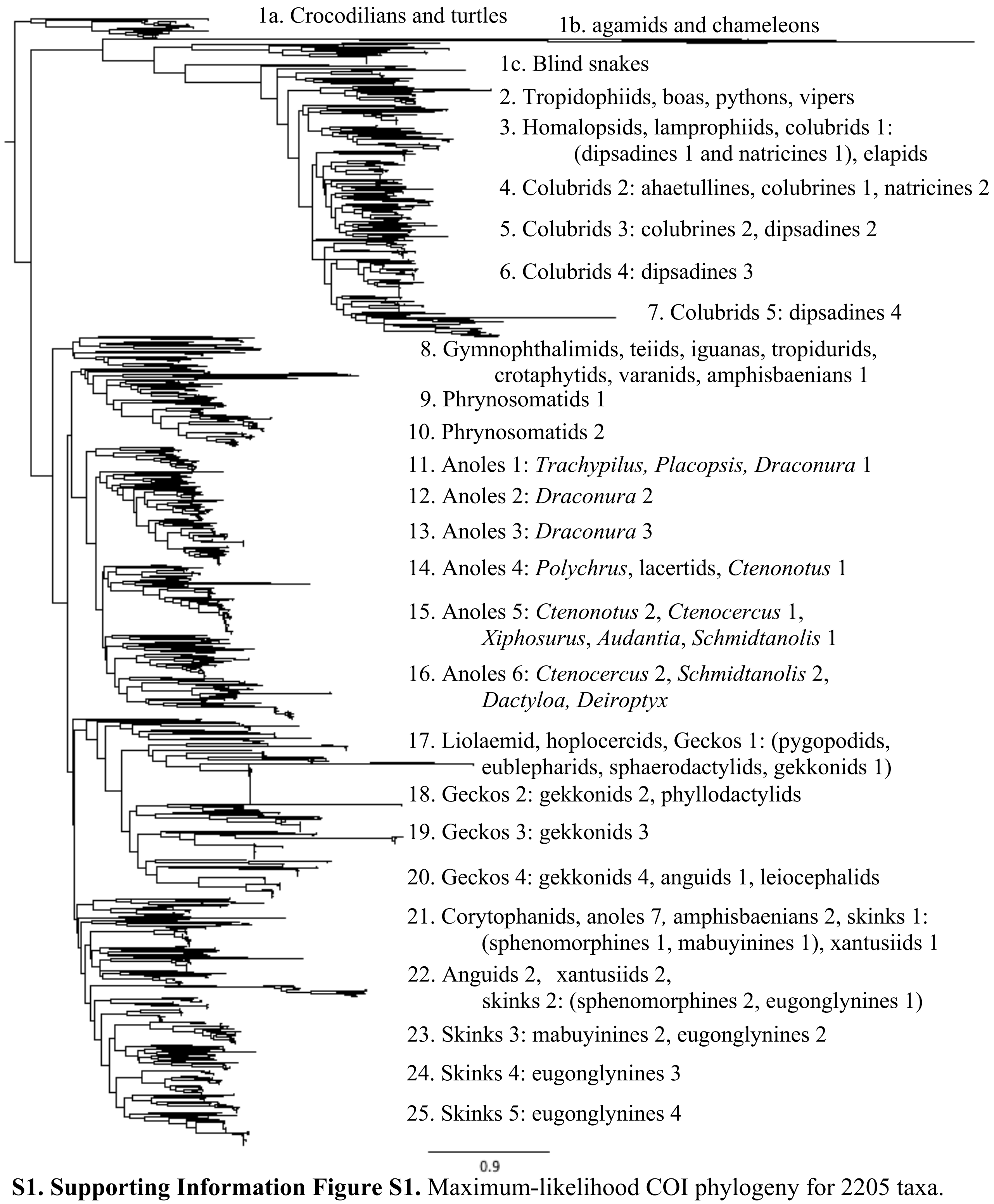

S1. Supporting Information Figure S1. Maximum-likelihood COI phylogeny for 2205 taxa. An overview of the tree is shown on this page. The groups listed are contained in the following pages 1–25. Several presumed monophyletic groups were not recovered as monophyletic because 655 bp of COI is insufficient to resolve an accurate phylogenetic estimation for such a large and ancient group.

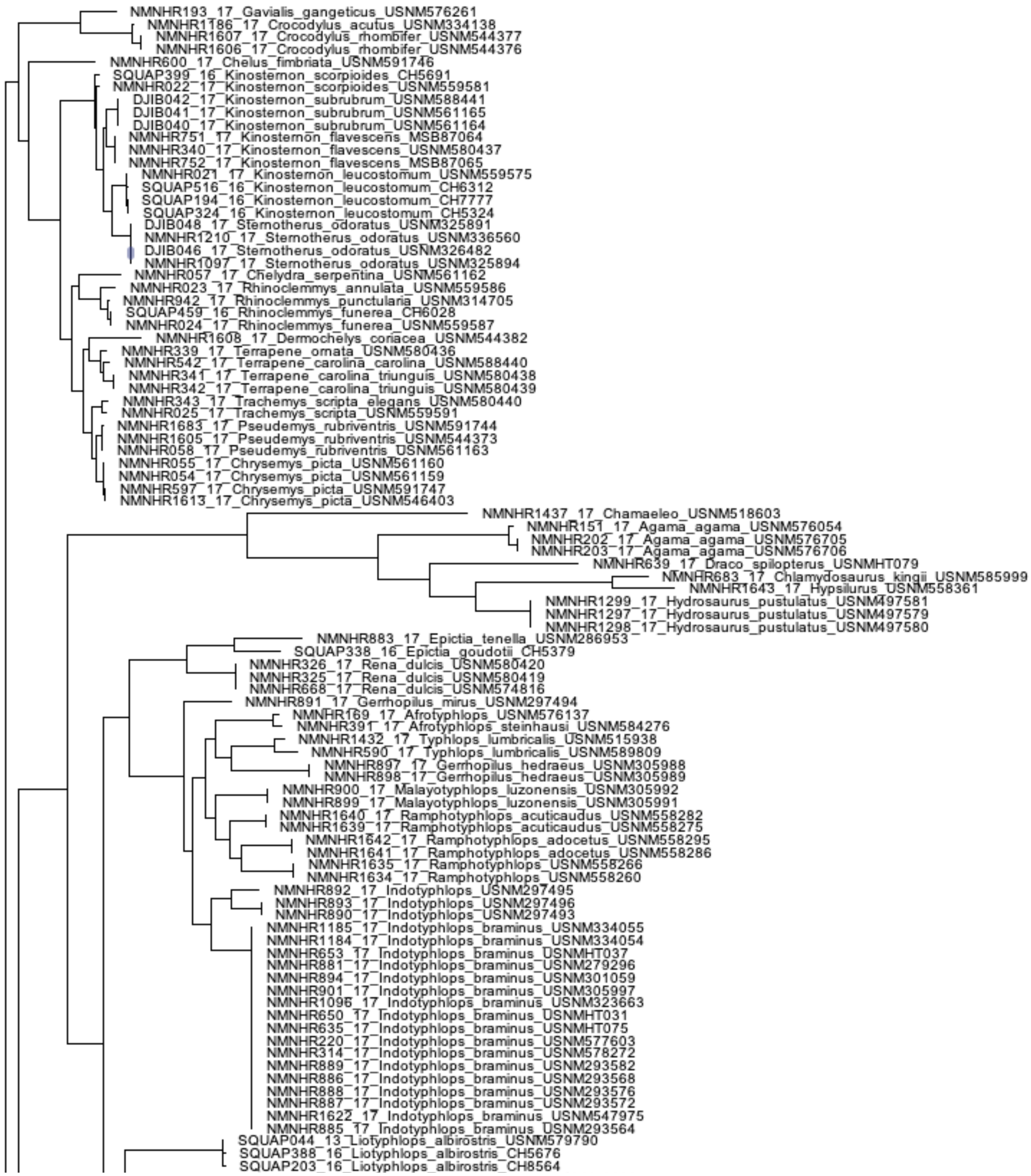

SM Figure 1.1 a–c. Crocodiles, turtles, agamids, and blind snakes.

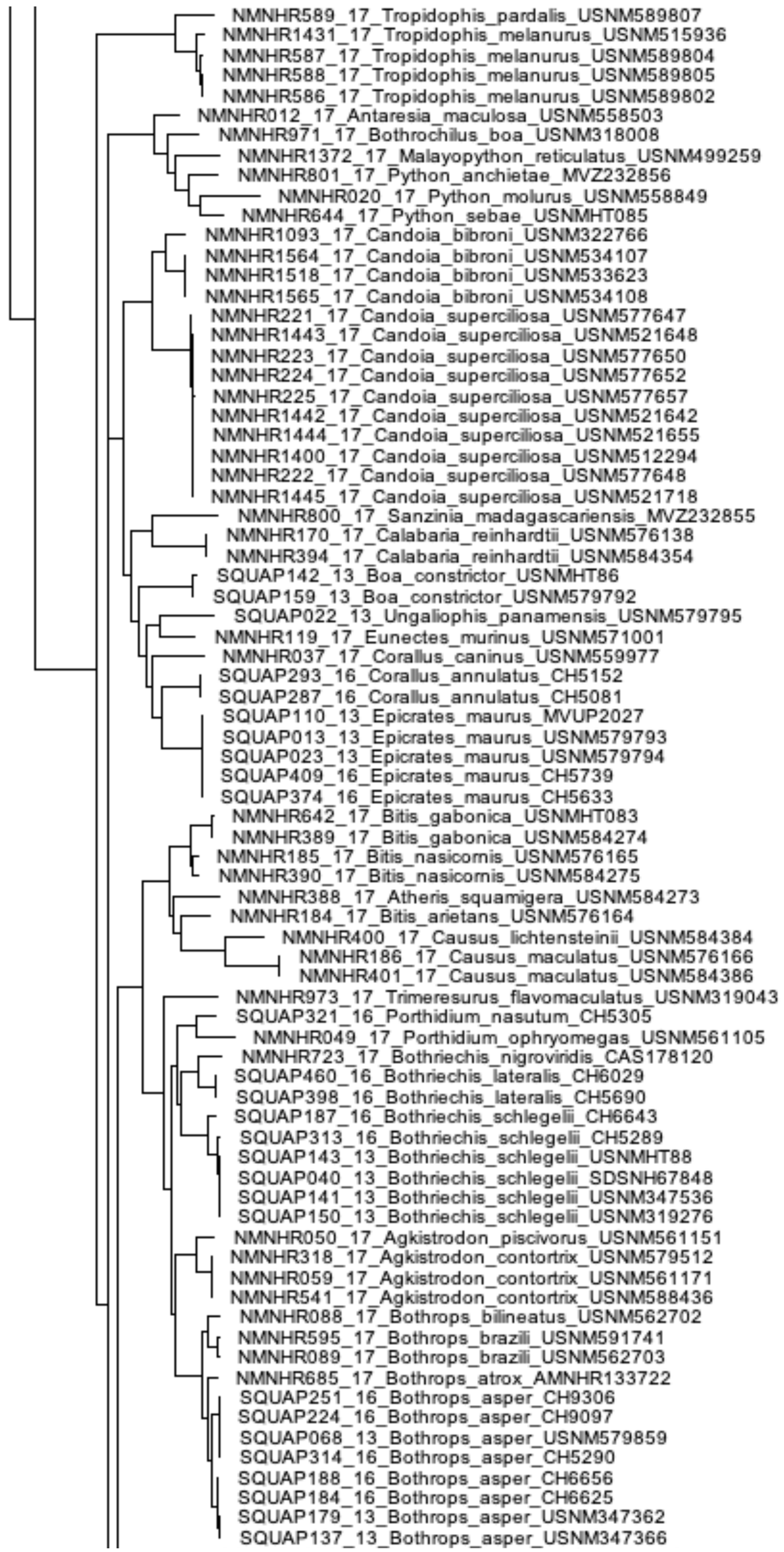

SM Figure 1.2. Tropidophiids, boas, pythons, vipers.

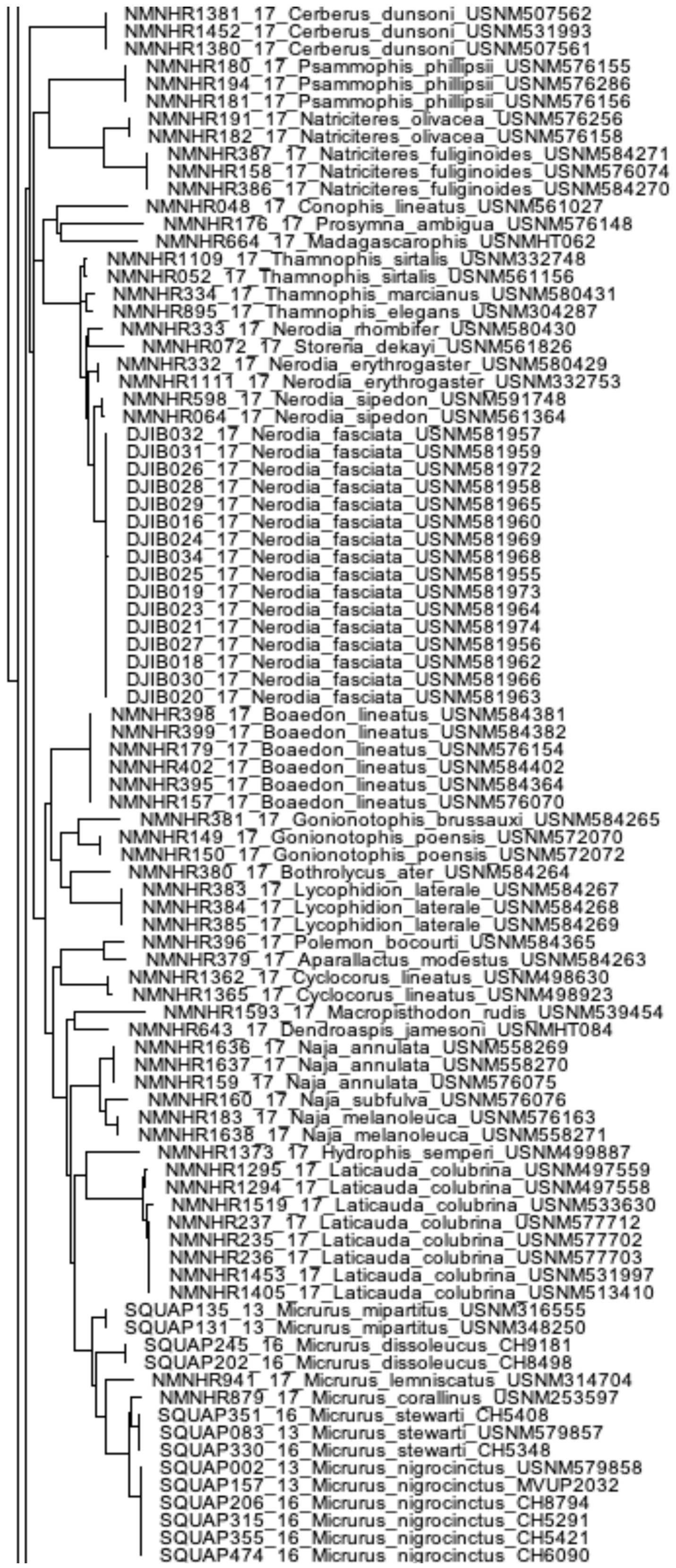

SM Figure 1.3. Homalopsids, lamprophiids, colubrids 1 (dipsadines 1 and natricines 1), elapids.

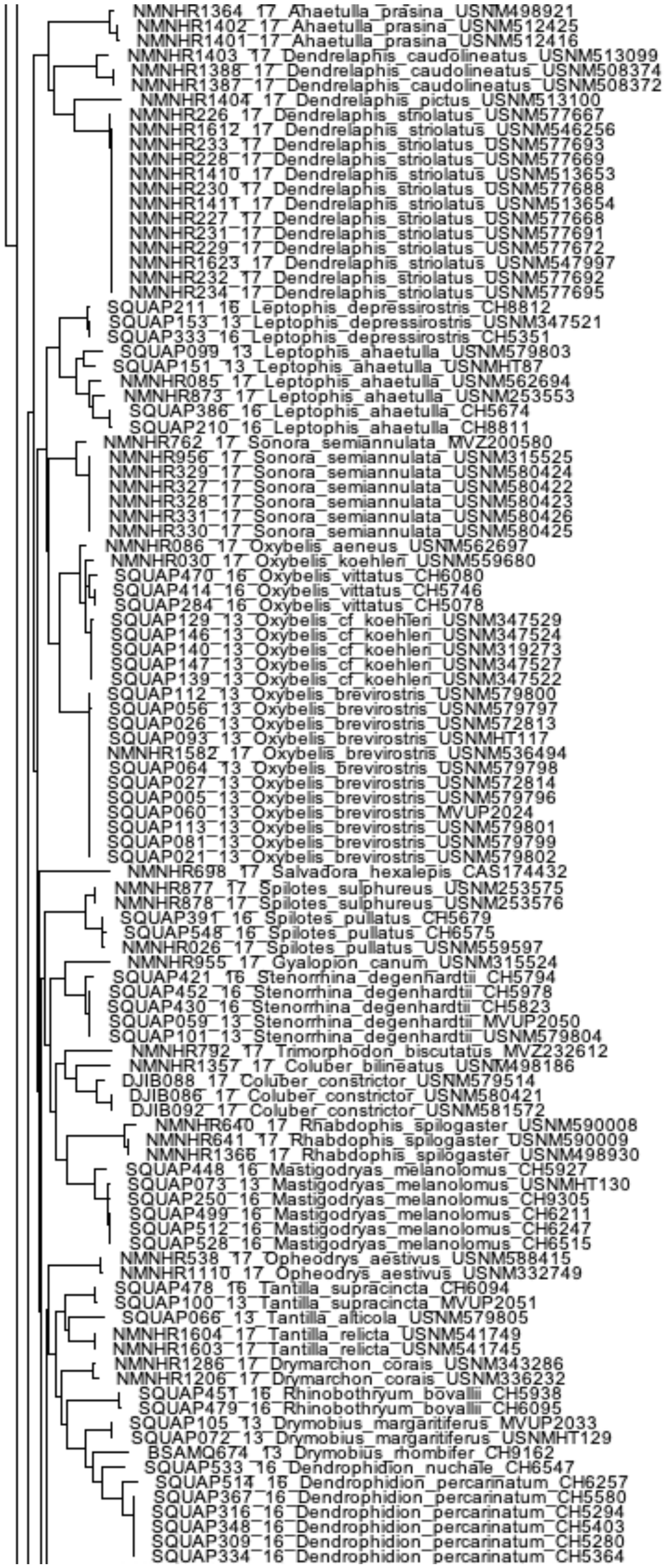

SM Figure 1.4. Colubrids 2: ahaetullines, colubrines 1, natricines.

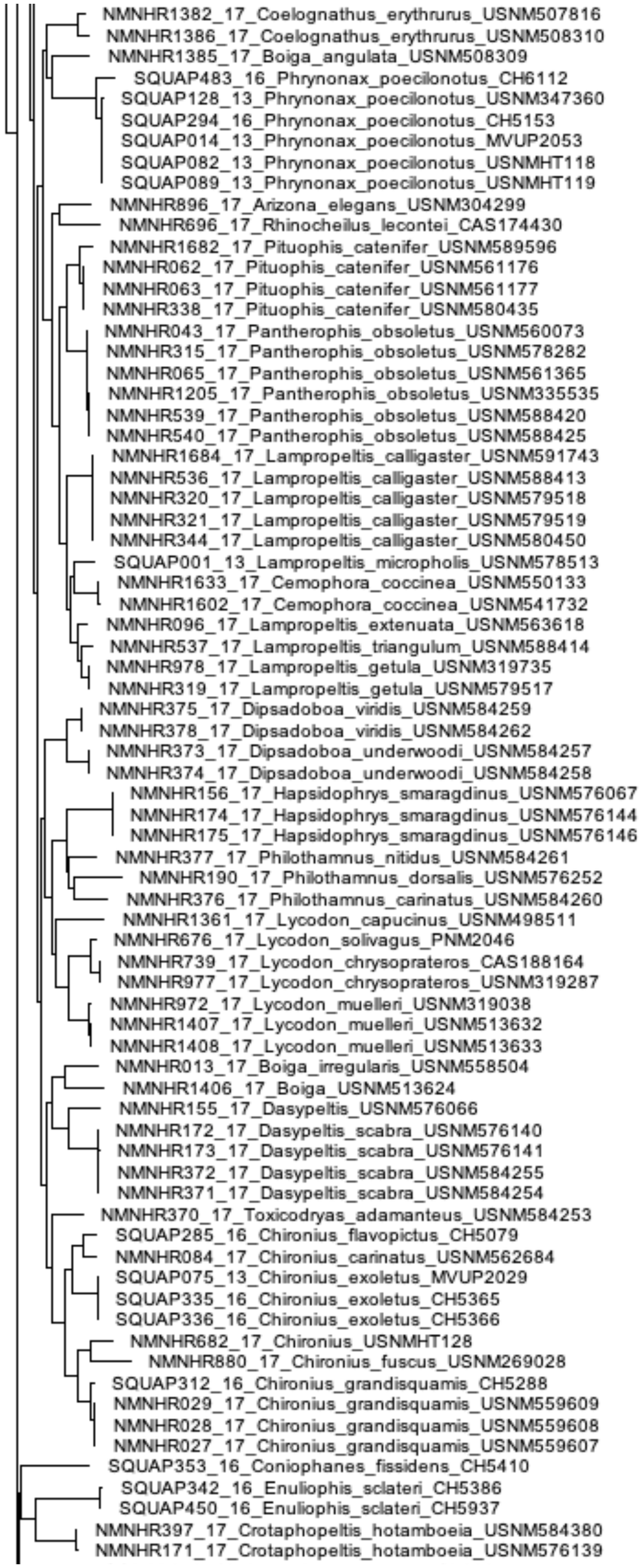

SM Figure 1.5. Colubrids 3: colubrines 2, dipsadines 2.

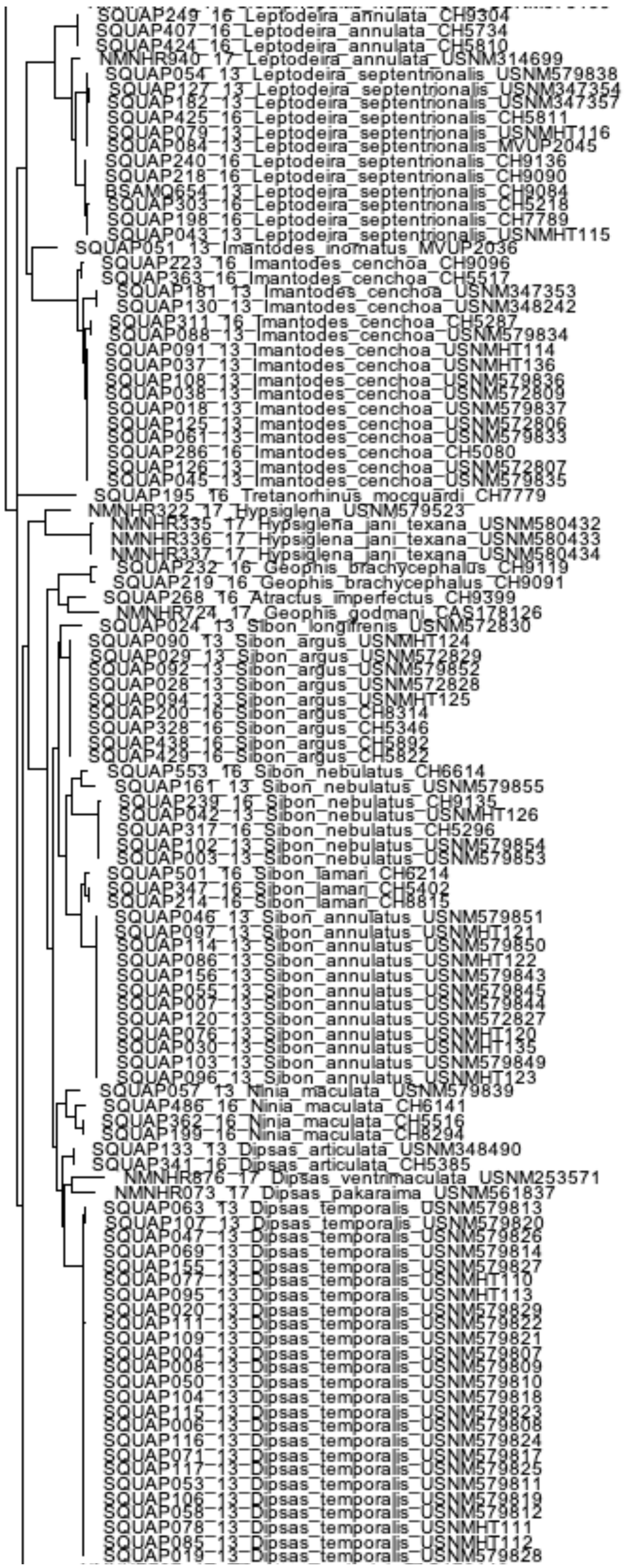

SM Figure 1.6. Colubrids 4: dipsadines 3.

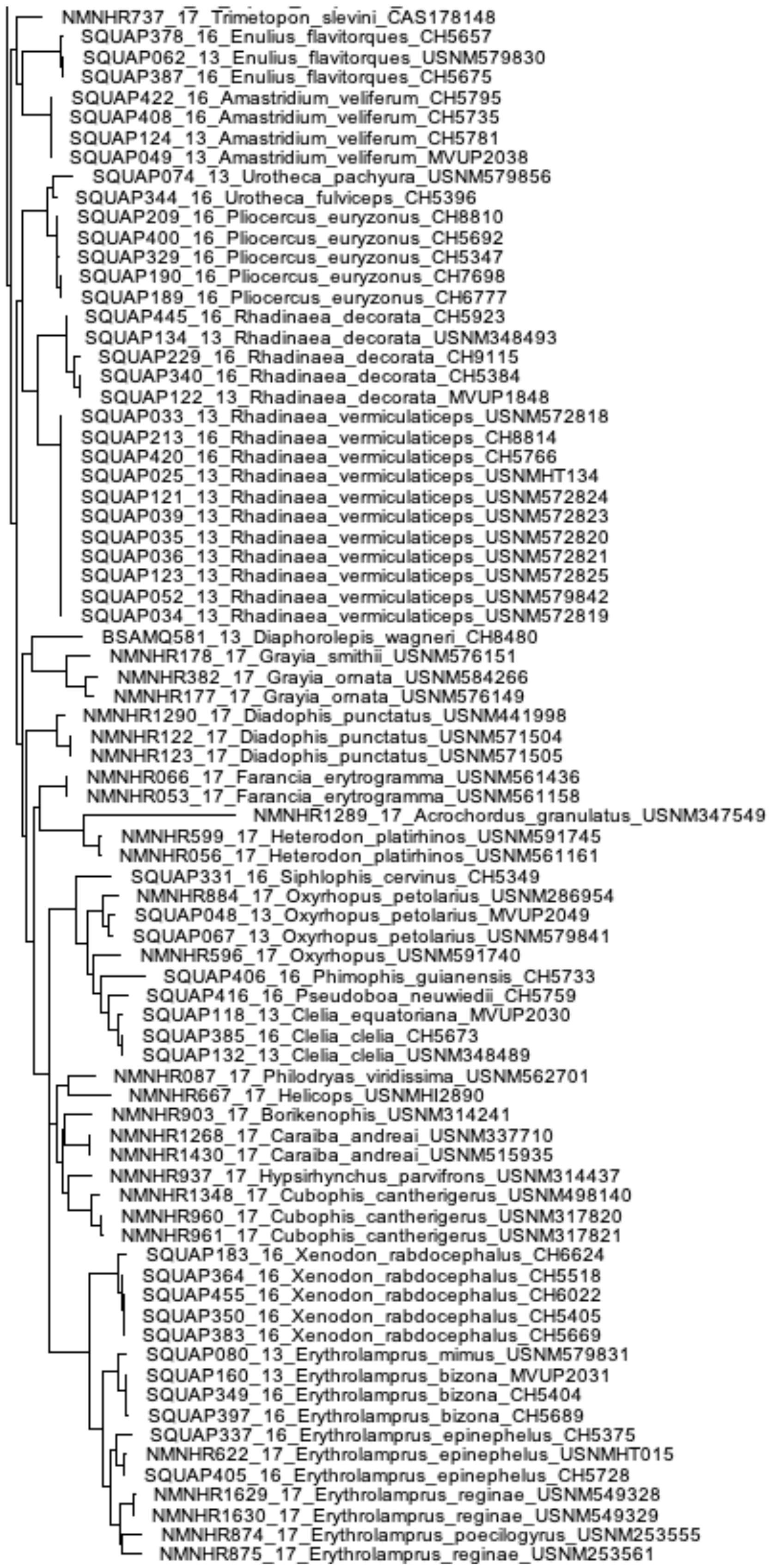

SM Figure 1.7. Colubrids 5: dipsadines 4.

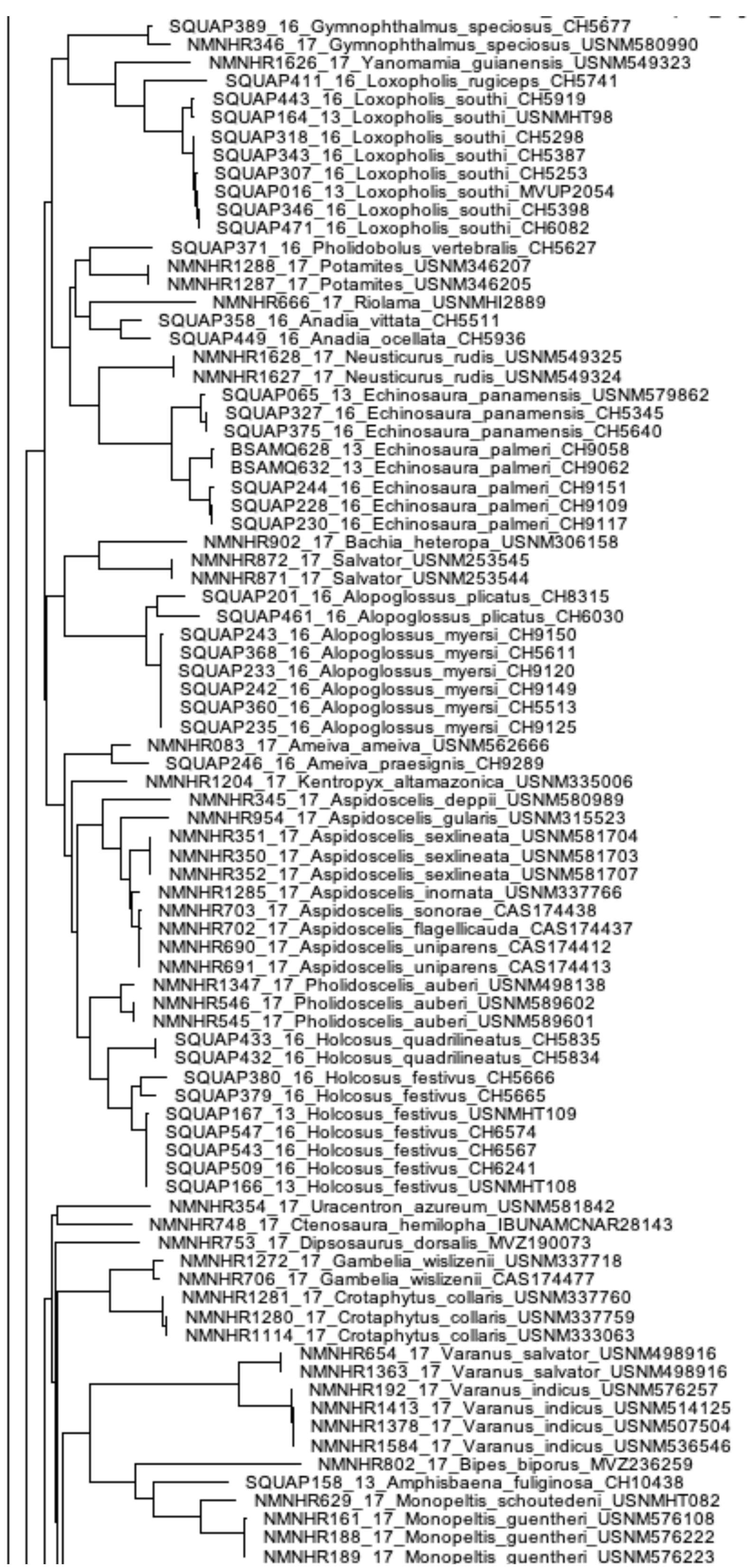

SM Figure 1.8. Gymnophthalmids, teiids, iguanas, tropidurids, crotaphytids, varanids, amphisbaenians 1.

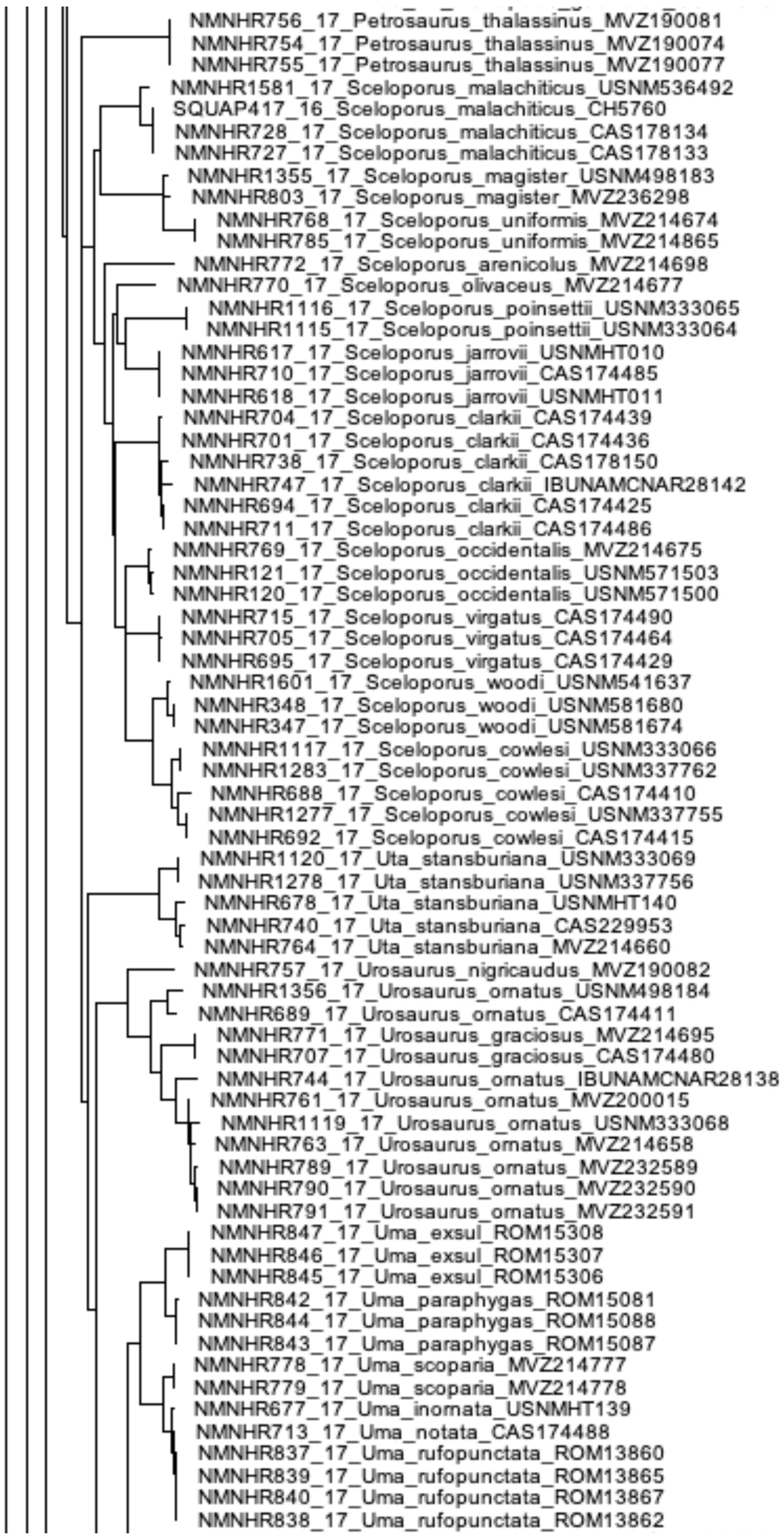

SM Figure 1.9. Phrynosomatids 1.

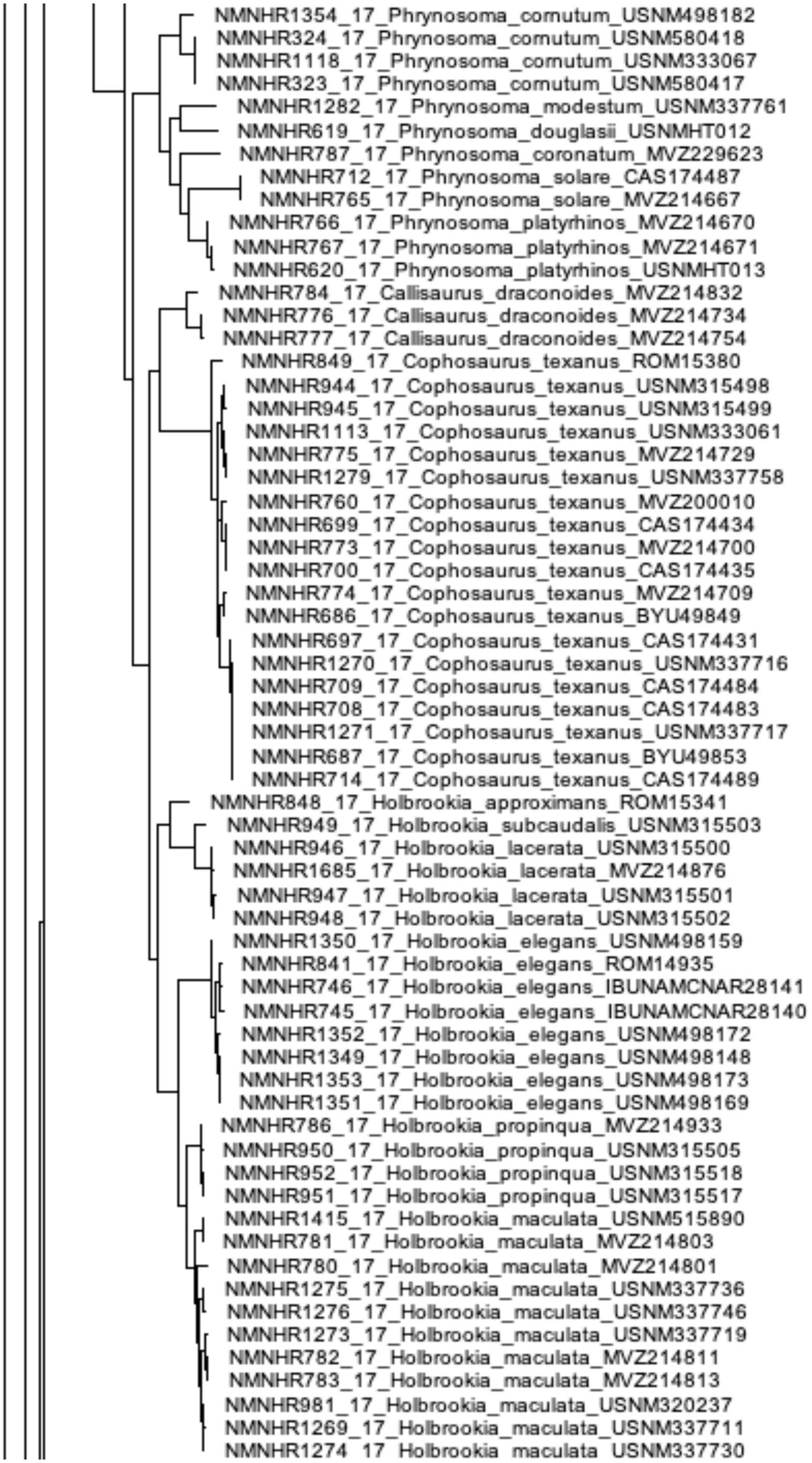

SM Figure 1.10. Phrynosomatids 2.

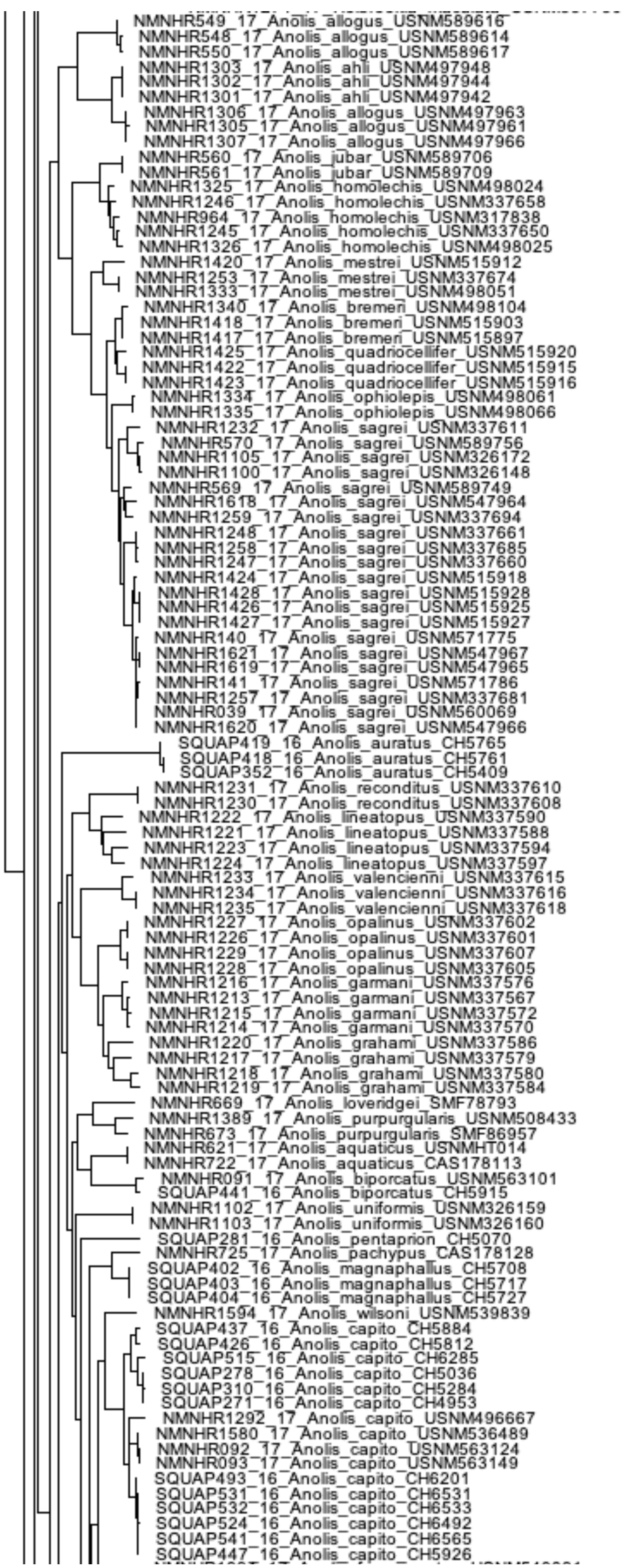

SM Figure 1.11. Anoles 1: *Trachypilus, Placopsis, Draconura* 1.

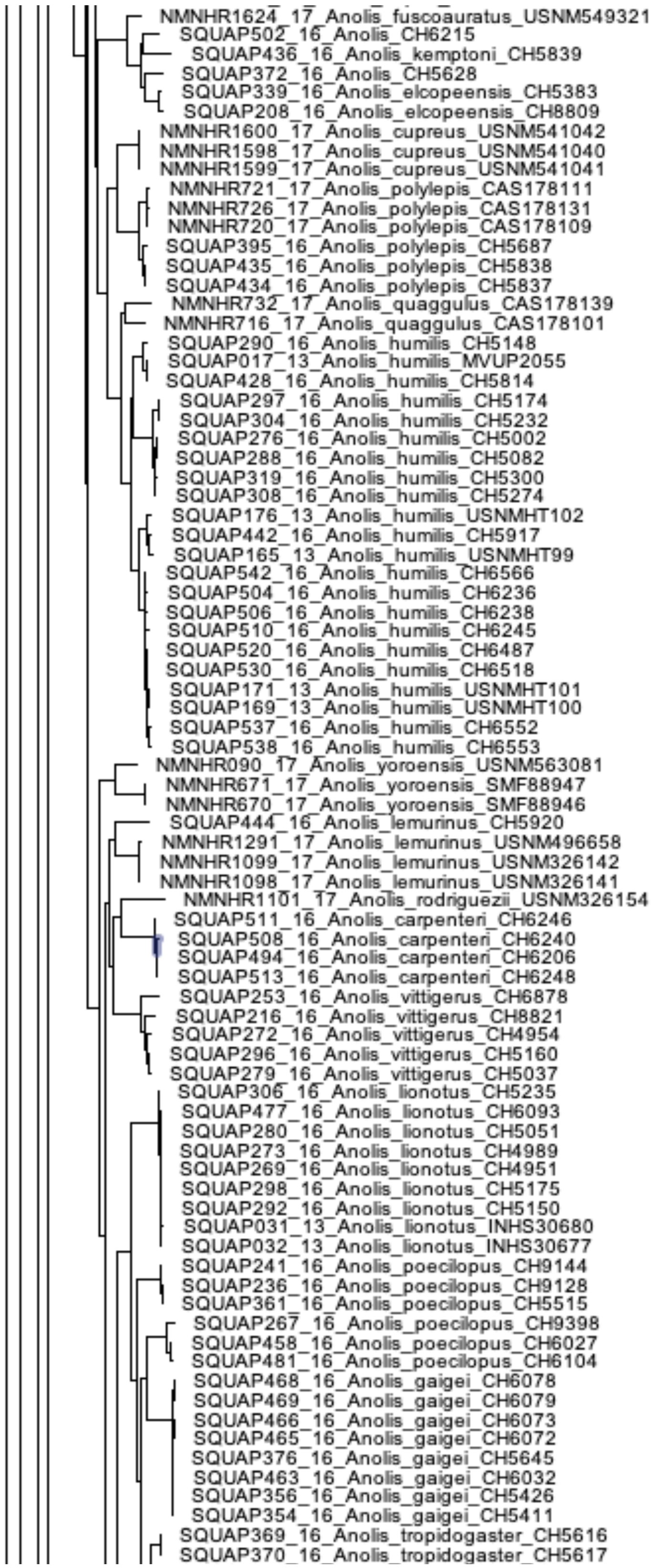

SM Figure 1.12. Anoles 2: *Draconura* 2.

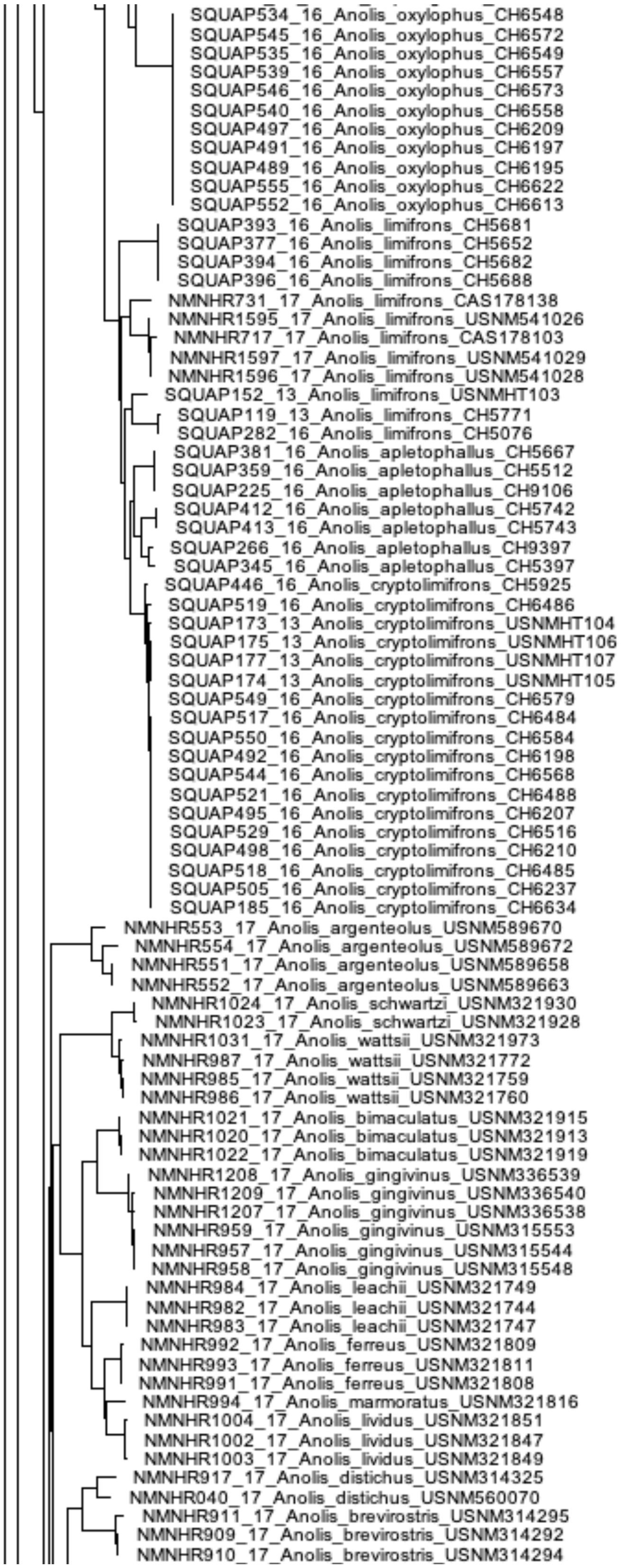

SM Figure 1.13. Anoles 3: *Draconura* 3.

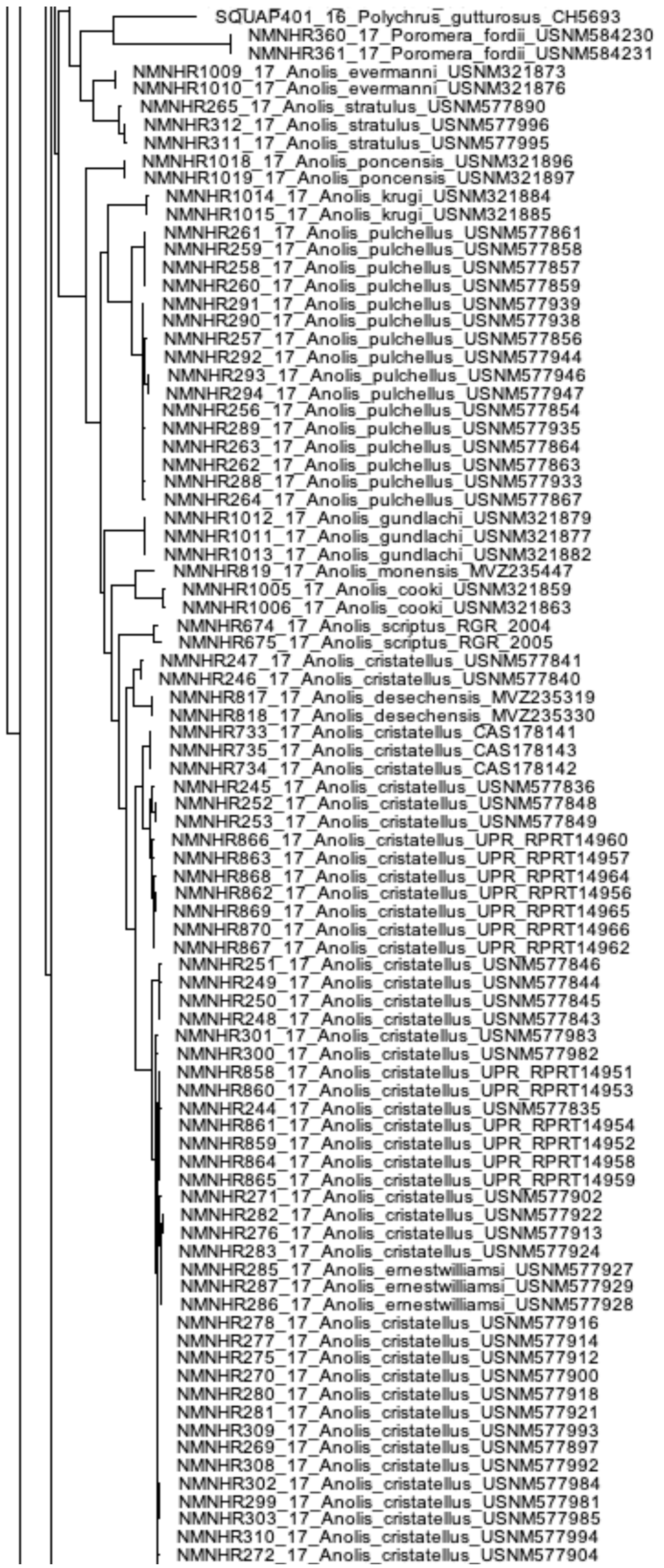

SM Figure 1.14. Anoles 4: *Polychrus*, lacertids, *Ctenonotus* 1.

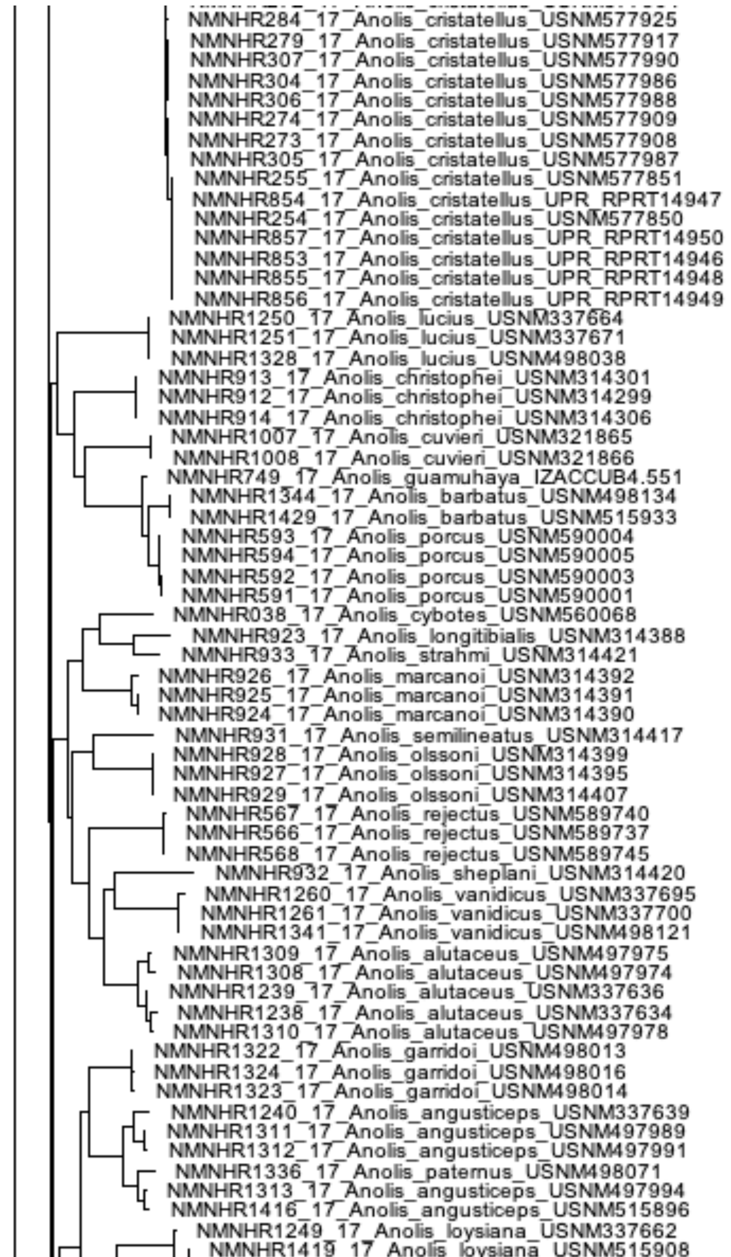

SM Figure 1.15. Anoles 5: *Ctenonotus* 2, *Ctenocercus* 1, *Xiphosurus*, *Audantia*, *Schmidtanolis* 1.

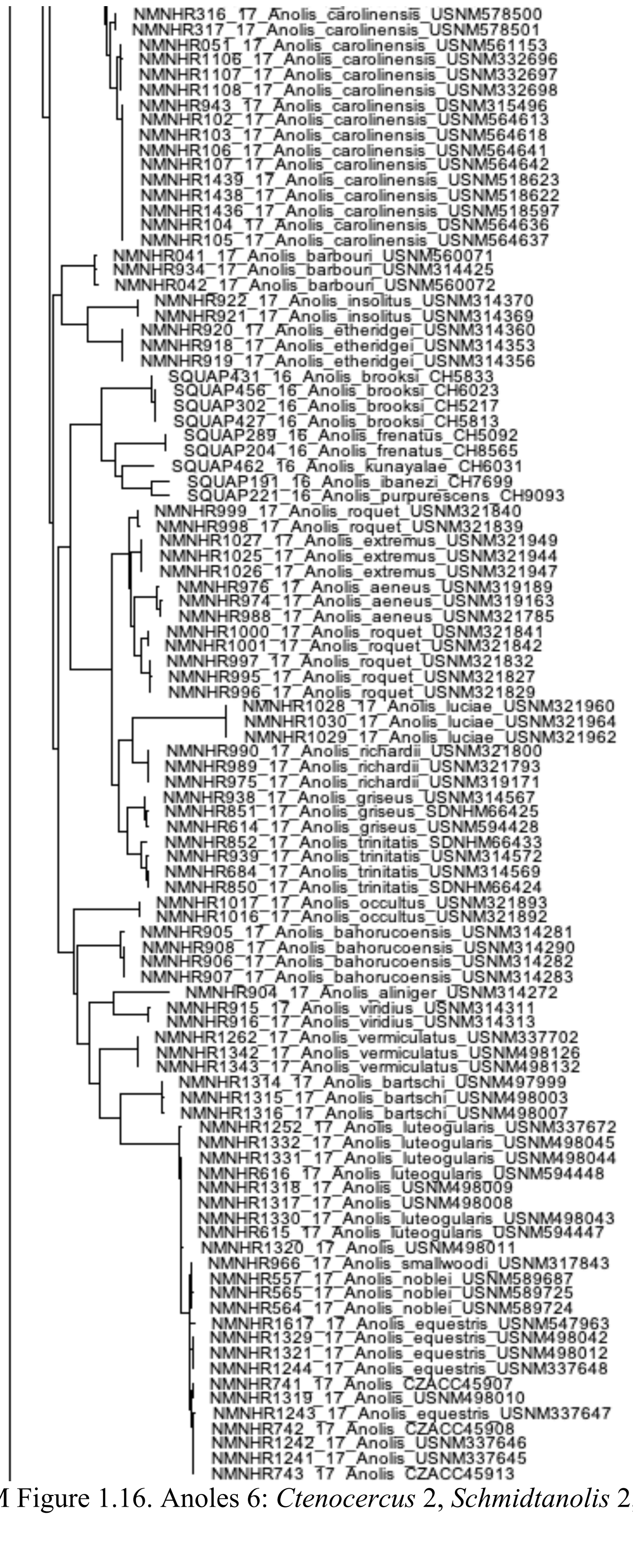

SM Figure 1.16. Anoles 6: *Ctenocercus* 2, *Schmidtanolis* 2, *Dactyloa, Deiroptyx*.

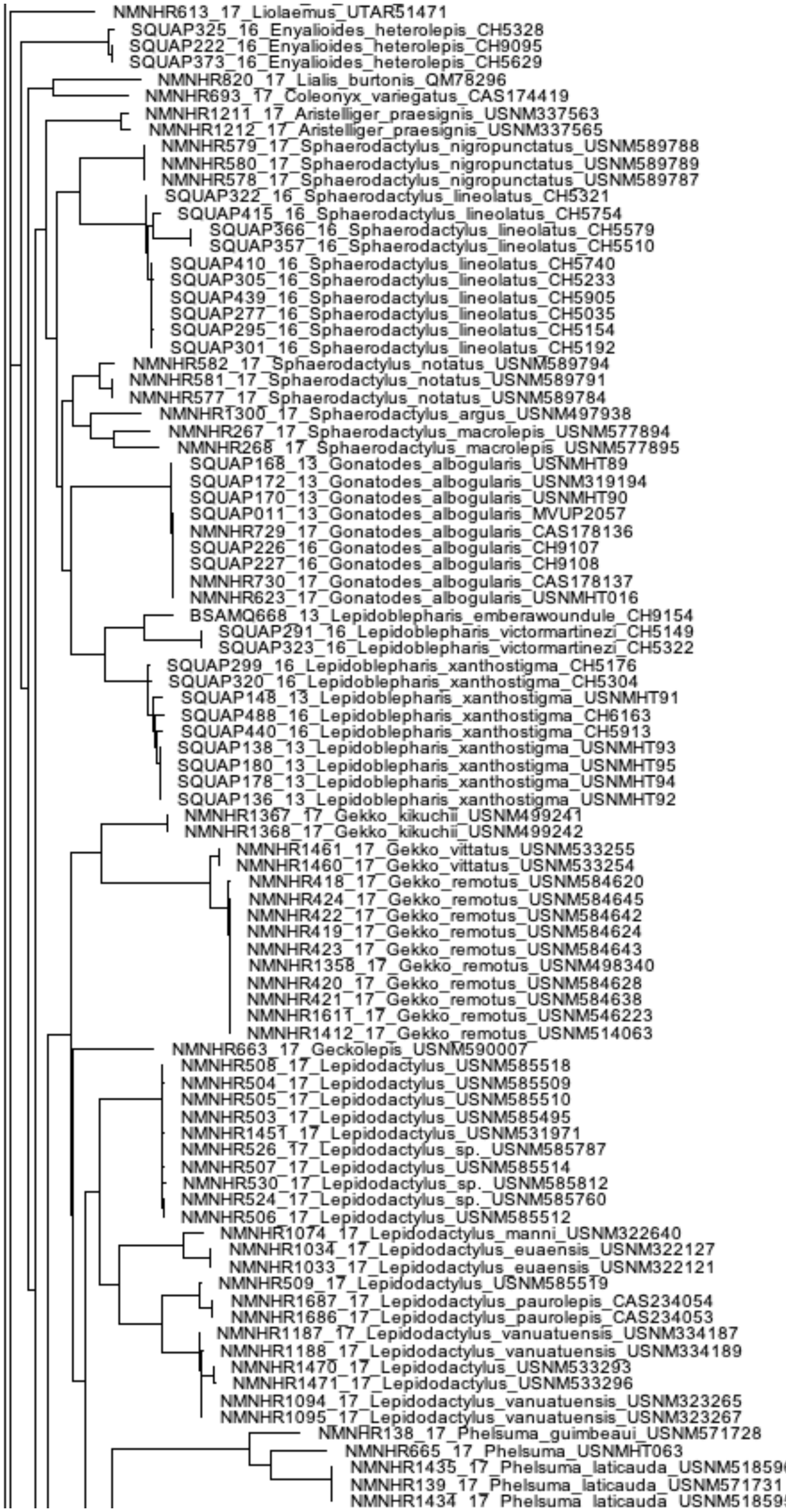

SM Figure 1.17. Liolaemid, hoplocercids, Geckos 1: (pygopodids, eublepharids, sphaerodactylids, gekkonids 1).

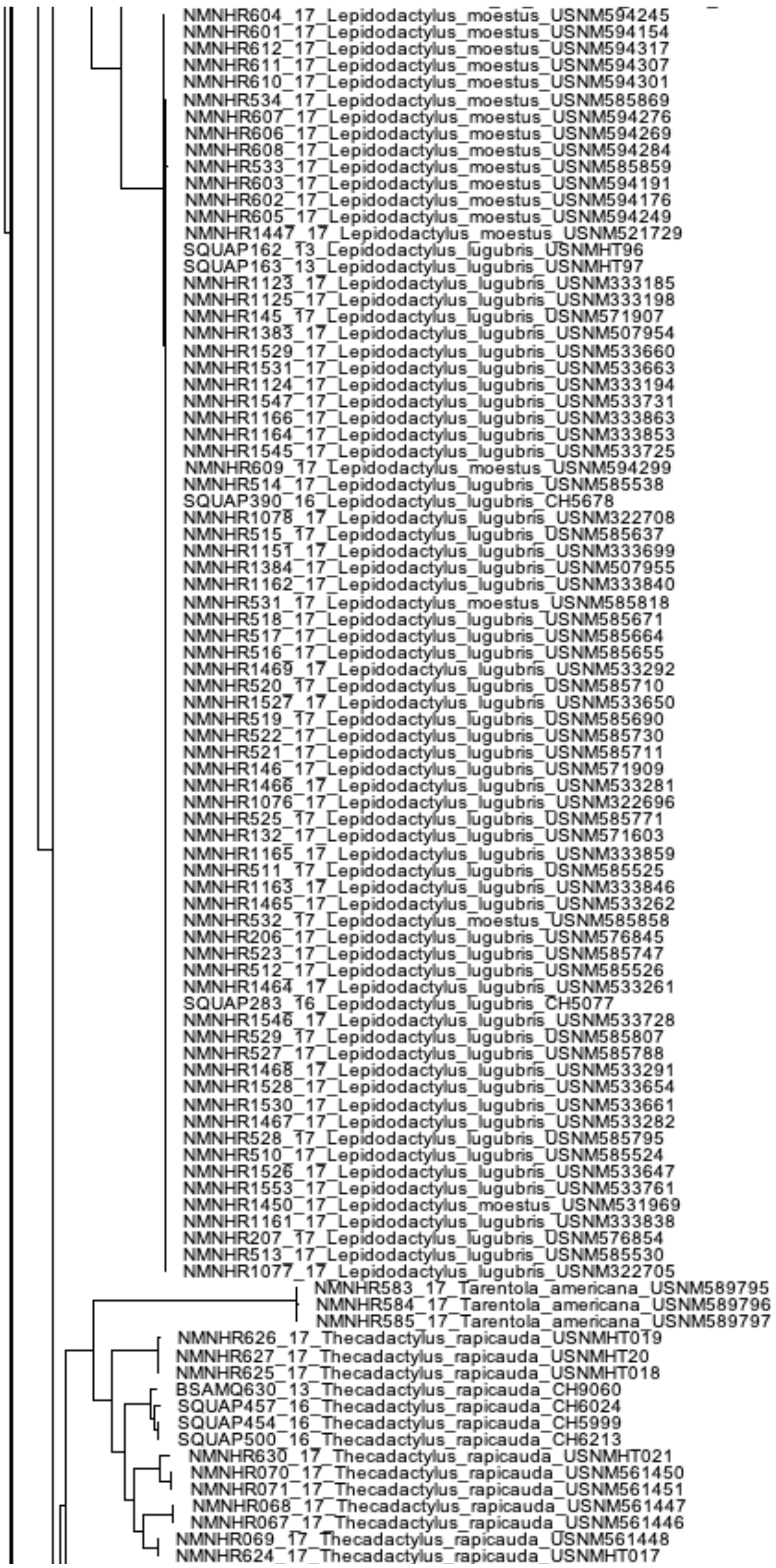

SM Figure 1.18. Geckos 2: gekkonids 2: phyllodactylids.

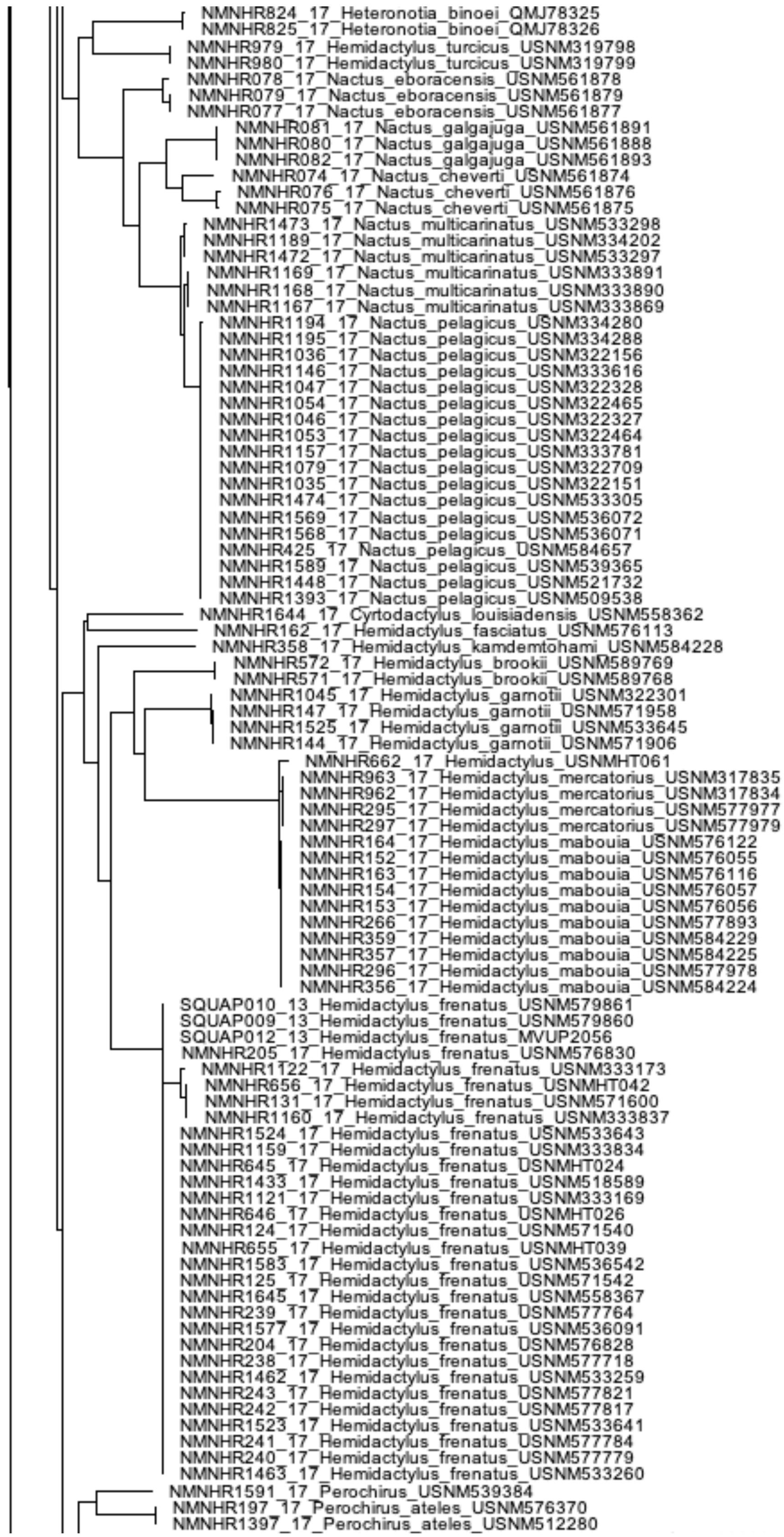

SM Figure 1.19. Geckos 3: gekkonids 3.

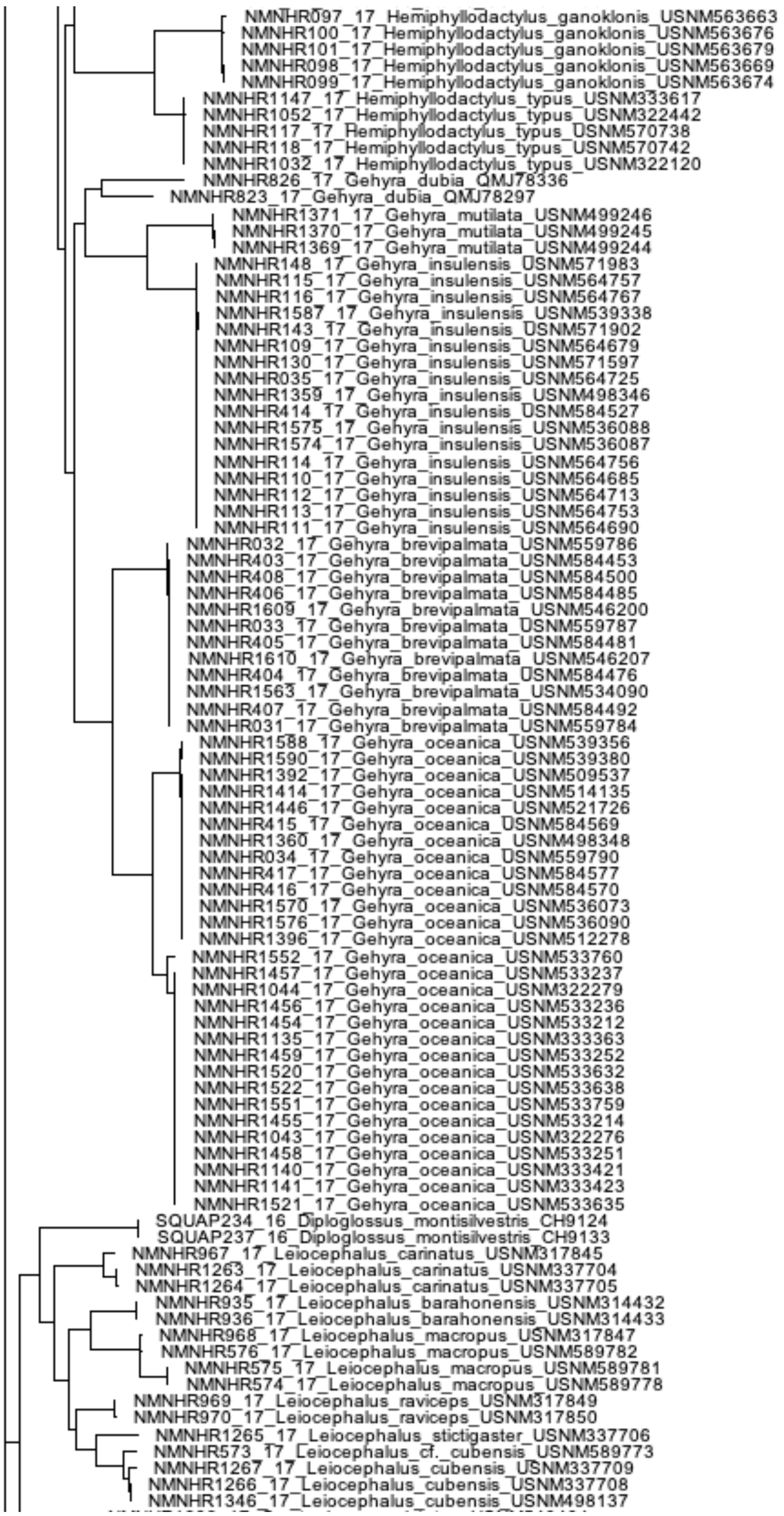

SM Figure 1.20. Geckos 4: gekkonids 4, anguids 1, leiocephalids.

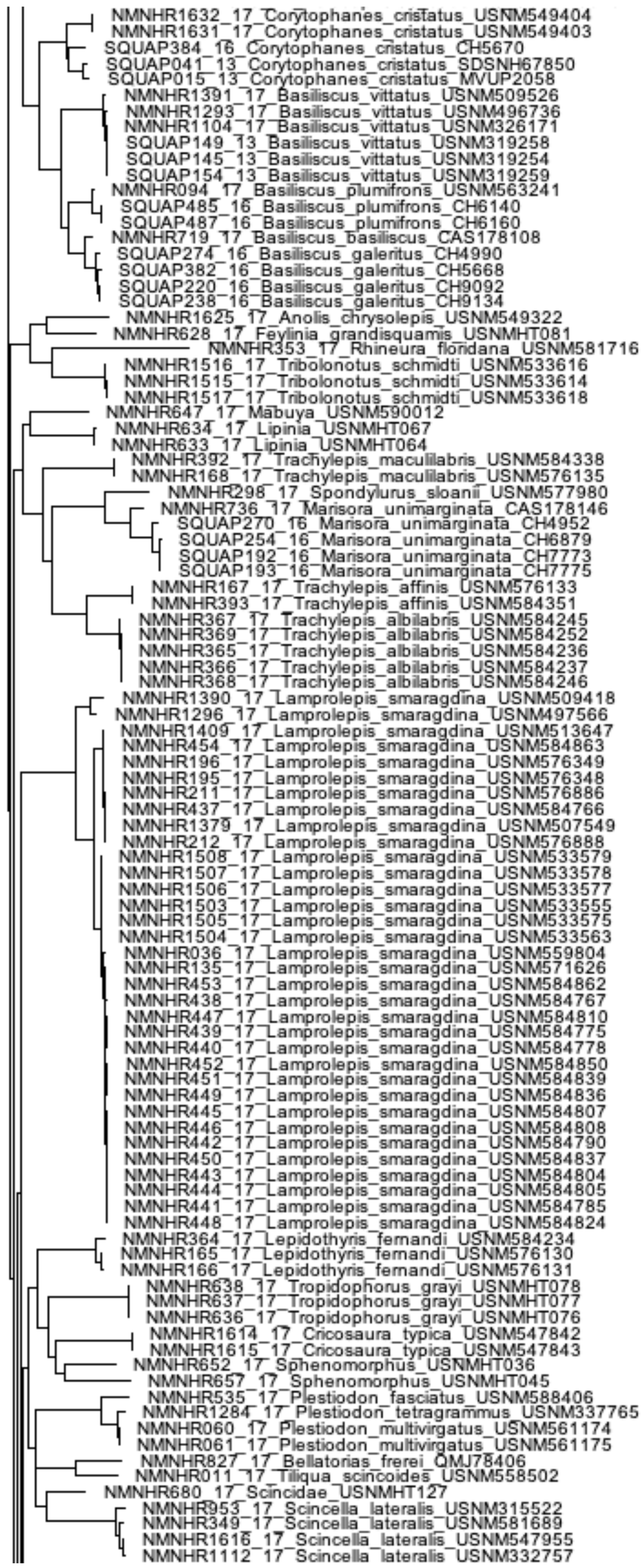

SM Figure 1.21. Corytophanids, anoles 7, amphisbaenians 2, Skinks 1: (sphenomorphines 1, mabuyinines 2), xantusiids 1.

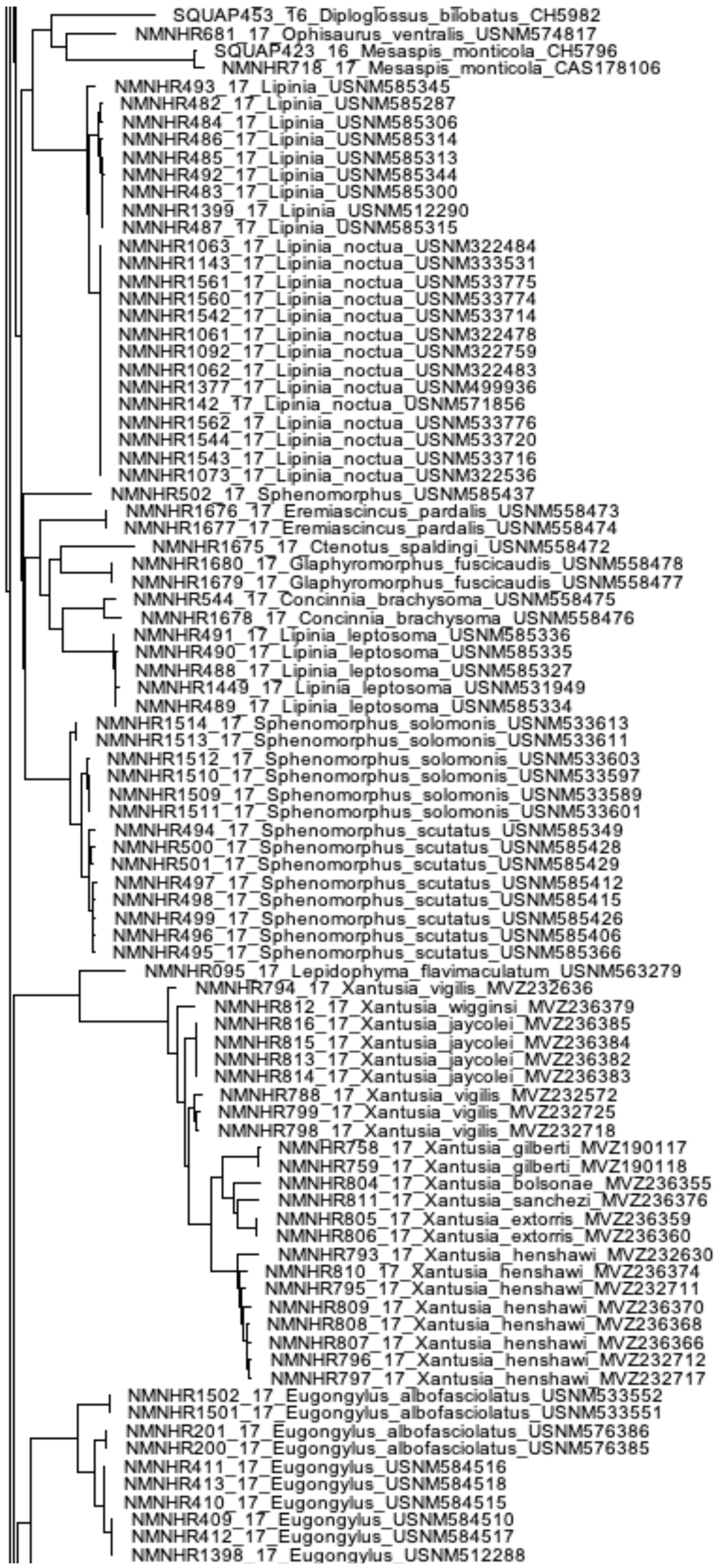

SM Figure 1.22. Anguids 2, xantusiids 2, skinks 2: (sphenomorphines 2, eugonglynines 1).

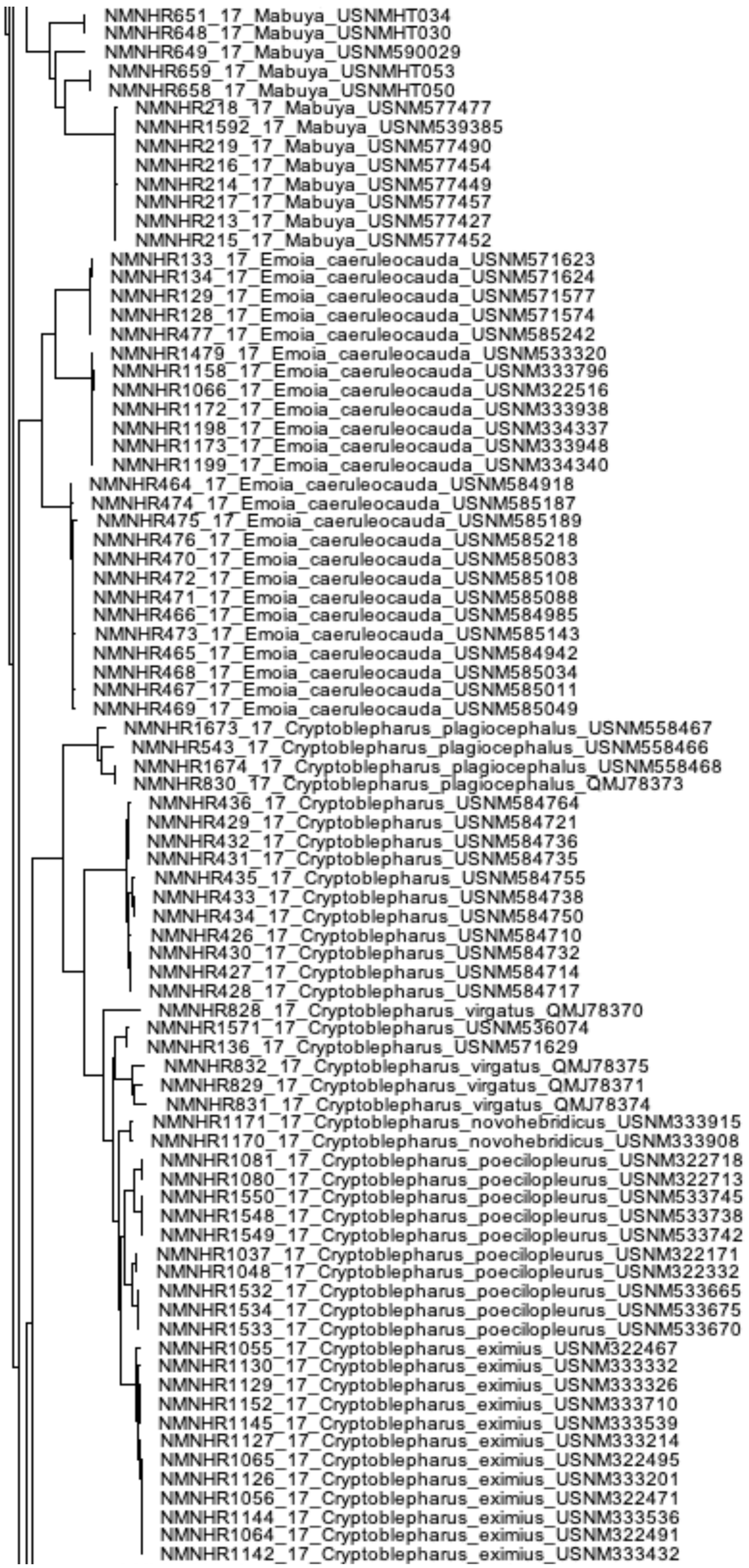

SM Figure 1.23. Skinks 3: mabuyinines 2, eugonglynines 2.

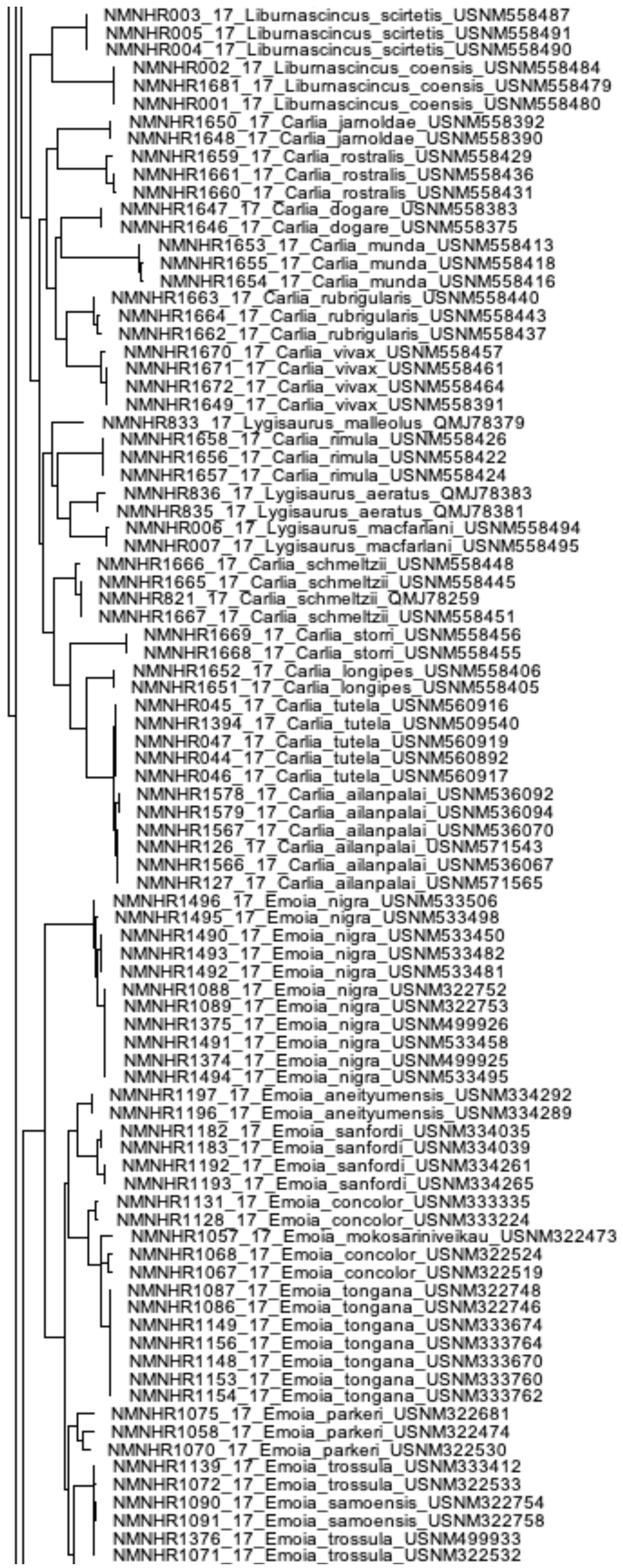

SM Figure 1.24. Skinks 4: eugonglynines 3.

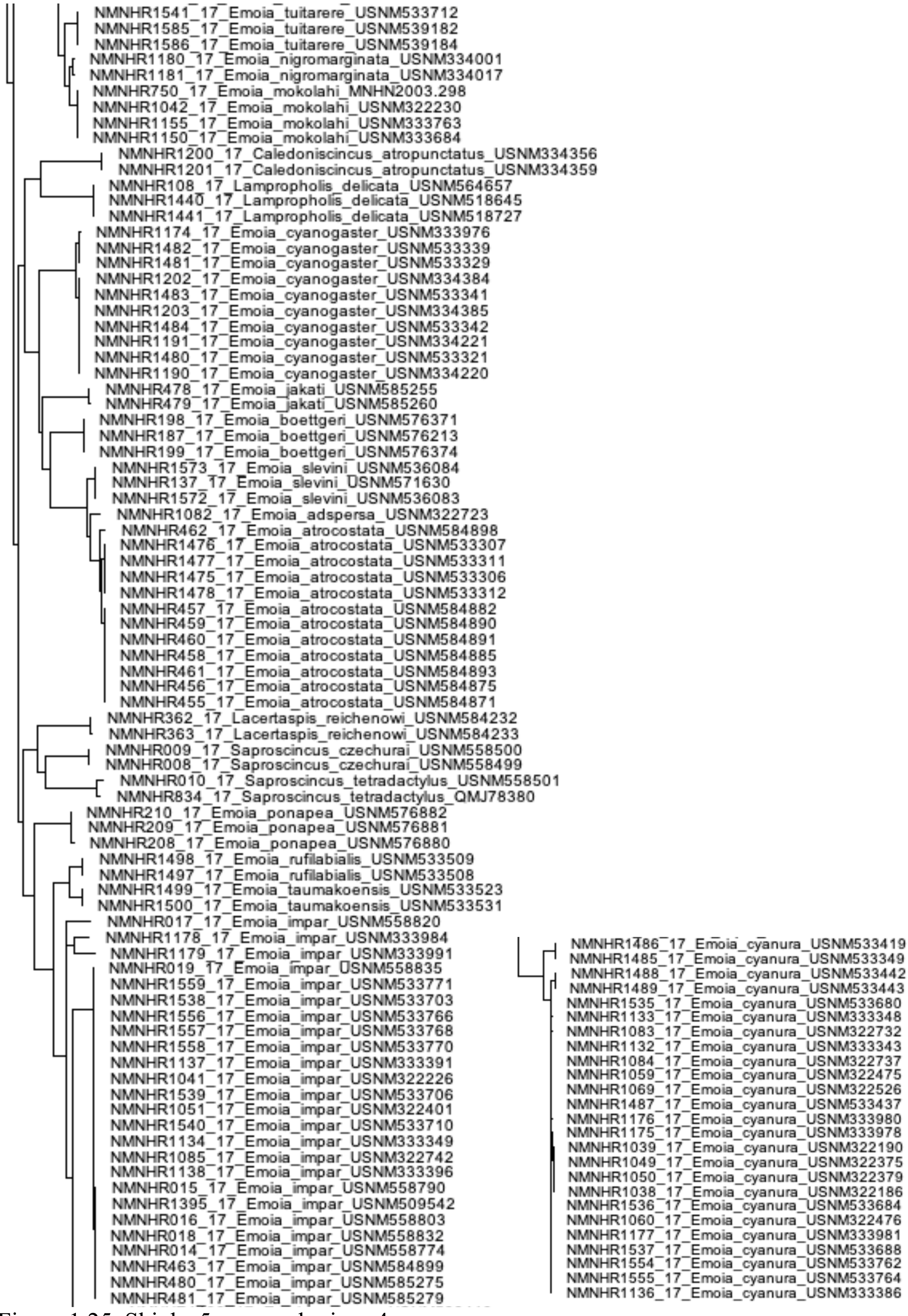

SM Figure 1.25. Skinks 5: eugonglynines 4.
