# Supplementary material for: DNA barcoding of the National Museum of Natural History reptile tissue holdings raises concerns about the use of natural history collections and the responsibilities of scientists in the molecular age
Source: PLoS One. 2022 Mar 4;17(3):e0264930. doi: 10.1371/journal.pone.0264930 (PMC8896674; doi:10.1371/journal.pone.0264930)
Supplement: S2 Fig — (PDF) [file pone.0264930.s002.pdf]

# BOLD TaxonID Tree

| | | |
|---|---|---|
| Title | : | Tree Result - DS-NMNHA |
| Date | : | 06-Dec-2021 |
| Data Type | : | Nucleotide |
| Distance Model | : | Kimura 2 Parameter |
| Marker | : | COI-5P |
| Colourization | : | [blue]=Stop Codons [red]=Contamination or misidentification |
| | | |
| Label | : | Process ID |
| Label | : | Taxon |
| | | |
| Sequence Count | : | 766 |
| Species count | : | 176 |
| Genus count | : | 2 |
| Family count | : | 2 |
| Unidentified | : | 15 |
| | | |
| BIN Count | : | 310 |

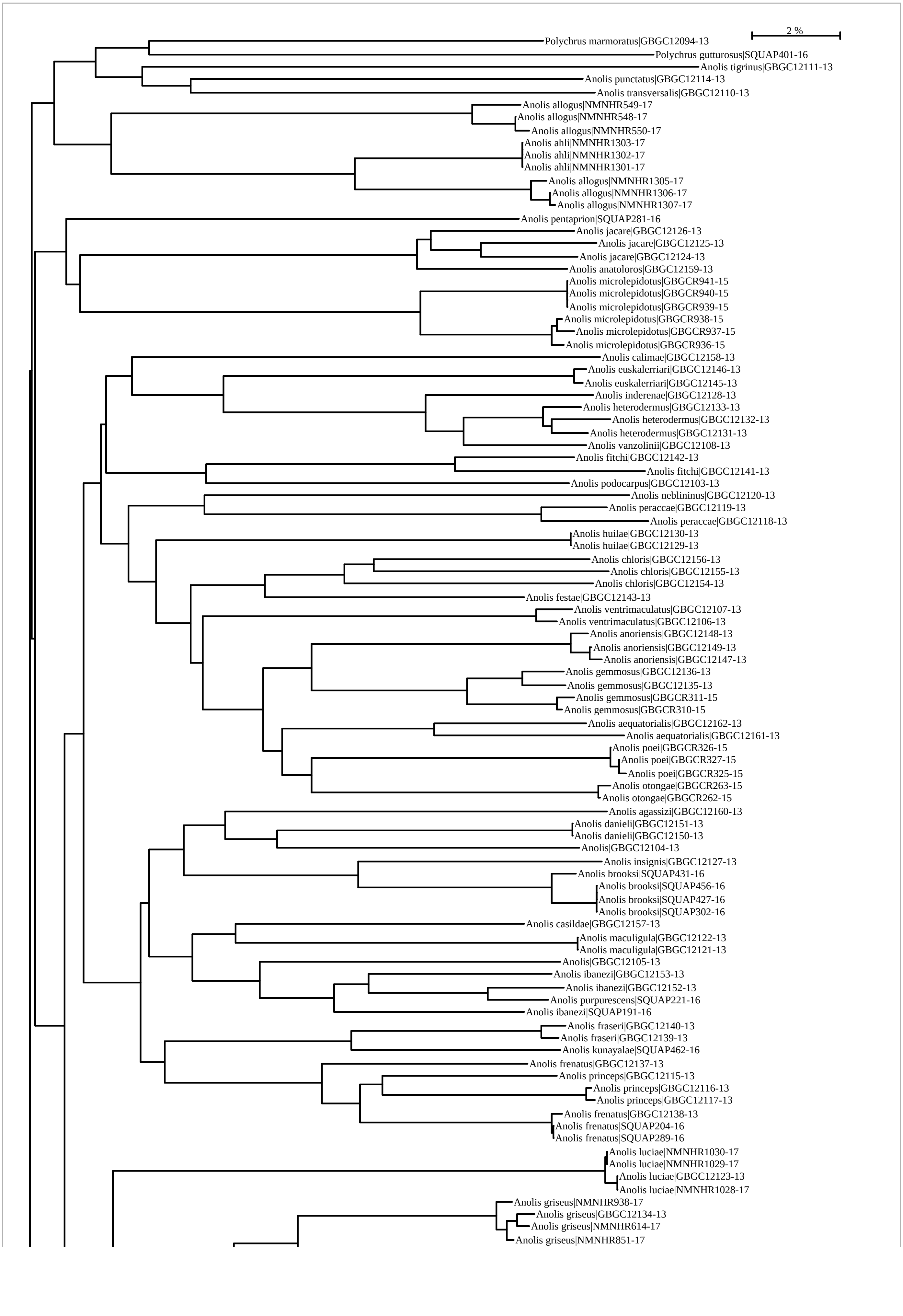
2 %
Polychrus marmoratus|GBGC12094-13
Polychrus gutturosus|SQUAP401-16
Anolis tigrinus|GBGC12111-13
Anolis punctatus|GBGC12114-13
Anolis transversalis|GBGC12110-13
Anolis allogus|NMNHR549-17
Anolis allogus|NMNHR548-17
Anolis allogus|NMNHR550-17
Anolis ahli|NMNHR1303-17
Anolis ahli|NMNHR1302-17
Anolis ahli|NMNHR1301-17
Anolis allogus|NMNHR1305-17
Anolis allogus|NMNHR1306-17
Anolis allogus|NMNHR1307-17
Anolis pentaprion|SQUAP281-16
Anolis jacare|GBGC12126-13
Anolis jacare|GBGC12125-13
Anolis jacare|GBGC12124-13
Anolis anatoloros|GBGC12159-13
Anolis microlepidotus|GBGCR941-15
Anolis microlepidotus|GBGCR940-15
Anolis microlepidotus|GBGCR939-15
Anolis microlepidotus|GBGCR938-15
Anolis microlepidotus|GBGCR937-15
Anolis microlepidotus|GBGCR936-15
Anolis calimae|GBGC12158-13
Anolis euskalerriari|GBGC12146-13
Anolis euskalerriari|GBGC12145-13
Anolis inderenae|GBGC12128-13
Anolis heterodermus|GBGC12133-13
Anolis heterodermus|GBGC12132-13
Anolis heterodermus|GBGC12131-13
Anolis vanzolinii|GBGC12108-13
Anolis fitchi|GBGC12142-13
Anolis fitchi|GBGC12141-13
Anolis podocarpus|GBGC12103-13
Anolis neblininus|GBGC12120-13
Anolis peraccae|GBGC12119-13
Anolis peraccae|GBGC12118-13
Anolis huilae|GBGC12130-13
Anolis huilae|GBGC12129-13
Anolis chloris|GBGC12156-13
Anolis chloris|GBGC12155-13
Anolis chloris|GBGC12154-13
Anolis festae|GBGC12143-13
Anolis ventrimaculatus|GBGC12107-13
Anolis ventrimaculatus|GBGC12106-13
Anolis anoriensis|GBGC12148-13
Anolis anoriensis|GBGC12149-13
Anolis anoriensis|GBGC12147-13
Anolis gemmosus|GBGC12136-13
Anolis gemmosus|GBGC12135-13
Anolis gemmosus|GBGCR311-15
Anolis gemmosus|GBGCR310-15
Anolis aequatorialis|GBGC12162-13
Anolis aequatorialis|GBGC12161-13
Anolis poei|GBGCR326-15
Anolis poei|GBGCR327-15
Anolis poei|GBGCR325-15
Anolis otongae|GBGCR263-15
Anolis otongae|GBGCR262-15
Anolis agassizi|GBGC12160-13
Anolis danieli|GBGC12151-13
Anolis danieli|GBGC12150-13
Anolis|GBGC12104-13
Anolis insignis|GBGC12127-13
Anolis brooksi|SQUAP431-16
Anolis brooksi|SQUAP456-16
Anolis brooksi|SQUAP427-16
Anolis brooksi|SQUAP302-16
Anolis casildae|GBGC12157-13
Anolis maculigula|GBGC12122-13
Anolis maculigula|GBGC12121-13
Anolis|GBGC12105-13
Anolis ibanezi|GBGC12153-13
Anolis ibanezi|GBGC12152-13
Anolis purpurescens|SQUAP221-16
Anolis ibanezi|SQUAP191-16
Anolis fraseri|GBGC12140-13
Anolis fraseri|GBGC12139-13
Anolis kunayalae|SQUAP462-16
Anolis frenatus|GBGC12137-13
Anolis princeps|GBGC12115-13
Anolis princeps|GBGC12116-13
Anolis princeps|GBGC12117-13
Anolis frenatus|GBGC12138-13
Anolis frenatus|SQUAP204-16
Anolis frenatus|SQUAP289-16
Anolis luciae|NMNHR1030-17
Anolis luciae|NMNHR1029-17
Anolis luciae|GBGC12123-13
Anolis luciae|NMNHR1028-17
Anolis griseus|NMNHR938-17
Anolis griseus|GBGC12134-13
Anolis griseus|NMNHR614-17
Anolis griseus|NMNHR851-17

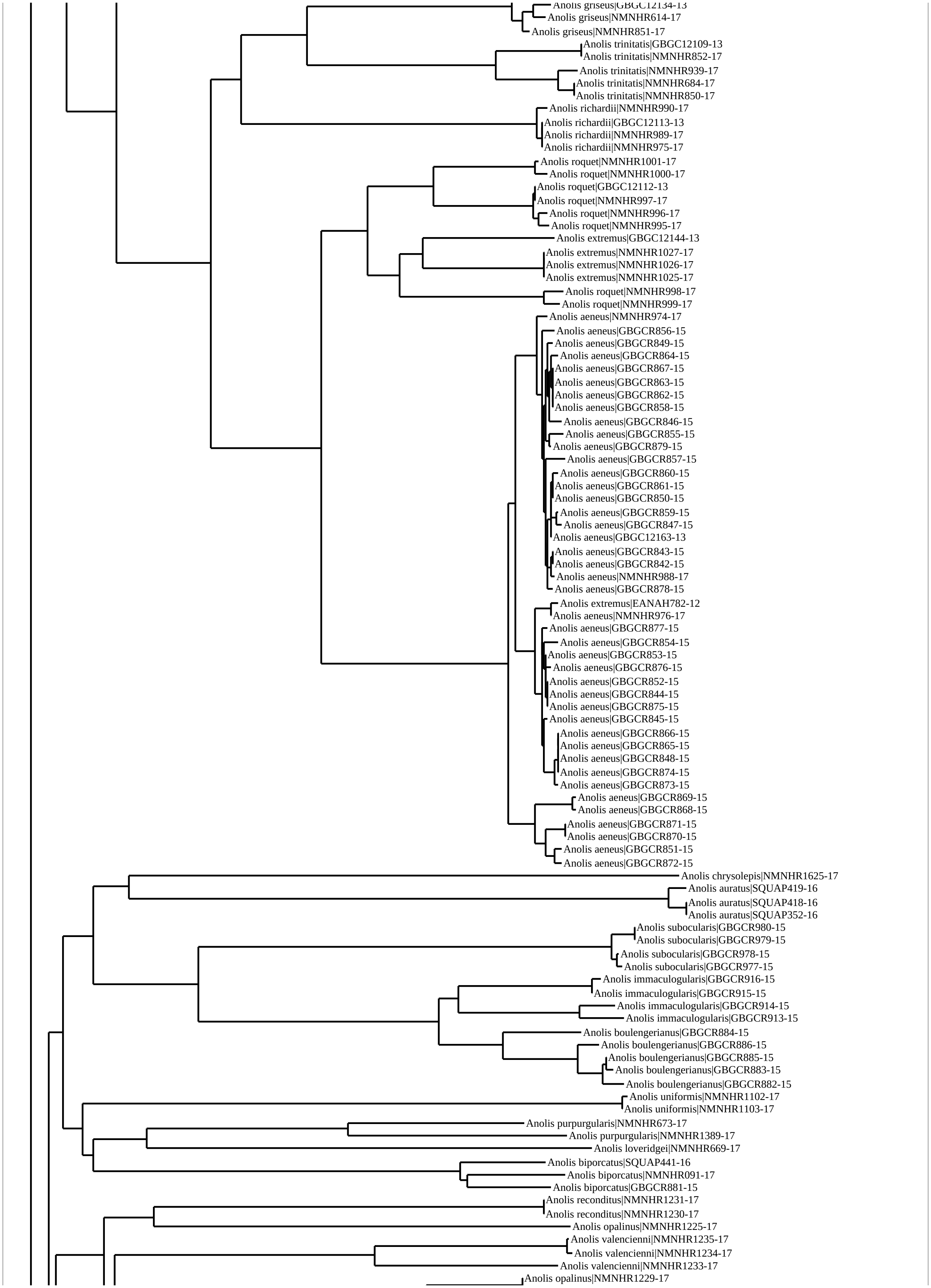
Anolis griseus|GBGC12134-13
Anolis griseus|NMNHR614-17
Anolis griseus|NMNHR851-17
Anolis trinitatis|GBGC12109-13
Anolis trinitatis|NMNHR852-17
Anolis trinitatis|NMNHR939-17
Anolis trinitatis|NMNHR684-17
Anolis trinitatis|NMNHR850-17
Anolis richardii|NMNHR990-17
Anolis richardii|GBGC12113-13
Anolis richardii|NMNHR989-17
Anolis richardii|NMNHR975-17
Anolis roquet|NMNHR1001-17
Anolis roquet|NMNHR1000-17
Anolis roquet|GBGC12112-13
Anolis roquet|NMNHR997-17
Anolis roquet|NMNHR996-17
Anolis roquet|NMNHR995-17
Anolis extremus|GBGC12144-13
Anolis extremus|NMNHR1027-17
Anolis extremus|NMNHR1026-17
Anolis extremus|NMNHR1025-17
Anolis roquet|NMNHR998-17
Anolis roquet|NMNHR999-17
Anolis aeneus|NMNHR974-17
Anolis aeneus|GBGCR856-15
Anolis aeneus|GBGCR849-15
Anolis aeneus|GBGCR864-15
Anolis aeneus|GBGCR867-15
Anolis aeneus|GBGCR863-15
Anolis aeneus|GBGCR862-15
Anolis aeneus|GBGCR858-15
Anolis aeneus|GBGCR846-15
Anolis aeneus|GBGCR855-15
Anolis aeneus|GBGCR879-15
Anolis aeneus|GBGCR857-15
Anolis aeneus|GBGCR860-15
Anolis aeneus|GBGCR861-15
Anolis aeneus|GBGCR850-15
Anolis aeneus|GBGCR859-15
Anolis aeneus|GBGCR847-15
Anolis aeneus|GBGC12163-13
Anolis aeneus|GBGCR843-15
Anolis aeneus|GBGCR842-15
Anolis aeneus|NMNHR988-17
Anolis aeneus|GBGCR878-15
Anolis extremus|EANAH782-12
Anolis aeneus|NMNHR976-17
Anolis aeneus|GBGCR877-15
Anolis aeneus|GBGCR854-15
Anolis aeneus|GBGCR853-15
Anolis aeneus|GBGCR876-15
Anolis aeneus|GBGCR852-15
Anolis aeneus|GBGCR844-15
Anolis aeneus|GBGCR875-15
Anolis aeneus|GBGCR845-15
Anolis aeneus|GBGCR866-15
Anolis aeneus|GBGCR865-15
Anolis aeneus|GBGCR848-15
Anolis aeneus|GBGCR874-15
Anolis aeneus|GBGCR873-15
Anolis aeneus|GBGCR869-15
Anolis aeneus|GBGCR868-15
Anolis aeneus|GBGCR871-15
Anolis aeneus|GBGCR870-15
Anolis aeneus|GBGCR851-15
Anolis aeneus|GBGCR872-15
Anolis chrysolepis|NMNHR1625-17
Anolis auratus|SQUAP419-16
Anolis auratus|SQUAP418-16
Anolis auratus|SQUAP352-16
Anolis subocularis|GBGCR980-15
Anolis subocularis|GBGCR979-15
Anolis subocularis|GBGCR978-15
Anolis subocularis|GBGCR977-15
Anolis immaculogularis|GBGCR916-15
Anolis immaculogularis|GBGCR915-15
Anolis immaculogularis|GBGCR914-15
Anolis immaculogularis|GBGCR913-15
Anolis boulengerianus|GBGCR884-15
Anolis boulengerianus|GBGCR886-15
Anolis boulengerianus|GBGCR885-15
Anolis boulengerianus|GBGCR883-15
Anolis boulengerianus|GBGCR882-15
Anolis uniformis|NMNHR1102-17
Anolis uniformis|NMNHR1103-17
Anolis purpurgularis|NMNHR673-17
Anolis purpurgularis|NMNHR1389-17
Anolis loveridgei|NMNHR669-17
Anolis biporcatus|SQUAP441-16
Anolis biporcatus|NMNHR091-17
Anolis biporcatus|GBGCR881-15
Anolis reconditus|NMNHR1231-17
Anolis reconditus|NMNHR1230-17
Anolis opalinus|NMNHR1225-17
Anolis valencienni|NMNHR1235-17
Anolis valencienni|NMNHR1234-17
Anolis valencienni|NMNHR1233-17
Anolis opalinus|NMNHR1229-17

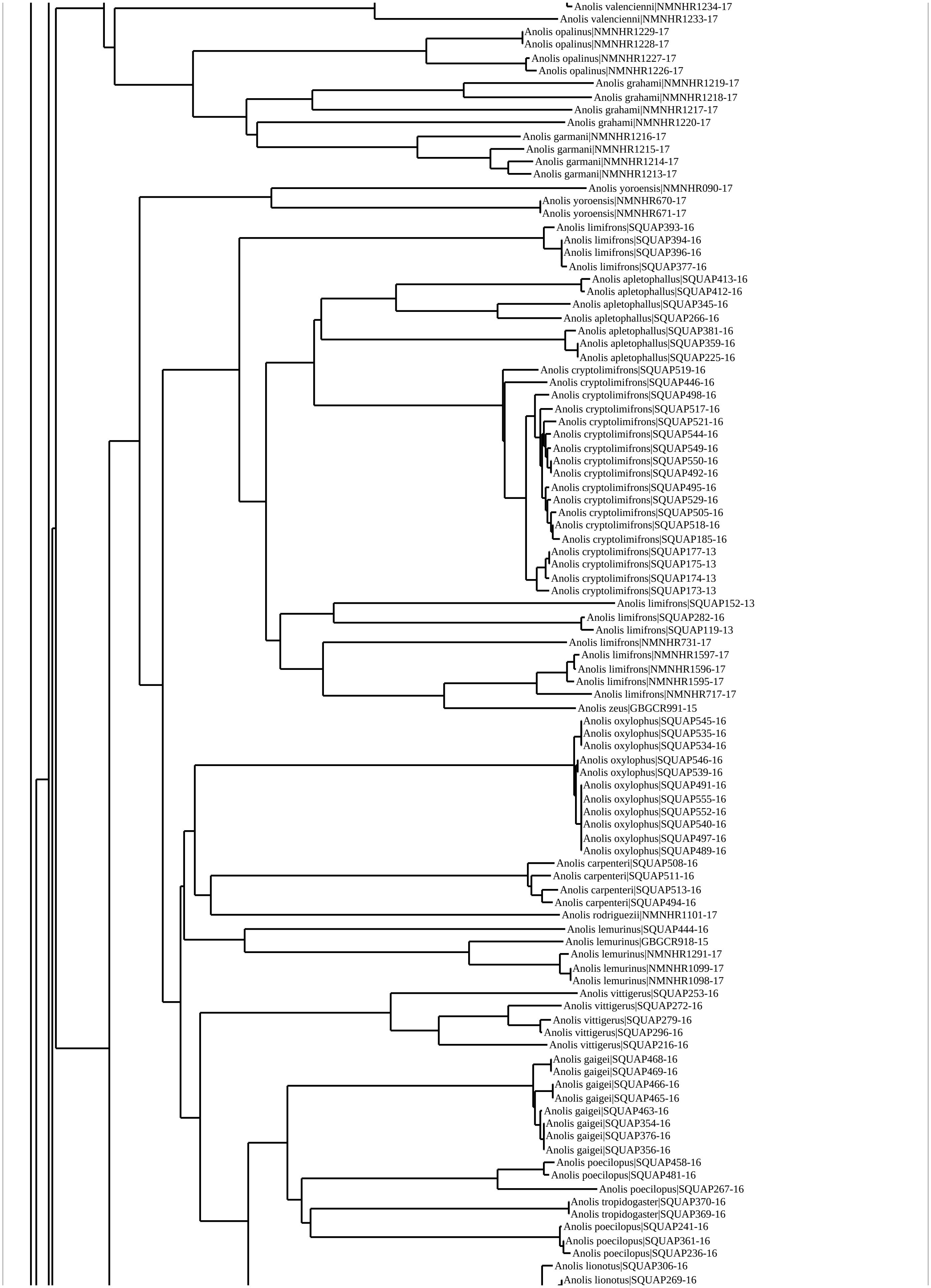
Anolis valencienni|NMNHR1234-17
Anolis valencienni|NMNHR1233-17
Anolis opalinus|NMNHR1229-17
Anolis opalinus|NMNHR1228-17
Anolis opalinus|NMNHR1227-17
Anolis opalinus|NMNHR1226-17
Anolis grahami|NMNHR1219-17
Anolis grahami|NMNHR1218-17
Anolis grahami|NMNHR1217-17
Anolis grahami|NMNHR1220-17
Anolis garmani|NMNHR1216-17
Anolis garmani|NMNHR1215-17
Anolis garmani|NMNHR1214-17
Anolis garmani|NMNHR1213-17
Anolis yoroensis|NMNHR090-17
Anolis yoroensis|NMNHR670-17
Anolis yoroensis|NMNHR671-17
Anolis limifrons|SQUAP393-16
Anolis limifrons|SQUAP394-16
Anolis limifrons|SQUAP396-16
Anolis limifrons|SQUAP377-16
Anolis apletophallus|SQUAP413-16
Anolis apletophallus|SQUAP412-16
Anolis apletophallus|SQUAP345-16
Anolis apletophallus|SQUAP266-16
Anolis apletophallus|SQUAP381-16
Anolis apletophallus|SQUAP359-16
Anolis apletophallus|SQUAP225-16
Anolis cryptolimifrons|SQUAP519-16
Anolis cryptolimifrons|SQUAP446-16
Anolis cryptolimifrons|SQUAP498-16
Anolis cryptolimifrons|SQUAP517-16
Anolis cryptolimifrons|SQUAP521-16
Anolis cryptolimifrons|SQUAP544-16
Anolis cryptolimifrons|SQUAP549-16
Anolis cryptolimifrons|SQUAP550-16
Anolis cryptolimifrons|SQUAP492-16
Anolis cryptolimifrons|SQUAP495-16
Anolis cryptolimifrons|SQUAP529-16
Anolis cryptolimifrons|SQUAP505-16
Anolis cryptolimifrons|SQUAP518-16
Anolis cryptolimifrons|SQUAP185-16
Anolis cryptolimifrons|SQUAP177-13
Anolis cryptolimifrons|SQUAP175-13
Anolis cryptolimifrons|SQUAP174-13
Anolis cryptolimifrons|SQUAP173-13
Anolis limifrons|SQUAP152-13
Anolis limifrons|SQUAP282-16
Anolis limifrons|SQUAP119-13
Anolis limifrons|NMNHR731-17
Anolis limifrons|NMNHR1597-17
Anolis limifrons|NMNHR1596-17
Anolis limifrons|NMNHR1595-17
Anolis limifrons|NMNHR717-17
Anolis zeus|GBGCR991-15
Anolis oxylophus|SQUAP545-16
Anolis oxylophus|SQUAP535-16
Anolis oxylophus|SQUAP534-16
Anolis oxylophus|SQUAP546-16
Anolis oxylophus|SQUAP539-16
Anolis oxylophus|SQUAP491-16
Anolis oxylophus|SQUAP555-16
Anolis oxylophus|SQUAP552-16
Anolis oxylophus|SQUAP540-16
Anolis oxylophus|SQUAP497-16
Anolis oxylophus|SQUAP489-16
Anolis carpenteri|SQUAP508-16
Anolis carpenteri|SQUAP511-16
Anolis carpenteri|SQUAP513-16
Anolis carpenteri|SQUAP494-16
Anolis rodriguezii|NMNHR1101-17
Anolis lemurinus|SQUAP444-16
Anolis lemurinus|GBGCR918-15
Anolis lemurinus|NMNHR1291-17
Anolis lemurinus|NMNHR1099-17
Anolis lemurinus|NMNHR1098-17
Anolis vittigerus|SQUAP253-16
Anolis vittigerus|SQUAP272-16
Anolis vittigerus|SQUAP279-16
Anolis vittigerus|SQUAP296-16
Anolis vittigerus|SQUAP216-16
Anolis gaigei|SQUAP468-16
Anolis gaigei|SQUAP469-16
Anolis gaigei|SQUAP466-16
Anolis gaigei|SQUAP465-16
Anolis gaigei|SQUAP463-16
Anolis gaigei|SQUAP354-16
Anolis gaigei|SQUAP376-16
Anolis gaigei|SQUAP356-16
Anolis poecilopus|SQUAP458-16
Anolis poecilopus|SQUAP481-16
Anolis poecilopus|SQUAP267-16
Anolis tropidogaster|SQUAP370-16
Anolis tropidogaster|SQUAP369-16
Anolis poecilopus|SQUAP241-16
Anolis poecilopus|SQUAP361-16
Anolis poecilopus|SQUAP236-16
Anolis lionotus|SQUAP306-16
Anolis lionotus|SQUAP269-16

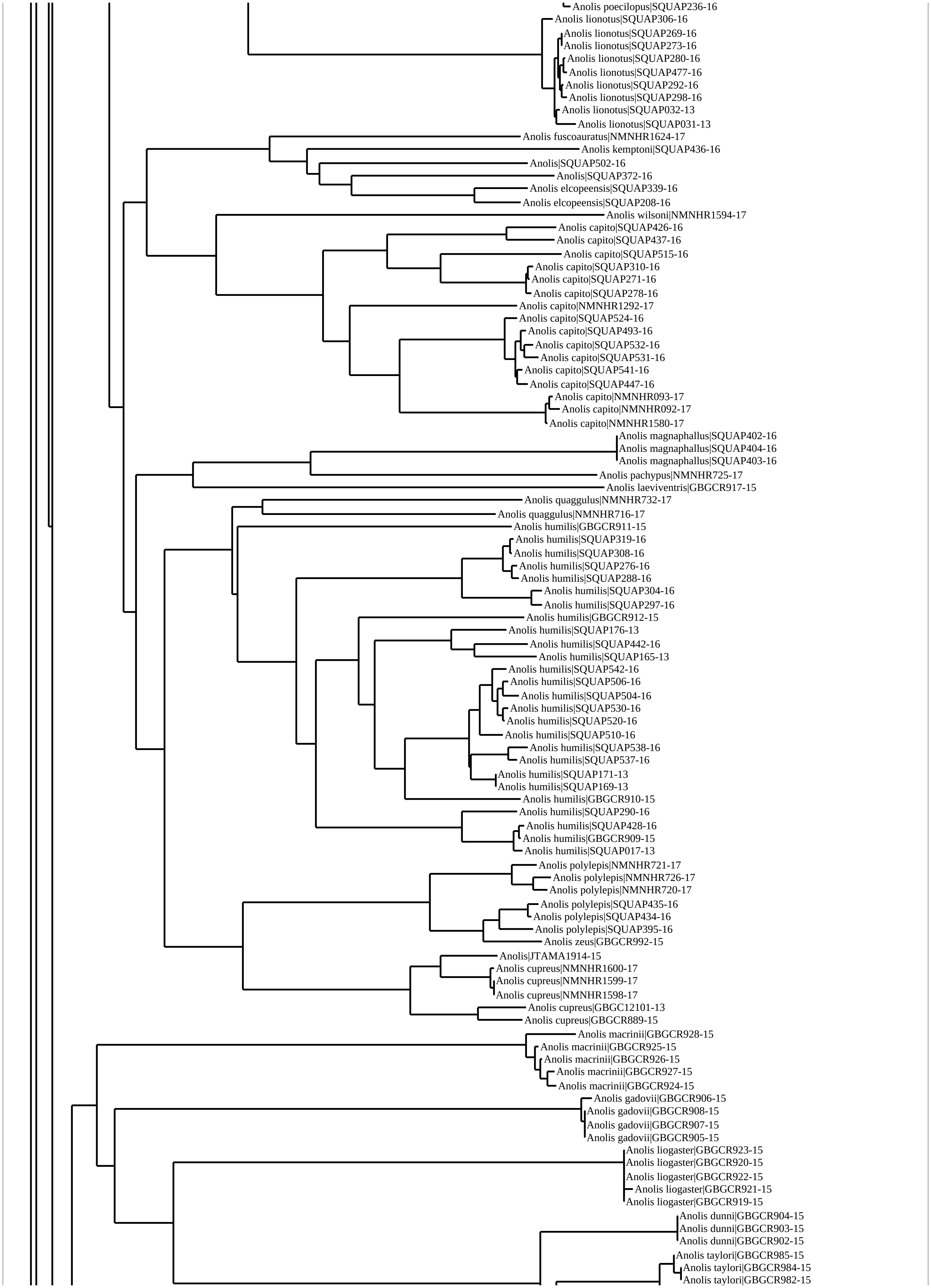
Anolis poecilopus|SQUAP236-16
Anolis lionotus|SQUAP306-16
Anolis lionotus|SQUAP269-16
Anolis lionotus|SQUAP273-16
Anolis lionotus|SQUAP280-16
Anolis lionotus|SQUAP477-16
Anolis lionotus|SQUAP292-16
Anolis lionotus|SQUAP298-16
Anolis lionotus|SQUAP032-13
Anolis lionotus|SQUAP031-13
Anolis fuscoauratus|NMNHR1624-17
Anolis kemptoni|SQUAP436-16
Anolis|SQUAP502-16
Anolis|SQUAP372-16
Anolis elcopeensis|SQUAP339-16
Anolis elcopeensis|SQUAP208-16
Anolis wilsoni|NMNHR1594-17
Anolis capito|SQUAP426-16
Anolis capito|SQUAP437-16
Anolis capito|SQUAP515-16
Anolis capito|SQUAP310-16
Anolis capito|SQUAP271-16
Anolis capito|SQUAP278-16
Anolis capito|NMNHR1292-17
Anolis capito|SQUAP524-16
Anolis capito|SQUAP493-16
Anolis capito|SQUAP532-16
Anolis capito|SQUAP531-16
Anolis capito|SQUAP541-16
Anolis capito|SQUAP447-16
Anolis capito|NMNHR093-17
Anolis capito|NMNHR092-17
Anolis capito|NMNHR1580-17
Anolis magnaphallus|SQUAP402-16
Anolis magnaphallus|SQUAP404-16
Anolis magnaphallus|SQUAP403-16
Anolis pachypus|NMNHR725-17
Anolis laeviventris|GBGCR917-15
Anolis quaggulus|NMNHR732-17
Anolis quaggulus|NMNHR716-17
Anolis humilis|GBGCR911-15
Anolis humilis|SQUAP319-16
Anolis humilis|SQUAP308-16
Anolis humilis|SQUAP276-16
Anolis humilis|SQUAP288-16
Anolis humilis|SQUAP304-16
Anolis humilis|SQUAP297-16
Anolis humilis|GBGCR912-15
Anolis humilis|SQUAP176-13
Anolis humilis|SQUAP442-16
Anolis humilis|SQUAP165-13
Anolis humilis|SQUAP542-16
Anolis humilis|SQUAP506-16
Anolis humilis|SQUAP504-16
Anolis humilis|SQUAP530-16
Anolis humilis|SQUAP520-16
Anolis humilis|SQUAP510-16
Anolis humilis|SQUAP538-16
Anolis humilis|SQUAP537-16
Anolis humilis|SQUAP171-13
Anolis humilis|SQUAP169-13
Anolis humilis|GBGCR910-15
Anolis humilis|SQUAP290-16
Anolis humilis|SQUAP428-16
Anolis humilis|GBGCR909-15
Anolis humilis|SQUAP017-13
Anolis polylepis|NMNHR721-17
Anolis polylepis|NMNHR726-17
Anolis polylepis|NMNHR720-17
Anolis polylepis|SQUAP435-16
Anolis polylepis|SQUAP434-16
Anolis polylepis|SQUAP395-16
Anolis zeus|GBGCR992-15
Anolis|JTAMA1914-15
Anolis cupreus|NMNHR1600-17
Anolis cupreus|NMNHR1599-17
Anolis cupreus|NMNHR1598-17
Anolis cupreus|GBGC12101-13
Anolis cupreus|GBGCR889-15
Anolis macrinii|GBGCR928-15
Anolis macrinii|GBGCR925-15
Anolis macrinii|GBGCR926-15
Anolis macrinii|GBGCR927-15
Anolis macrinii|GBGCR924-15
Anolis gadovii|GBGCR906-15
Anolis gadovii|GBGCR908-15
Anolis gadovii|GBGCR907-15
Anolis gadovii|GBGCR905-15
Anolis liogaster|GBGCR923-15
Anolis liogaster|GBGCR920-15
Anolis liogaster|GBGCR922-15
Anolis liogaster|GBGCR921-15
Anolis liogaster|GBGCR919-15
Anolis dunni|GBGCR904-15
Anolis dunni|GBGCR903-15
Anolis dunni|GBGCR902-15
Anolis taylori|GBGCR985-15
Anolis taylori|GBGCR984-15
Anolis taylori|GBGCR982-15

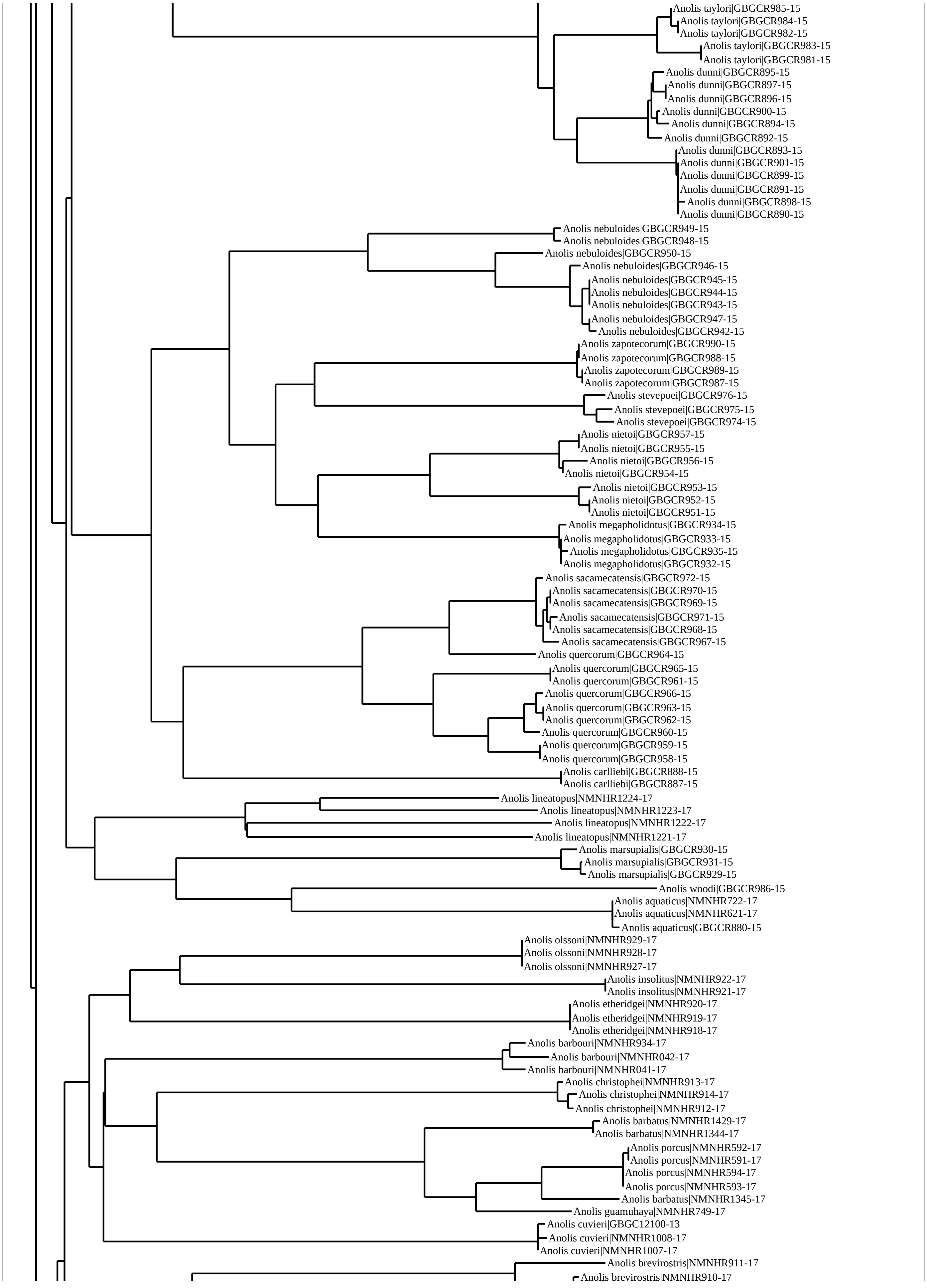
Anolis taylori|GBGCR985-15
Anolis taylori|GBGCR984-15
Anolis taylori|GBGCR982-15
Anolis taylori|GBGCR983-15
Anolis taylori|GBGCR981-15
Anolis dunni|GBGCR895-15
Anolis dunni|GBGCR897-15
Anolis dunni|GBGCR896-15
Anolis dunni|GBGCR900-15
Anolis dunni|GBGCR894-15
Anolis dunni|GBGCR892-15
Anolis dunni|GBGCR893-15
Anolis dunni|GBGCR901-15
Anolis dunni|GBGCR899-15
Anolis dunni|GBGCR891-15
Anolis dunni|GBGCR898-15
Anolis dunni|GBGCR890-15
Anolis nebuloides|GBGCR949-15
Anolis nebuloides|GBGCR948-15
Anolis nebuloides|GBGCR950-15
Anolis nebuloides|GBGCR946-15
Anolis nebuloides|GBGCR945-15
Anolis nebuloides|GBGCR944-15
Anolis nebuloides|GBGCR943-15
Anolis nebuloides|GBGCR947-15
Anolis nebuloides|GBGCR942-15
Anolis zapotecorum|GBGCR990-15
Anolis zapotecorum|GBGCR988-15
Anolis zapotecorum|GBGCR989-15
Anolis zapotecorum|GBGCR987-15
Anolis stevepoei|GBGCR976-15
Anolis stevepoei|GBGCR975-15
Anolis stevepoei|GBGCR974-15
Anolis nietoi|GBGCR957-15
Anolis nietoi|GBGCR955-15
Anolis nietoi|GBGCR956-15
Anolis nietoi|GBGCR954-15
Anolis nietoi|GBGCR953-15
Anolis nietoi|GBGCR952-15
Anolis nietoi|GBGCR951-15
Anolis megapholidotus|GBGCR934-15
Anolis megapholidotus|GBGCR933-15
Anolis megapholidotus|GBGCR935-15
Anolis megapholidotus|GBGCR932-15
Anolis sacamecatensis|GBGCR972-15
Anolis sacamecatensis|GBGCR970-15
Anolis sacamecatensis|GBGCR969-15
Anolis sacamecatensis|GBGCR971-15
Anolis sacamecatensis|GBGCR968-15
Anolis sacamecatensis|GBGCR967-15
Anolis quercorum|GBGCR964-15
Anolis quercorum|GBGCR965-15
Anolis quercorum|GBGCR961-15
Anolis quercorum|GBGCR966-15
Anolis quercorum|GBGCR963-15
Anolis quercorum|GBGCR962-15
Anolis quercorum|GBGCR960-15
Anolis quercorum|GBGCR959-15
Anolis quercorum|GBGCR958-15
Anolis carlliebi|GBGCR888-15
Anolis carlliebi|GBGCR887-15
Anolis lineatopus|NMNHR1224-17
Anolis lineatopus|NMNHR1223-17
Anolis lineatopus|NMNHR1222-17
Anolis lineatopus|NMNHR1221-17
Anolis marsupialis|GBGCR930-15
Anolis marsupialis|GBGCR931-15
Anolis marsupialis|GBGCR929-15
Anolis woodi|GBGCR986-15
Anolis aquaticus|NMNHR722-17
Anolis aquaticus|NMNHR621-17
Anolis aquaticus|GBGCR880-15
Anolis olssoni|NMNHR929-17
Anolis olssoni|NMNHR928-17
Anolis olssoni|NMNHR927-17
Anolis insolitus|NMNHR922-17
Anolis insolitus|NMNHR921-17
Anolis etheridgei|NMNHR920-17
Anolis etheridgei|NMNHR919-17
Anolis etheridgei|NMNHR918-17
Anolis barbouri|NMNHR934-17
Anolis barbouri|NMNHR042-17
Anolis barbouri|NMNHR041-17
Anolis christophei|NMNHR913-17
Anolis christophei|NMNHR914-17
Anolis christophei|NMNHR912-17
Anolis barbatus|NMNHR1429-17
Anolis barbatus|NMNHR1344-17
Anolis porcus|NMNHR592-17
Anolis porcus|NMNHR591-17
Anolis porcus|NMNHR594-17
Anolis porcus|NMNHR593-17
Anolis barbatus|NMNHR1345-17
Anolis guamuhaya|NMNHR749-17
Anolis cuvieri|GBGC12100-13
Anolis cuvieri|NMNHR1008-17
Anolis cuvieri|NMNHR1007-17
Anolis brevirostris|NMNHR911-17
Anolis brevirostris|NMNHR910-17

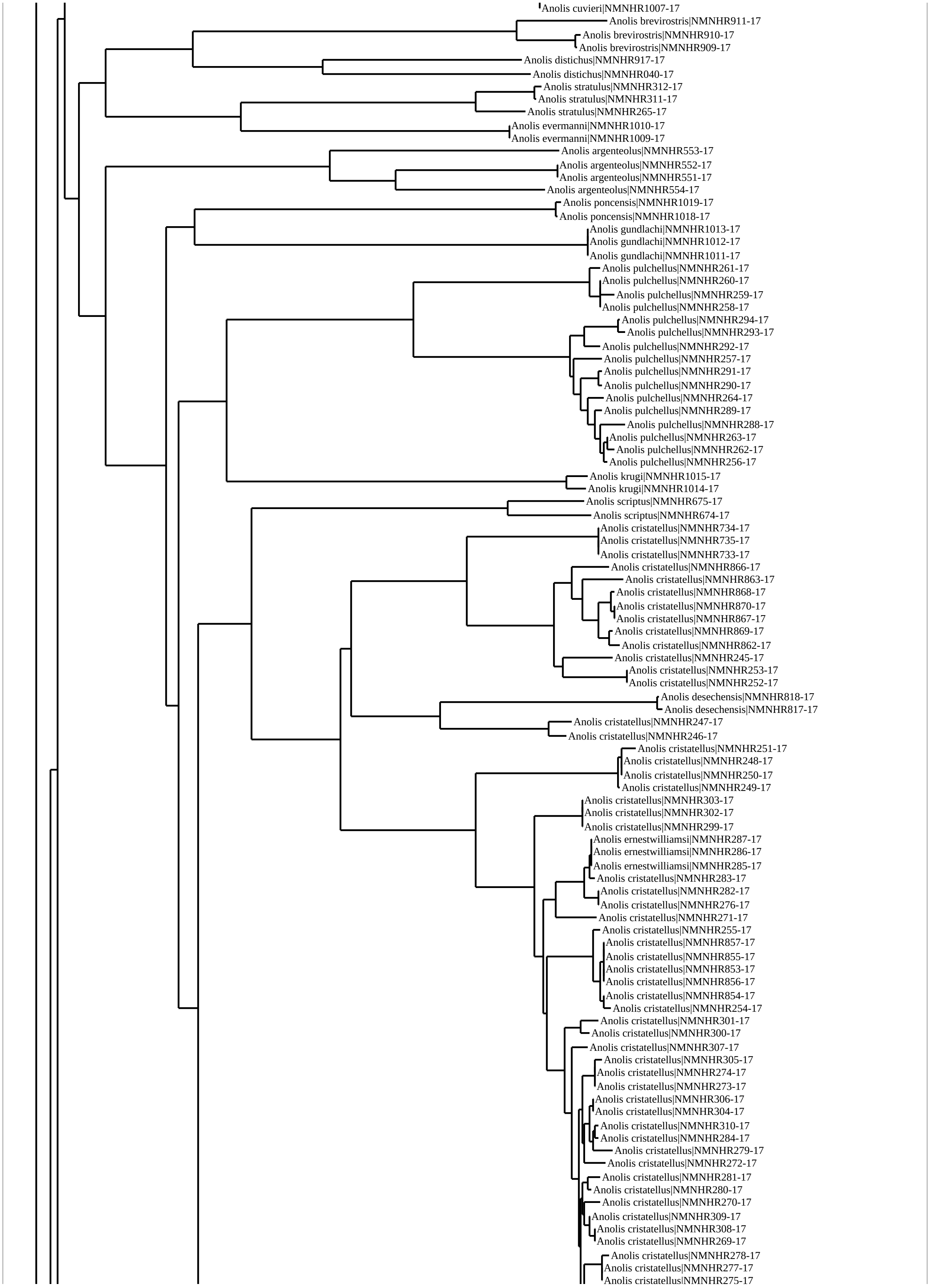
Anolis cuvieri|NMNHR1007-17
Anolis brevirostris|NMNHR911-17
Anolis brevirostris|NMNHR910-17
Anolis brevirostris|NMNHR909-17
Anolis distichus|NMNHR917-17
Anolis distichus|NMNHR040-17
Anolis stratulus|NMNHR312-17
Anolis stratulus|NMNHR311-17
Anolis stratulus|NMNHR265-17
Anolis evermanni|NMNHR1010-17
Anolis evermanni|NMNHR1009-17
Anolis argenteolus|NMNHR553-17
Anolis argenteolus|NMNHR552-17
Anolis argenteolus|NMNHR551-17
Anolis argenteolus|NMNHR554-17
Anolis poncensis|NMNHR1019-17
Anolis poncensis|NMNHR1018-17
Anolis gundlachi|NMNHR1013-17
Anolis gundlachi|NMNHR1012-17
Anolis gundlachi|NMNHR1011-17
Anolis pulchellus|NMNHR261-17
Anolis pulchellus|NMNHR260-17
Anolis pulchellus|NMNHR259-17
Anolis pulchellus|NMNHR258-17
Anolis pulchellus|NMNHR294-17
Anolis pulchellus|NMNHR293-17
Anolis pulchellus|NMNHR292-17
Anolis pulchellus|NMNHR257-17
Anolis pulchellus|NMNHR291-17
Anolis pulchellus|NMNHR290-17
Anolis pulchellus|NMNHR264-17
Anolis pulchellus|NMNHR289-17
Anolis pulchellus|NMNHR288-17
Anolis pulchellus|NMNHR263-17
Anolis pulchellus|NMNHR262-17
Anolis pulchellus|NMNHR256-17
Anolis krugi|NMNHR1015-17
Anolis krugi|NMNHR1014-17
Anolis scriptus|NMNHR675-17
Anolis scriptus|NMNHR674-17
Anolis cristatellus|NMNHR734-17
Anolis cristatellus|NMNHR735-17
Anolis cristatellus|NMNHR733-17
Anolis cristatellus|NMNHR866-17
Anolis cristatellus|NMNHR863-17
Anolis cristatellus|NMNHR868-17
Anolis cristatellus|NMNHR870-17
Anolis cristatellus|NMNHR867-17
Anolis cristatellus|NMNHR869-17
Anolis cristatellus|NMNHR862-17
Anolis cristatellus|NMNHR245-17
Anolis cristatellus|NMNHR253-17
Anolis cristatellus|NMNHR252-17
Anolis desechensis|NMNHR818-17
Anolis desechensis|NMNHR817-17
Anolis cristatellus|NMNHR247-17
Anolis cristatellus|NMNHR246-17
Anolis cristatellus|NMNHR251-17
Anolis cristatellus|NMNHR248-17
Anolis cristatellus|NMNHR250-17
Anolis cristatellus|NMNHR249-17
Anolis cristatellus|NMNHR303-17
Anolis cristatellus|NMNHR302-17
Anolis cristatellus|NMNHR299-17
Anolis ernestwilliamsi|NMNHR287-17
Anolis ernestwilliamsi|NMNHR286-17
Anolis ernestwilliamsi|NMNHR285-17
Anolis cristatellus|NMNHR283-17
Anolis cristatellus|NMNHR282-17
Anolis cristatellus|NMNHR276-17
Anolis cristatellus|NMNHR271-17
Anolis cristatellus|NMNHR255-17
Anolis cristatellus|NMNHR857-17
Anolis cristatellus|NMNHR855-17
Anolis cristatellus|NMNHR853-17
Anolis cristatellus|NMNHR856-17
Anolis cristatellus|NMNHR854-17
Anolis cristatellus|NMNHR254-17
Anolis cristatellus|NMNHR301-17
Anolis cristatellus|NMNHR300-17
Anolis cristatellus|NMNHR307-17
Anolis cristatellus|NMNHR305-17
Anolis cristatellus|NMNHR274-17
Anolis cristatellus|NMNHR273-17
Anolis cristatellus|NMNHR306-17
Anolis cristatellus|NMNHR304-17
Anolis cristatellus|NMNHR310-17
Anolis cristatellus|NMNHR284-17
Anolis cristatellus|NMNHR279-17
Anolis cristatellus|NMNHR272-17
Anolis cristatellus|NMNHR281-17
Anolis cristatellus|NMNHR280-17
Anolis cristatellus|NMNHR270-17
Anolis cristatellus|NMNHR309-17
Anolis cristatellus|NMNHR308-17
Anolis cristatellus|NMNHR269-17
Anolis cristatellus|NMNHR278-17
Anolis cristatellus|NMNHR277-17
Anolis cristatellus|NMNHR275-17

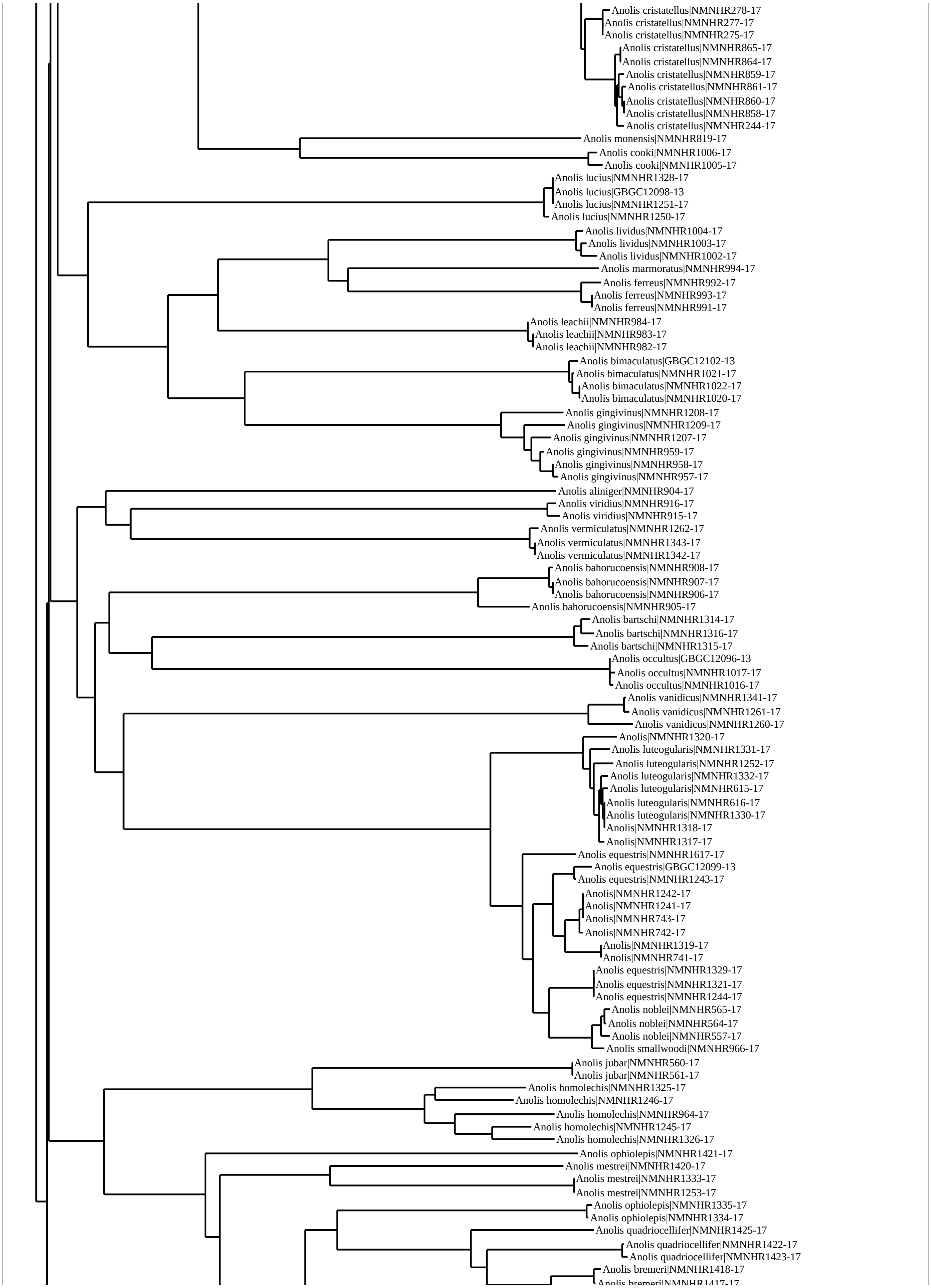
Anolis cristatellus|NMNHR278-17
Anolis cristatellus|NMNHR277-17
Anolis cristatellus|NMNHR275-17
Anolis cristatellus|NMNHR865-17
Anolis cristatellus|NMNHR864-17
Anolis cristatellus|NMNHR859-17
Anolis cristatellus|NMNHR861-17
Anolis cristatellus|NMNHR860-17
Anolis cristatellus|NMNHR858-17
Anolis cristatellus|NMNHR244-17
Anolis monensis|NMNHR819-17
Anolis cooki|NMNHR1006-17
Anolis cooki|NMNHR1005-17
Anolis lucius|NMNHR1328-17
Anolis lucius|GBGC12098-13
Anolis lucius|NMNHR1251-17
Anolis lucius|NMNHR1250-17
Anolis lividus|NMNHR1004-17
Anolis lividus|NMNHR1003-17
Anolis lividus|NMNHR1002-17
Anolis marmoratus|NMNHR994-17
Anolis ferreus|NMNHR992-17
Anolis ferreus|NMNHR993-17
Anolis ferreus|NMNHR991-17
Anolis leachii|NMNHR984-17
Anolis leachii|NMNHR983-17
Anolis leachii|NMNHR982-17
Anolis bimaculatus|GBGC12102-13
Anolis bimaculatus|NMNHR1021-17
Anolis bimaculatus|NMNHR1022-17
Anolis bimaculatus|NMNHR1020-17
Anolis gingivinus|NMNHR1208-17
Anolis gingivinus|NMNHR1209-17
Anolis gingivinus|NMNHR1207-17
Anolis gingivinus|NMNHR959-17
Anolis gingivinus|NMNHR958-17
Anolis gingivinus|NMNHR957-17
Anolis aliniger|NMNHR904-17
Anolis viridius|NMNHR916-17
Anolis viridius|NMNHR915-17
Anolis vermiculatus|NMNHR1262-17
Anolis vermiculatus|NMNHR1343-17
Anolis vermiculatus|NMNHR1342-17
Anolis bahorucoensis|NMNHR908-17
Anolis bahorucoensis|NMNHR907-17
Anolis bahorucoensis|NMNHR906-17
Anolis bahorucoensis|NMNHR905-17
Anolis bartschi|NMNHR1314-17
Anolis bartschi|NMNHR1316-17
Anolis bartschi|NMNHR1315-17
Anolis occultus|GBGC12096-13
Anolis occultus|NMNHR1017-17
Anolis occultus|NMNHR1016-17
Anolis vanidicus|NMNHR1341-17
Anolis vanidicus|NMNHR1261-17
Anolis vanidicus|NMNHR1260-17
Anolis|NMNHR1320-17
Anolis luteogularis|NMNHR1331-17
Anolis luteogularis|NMNHR1252-17
Anolis luteogularis|NMNHR1332-17
Anolis luteogularis|NMNHR615-17
Anolis luteogularis|NMNHR616-17
Anolis luteogularis|NMNHR1330-17
Anolis|NMNHR1318-17
Anolis|NMNHR1317-17
Anolis equestris|NMNHR1617-17
Anolis equestris|GBGC12099-13
Anolis equestris|NMNHR1243-17
Anolis|NMNHR1242-17
Anolis|NMNHR1241-17
Anolis|NMNHR743-17
Anolis|NMNHR742-17
Anolis|NMNHR1319-17
Anolis|NMNHR741-17
Anolis equestris|NMNHR1329-17
Anolis equestris|NMNHR1321-17
Anolis equestris|NMNHR1244-17
Anolis noblei|NMNHR565-17
Anolis noblei|NMNHR564-17
Anolis noblei|NMNHR557-17
Anolis smallwoodi|NMNHR966-17
Anolis jubar|NMNHR560-17
Anolis jubar|NMNHR561-17
Anolis homolechis|NMNHR1325-17
Anolis homolechis|NMNHR1246-17
Anolis homolechis|NMNHR964-17
Anolis homolechis|NMNHR1245-17
Anolis homolechis|NMNHR1326-17
Anolis ophiolepis|NMNHR1421-17
Anolis mestrei|NMNHR1420-17
Anolis mestrei|NMNHR1333-17
Anolis mestrei|NMNHR1253-17
Anolis ophiolepis|NMNHR1335-17
Anolis ophiolepis|NMNHR1334-17
Anolis quadriocellifer|NMNHR1425-17
Anolis quadriocellifer|NMNHR1422-17
Anolis quadriocellifer|NMNHR1423-17
Anolis bremeri|NMNHR1418-17
Anolis bremeri|NMNHR1417-17

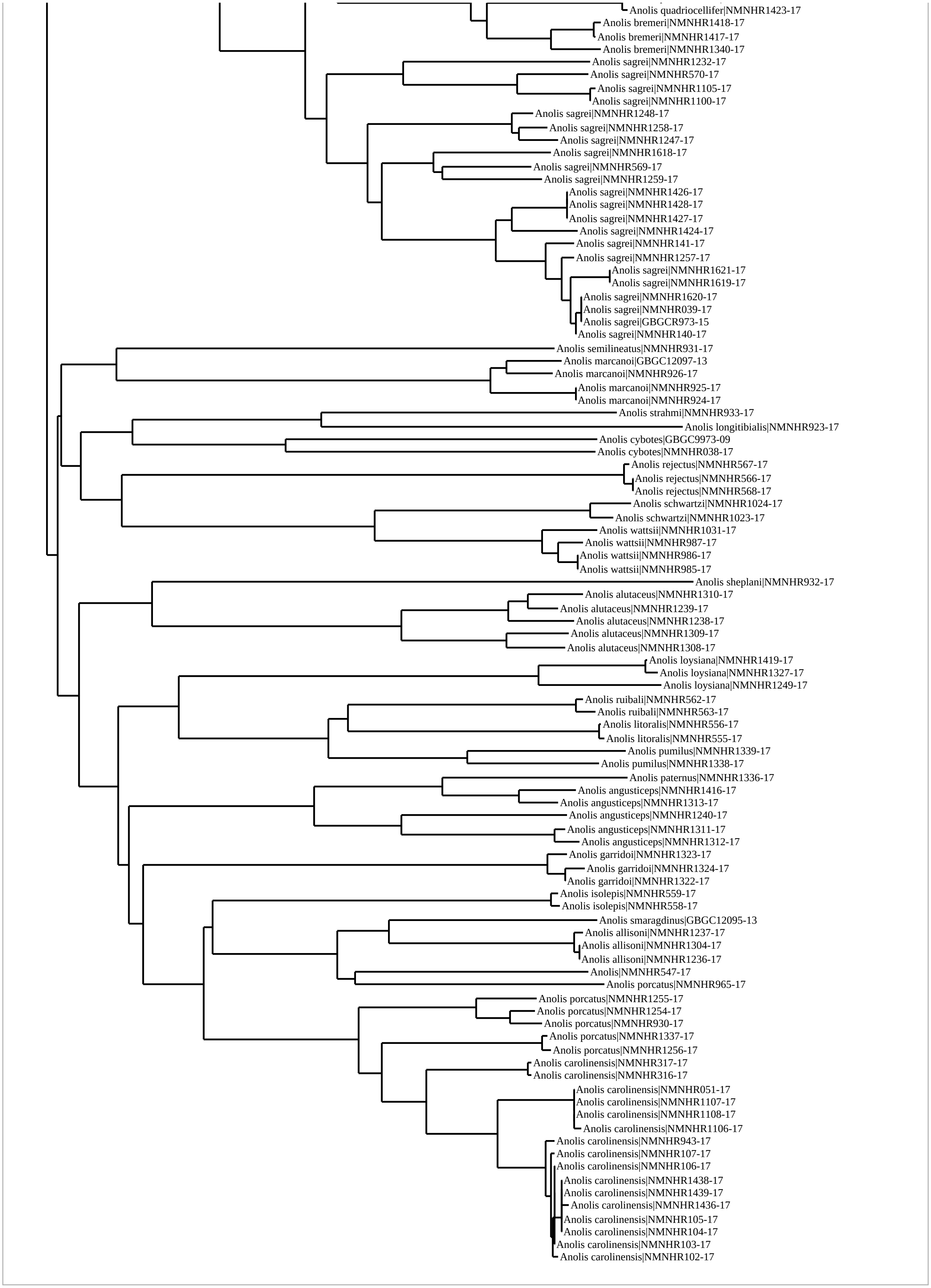
Anolis quadriocellifer|NMNHR1423-17
Anolis bremeri|NMNHR1418-17
Anolis bremeri|NMNHR1417-17
Anolis bremeri|NMNHR1340-17
Anolis sagrei|NMNHR1232-17
Anolis sagrei|NMNHR570-17
Anolis sagrei|NMNHR1105-17
Anolis sagrei|NMNHR1100-17
Anolis sagrei|NMNHR1248-17
Anolis sagrei|NMNHR1258-17
Anolis sagrei|NMNHR1247-17
Anolis sagrei|NMNHR1618-17
Anolis sagrei|NMNHR569-17
Anolis sagrei|NMNHR1259-17
Anolis sagrei|NMNHR1426-17
Anolis sagrei|NMNHR1428-17
Anolis sagrei|NMNHR1427-17
Anolis sagrei|NMNHR1424-17
Anolis sagrei|NMNHR141-17
Anolis sagrei|NMNHR1257-17
Anolis sagrei|NMNHR1621-17
Anolis sagrei|NMNHR1619-17
Anolis sagrei|NMNHR1620-17
Anolis sagrei|NMNHR039-17
Anolis sagrei|GBGCR973-15
Anolis sagrei|NMNHR140-17
Anolis semilineatus|NMNHR931-17
Anolis marcanoi|GBGC12097-13
Anolis marcanoi|NMNHR926-17
Anolis marcanoi|NMNHR925-17
Anolis marcanoi|NMNHR924-17
Anolis strahmi|NMNHR933-17
Anolis longitibialis|NMNHR923-17
Anolis cybotes|GBGC9973-09
Anolis cybotes|NMNHR038-17
Anolis rejectus|NMNHR567-17
Anolis rejectus|NMNHR566-17
Anolis rejectus|NMNHR568-17
Anolis schwartzi|NMNHR1024-17
Anolis schwartzi|NMNHR1023-17
Anolis wattsii|NMNHR1031-17
Anolis wattsii|NMNHR987-17
Anolis wattsii|NMNHR986-17
Anolis wattsii|NMNHR985-17
Anolis sheplani|NMNHR932-17
Anolis alutaceus|NMNHR1310-17
Anolis alutaceus|NMNHR1239-17
Anolis alutaceus|NMNHR1238-17
Anolis alutaceus|NMNHR1309-17
Anolis alutaceus|NMNHR1308-17
Anolis loysiana|NMNHR1419-17
Anolis loysiana|NMNHR1327-17
Anolis loysiana|NMNHR1249-17
Anolis ruibali|NMNHR562-17
Anolis ruibali|NMNHR563-17
Anolis litoralis|NMNHR556-17
Anolis litoralis|NMNHR555-17
Anolis pumilus|NMNHR1339-17
Anolis pumilus|NMNHR1338-17
Anolis paternus|NMNHR1336-17
Anolis angusticeps|NMNHR1416-17
Anolis angusticeps|NMNHR1313-17
Anolis angusticeps|NMNHR1240-17
Anolis angusticeps|NMNHR1311-17
Anolis angusticeps|NMNHR1312-17
Anolis garridoi|NMNHR1323-17
Anolis garridoi|NMNHR1324-17
Anolis garridoi|NMNHR1322-17
Anolis isolepis|NMNHR559-17
Anolis isolepis|NMNHR558-17
Anolis smaragdinus|GBGC12095-13
Anolis allisoni|NMNHR1237-17
Anolis allisoni|NMNHR1304-17
Anolis allisoni|NMNHR1236-17
Anolis|NMNHR547-17
Anolis porcatus|NMNHR965-17
Anolis porcatus|NMNHR1255-17
Anolis porcatus|NMNHR1254-17
Anolis porcatus|NMNHR930-17
Anolis porcatus|NMNHR1337-17
Anolis porcatus|NMNHR1256-17
Anolis carolinensis|NMNHR317-17
Anolis carolinensis|NMNHR316-17
Anolis carolinensis|NMNHR051-17
Anolis carolinensis|NMNHR1107-17
Anolis carolinensis|NMNHR1108-17
Anolis carolinensis|NMNHR1106-17
Anolis carolinensis|NMNHR943-17
Anolis carolinensis|NMNHR107-17
Anolis carolinensis|NMNHR106-17
Anolis carolinensis|NMNHR1438-17
Anolis carolinensis|NMNHR1439-17
Anolis carolinensis|NMNHR1436-17
Anolis carolinensis|NMNHR105-17
Anolis carolinensis|NMNHR104-17
Anolis carolinensis|NMNHR103-17
Anolis carolinensis|NMNHR102-17
